# *Track-A-Worm 2.0*: A Software Suite for Quantifying Properties of *C. elegans* Locomotion, Bending, Sleep, and Action Potentials

**DOI:** 10.1523/ENEURO.0224-25.2025

**Published:** 2025-08-13

**Authors:** Kiranmayi Vedantham, Longgang Niu, Ryan Ma, Liam Connelly, Anusha Nagella, Sijie Jason Wang, Zhao-Wen Wang

**Affiliations:** ^1^Department of Neuroscience, University of Connecticut School of Medicine, Farmington, Connecticut 06030; ^2^Health Research Program, University of Connecticut, Storrs, Connecticut 06030; ^3^MD Program, University of Connecticut School of Medicine, Farmington, Connecticut 06030

**Keywords:** action potential, *C. elegans*, locomotion, MATLAB, open-source, sleep

## Abstract

Comparative analyses of locomotor behavior and cellular electrical properties between wild-type and mutant *Caenorhabditis elegans* are crucial for exploring the gene basis of behaviors and the underlying cellular mechanisms. Although many tools have been developed by research labs and companies, their application is often hindered by implementation difficulties or lack of features specifically suited for *C. elegans*. Our system addresses these challenges with three key components: *WormTracker*, *SleepTracker*, and *Action Potential (AP) Analyzer*. *WormTracker* accurately quantifies a comprehensive set of locomotor and body bending metrics, incorporates user-identified dorsal and ventral orientation based on microscopic observation, continuously tracks the animal using a motorized stage, and seamlessly integrates external devices, such as a light source for optogenetic stimulation. *SleepTracker* detects and quantifies sleep-like behavior in freely moving animals. *AP Analyzer* assesses the resting membrane potential, afterhyperpolarization level, and various AP properties, including threshold, amplitude, mid-peak width, rise and decay times, and maximum and minimum slopes. Importantly, it addresses the challenge of AP threshold quantification posed by the absence of a preupstroke inflection point. This system is potentially a valuable tool for many *C. elegans* research labs due to its powerful functionality and ease of implementation.

## Significance Statement

The nematode *Caenorhabditis elegans* is a premier model for dissecting molecular, cellular, and neural circuit mechanisms of behavior. Such studies often require precise quantification of how genetic perturbations alter locomotion, sleep, and action potentials. However, no integrated system—open-source or commercial—exists to perform these multimodal analyses. Our open-source software suite bridges this gap by enabling high-resolution analysis of locomotion, body bending, and sleep in freely moving animals, as well as detailed quantification of action potential properties, including metrics that are challenging to assess with existing commercial tools. Designed for ease of use and implementation, this system offers a versatile and accessible solution for the *C. elegans* research community.

## Introduction

*Caenorhabditis elegans* is widely used to study the gene bases of behavior. Many mutations can alter *C. elegans* locomotion and body bending behavior, which typically require machine vision for quantitative analysis, prompting the development of numerous automated tracking systems (Feng et al., 2004; Tsibidis and Tavernarakis, 2007; Ramot et al., 2008; Swierczek et al., 2011; Wen et al., 2012; Kwon et al., 2013, 2015; Wang and Wang, 2013; Yemini et al., 2013; Machino et al., 2014; Perni et al., 2018; Javer et al., 2018a,b; Barlow et al., 2022; Bonnard et al., 2022; Puchalt et al., 2022; Deserno and Bozek, 2023). However, most systems are limited to in-house use or lack features like continuous tracking or dorsal/ventral differentiation.

A few systems have been developed for public use. Notably, *WormLab* (Roussel et al., 2014), a commercial multi-worm tracker from MBF Bioscience, and *Tierpsy Tracker*, an open-source multi-worm tracker (Javer et al., 2018a,b), are well known. However, they often cannot continuously track the same worm for extended time due to the absence of a motorized stage and cannot differentiate ventral and dorsal sides. *Tierpsy Tracker* also runs in a *Docker* environment, requiring users to install and configure the software, manage dependencies, and troubleshoot compatibility issues, which presents a steep learning curve. *Worm Tracker 2.0* (Yemini et al., 2013), an open-source single-worm tracker, is no longer available (https://worm-tracking.sourceforge.net/).

Beyond locomotion, *C. elegans* exhibits developmentally timed sleep (Raizen et al., 2008). Current sleep assays, however, often rely on physical restraint in microfluidic devices, which may alter naturalistic movement. A system for tracking freely moving animals would address this gap.

*C. elegans* body-wall muscle cells and some neurons can fire action potentials (APs; Gao and Zhen, 2011; Liu et al., 2011, 2018; Jiang et al., 2022). These APs, unlike those in mammalian neurons and muscle cells, lack a discernible inflection point in the rising phase. As a result, investigators must manually quantify the threshold, which can introduce inconsistencies. Since AP threshold is often used in quantifying AP amplitude, rise time, and afterhyperpolarization (AHP) amplitude, it is crucial to have an objective assessment of AP threshold.

*Track-A-Worm* is an open-source, single-worm tracker based on the MATLAB. The original version (Wang and Wang, 2013) could not quantify body length or curvature, nor could it distinguish between animal's ventral and dorsal sides. It also cannot track worms on a bacterial lawn or control external devices for applications such as optogenetic stimulation. Moreover, the absence of video tutorials made it difficult for readers to fully appreciate the system's capabilities.

Here, we present *Track-A-Worm 2.0*, an open-source system quantifying detailed locomotion and body bending behavior. It addresses all limitations of the original version, conveniently measures sleep duration in freely moving worms, and quantifies AP threshold using a definable approach, along with other AP properties in *C. elegans*. Importantly, video tutorials and sample recordings are provided, allowing users to evaluate whether this system meets their research needs.

## Materials and Methods

### Software

The software suite is available in two versions: a standalone version (Extended Data 1) and a MATLAB-dependent version (Extended Data 2). The standalone version can be used directly with the hardware described below, referred to as “standard hardware.” The MATLAB-dependent version is also configured for the standard hardware but offers the flexibility to configure custom hardware (requiring basic MATLAB knowledge).

10.1523/ENEURO.0224-25.2025.d1 Extended Data 1 **Standalone version of the software suite.** This executable file allows users to install the standalone version of the software suite. It does not need MATLAB to run. Instructions for installation are described in Extended Data 3. Download Extended Data 1, ZIP file.

10.1523/ENEURO.0224-25.2025.d2 Extended Data 2 **MATLAB codes for the software suite.** This folder contains the MATLAB codes of the software. Users can run the software and configure none “standard” hardware with MATLAB (R2021a or later) installed on their computers. Download Extended Data 2, ZIP file.

### Code accessibility

The code/software described in the paper is freely available online at https://github.com/wormlabuchc/TrackAWorm. The code is available as Extended Data 2.

### Hardware

**Figure 1.**
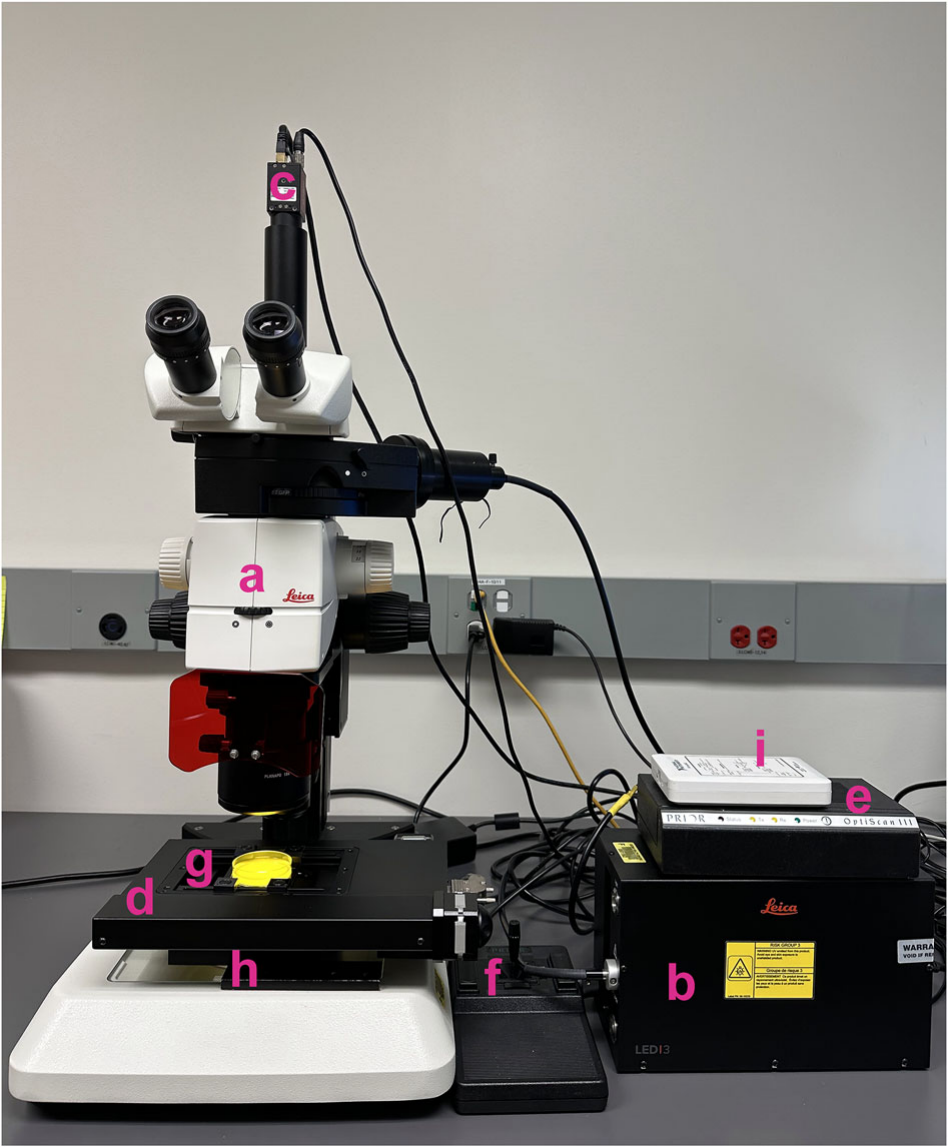
*WormTracker* hardware components. Shown are the hardware configuration of our system, including a fluorescence stereomicroscope (M165 FC, Leica; ***a***) with a fluorescence light source (LED3, Leica; ***b***), a C-mount CMOS camera (Mako G-040B, Allied Vision; ***c***), a motorized stage (OptiScan ES111; ***d***) with a stage controller (ES11), a joystick (CS1521DP; ***f***), a universal specimen holder (H473; ***g***), and a stage mounting bracket (H413; ***h***; all from Prior Scientific) and an external device controller (myDAQ, National Instruments; ***i***). The WormTracker software is preconfigured to work with components ***c***, ***d***, and ***i***. Components ***b*** and ***a*** 520 nm long-pass cutoff filter (Y52, Hoya Corporation) are required only for optogenetic stimulation. The Petri dish is 60 mm in diameter. The yellow color in the Petri dish is due to light from the microscope base filtered through the long-pass filter. The camera and stage must be oriented as shown to ensure proper stage tracking.

The required hardware components are shown in Figure 1. Instructions for their setup are described below.

#### Basic hardware

The basic hardware includes a desktop or laptop computer running Windows 10 or 11 (64 bit) with a GigE (Gigabit Ethernet) port for the camera described below and a stereomicroscope with an illuminated base. While most stereomicroscopes commonly used in *C. elegans* research laboratories are suitable for this application, those with good optics and a base that allows adjustable angled illumination offer advantages in image quality.

#### Additional hardware for *WormTracker*

##### Motorized stage

OptiScan ES111, including a stage controller (ES11), a universal specimen holder (H473), a stage mounting bracket (H413), and a joystick (CS1521DP; all from Prior Scientific; Table 1). The joystick is optional. You may want to request the manufacturer to reduce the stage mounting bracket height from the standard 8.0 cm to 2.0–2.5 cm to allow better illumination from the microscope base. This stage has a travel range of 125 mm × 75 mm with a minimum step size of 1 µm. If you choose to use a different motorized stage, ensure that its travel distances (in both the *x* and *y* directions) are significantly longer than the diameter of the Petri dish used for worm tracking. Additionally, ensure that the stage position can be adjusted promptly so that adjustments can occur and complete between image acquisitions.

**Table 1.** Hardware components and sources

Component	Model or product #	Supplier	Product URL
Motorized stage	OptiScan ES111	Prior Scientific	https://www.prior.com/product/optiscan-es111
Stage controller	OptiScan ES11	Prior Scientific	https://www.prior.com/product/optiscan-es11-controller
Universal specimen holder	H473	Prior Scientific	https://www.prior.com/product/h473
Stage mounting bracket	H413	Prior Scientific	N/A
Joystick control unit	CS1521DP	Prior Scientific	https://www.prior.com/product/cs152dp
Monochrome CMOS Camera	Mako G- 040B	Allied Vision	https://www.alliedvision.com/en/camera-selector/detail/mako/g-040/
Power supply for CMOS camera	12 V, 2 A, 8-pin	Allied Vision	https://www.edmundoptics.com/p/allied-vision-12v-2a-8-pin-hirose-power-supply/43032/#
myDAQ University Kit	781326-01	National Instruments	https://www.ni.com/en-us/shop/model/mydaq-university-kit.html
Fluorescence stereomicroscope	M165 FC	Leica	https://www.leica-microsystems.com/products/light-microscopes/stereo-microscopes/p/leica-m165-fc/
Hoya colored glass Long-pass filters	Hoya Y52 (520 nm), 12.5 mm dia., 1 mm thick	Hoya Corporation	https://www.edmundoptics.com/p/hoya-y52-520nm-125mm-dia-1mm-thick-colored-glass-longpass-filter/46497/
Monochrome CMOS Camera	DMK 37BUX273	The Imaging Source	https://www.theimagingsource.com/en-us/product/industrial/37u/dmk37bux273/

This table lists the hardware components, along with their model numbers, manufacturers, and weblinks to the products.

##### Camera: Mako G-040B (Allied Vision)

This black-and-white C-mount camera (Table 1) has a resolution of 728 × 544 pixels and uses GigE for video output. The relatively low resolution of this camera allows for smaller image file sizes and faster image processing. Images captured by this camera can be easily converted to high-quality binary images by the *WormTracker* software, even for those acquired in the presence of a bacterial lawn. This is in sharp contrast to the Sony XCD-V60 used in our original *WormTracker* (Wang and Wang, 2013), which prohibits tracking worms on a bacterial lawn. We strongly recommend using the Mako G-040B camera, though many newer cameras may work equally well. To operate this camera, you will need either a dedicated power supply, such as the Allied Vision 12 V 2 A 8-pin Hirose Power Supply (Table 1), or a power over Ethernet (PoE) single port injector (Table 1). If you choose a different camera, ensure it allows precision time control by MATLAB and can be configured by MATLAB to a resolution of 728 × 544 pixels.

##### External device controller: myDAQ University Kit (National Instruments)

This device (Table 1) is not required for typical worm tracking. *WormTracker* can control up to three external devices using myDAQ via TTL signals.

##### Fluorescence light source

To apply optogenetic stimuli during worm tracking, a fluorescence light source with adequate power and the ability to be controlled by TTL signals is required. Additionally, a long-pass filter with a 520 nm cutoff is necessary in the illuminating light path to prevent blue light from desensitizing GCaMP6. In our setup with a Leica M165 FC fluorescence stereomicroscope, we place a 520 nm long-pass cutoff filter (Y52, Hoya Corporation; Table 1) directly on the circular glass of the microscope base, which transmits light from a halogen bulb.

#### Additional hardware for *SleepTracker*

##### Camera: DMK 37BUX273 (The Imaging Source)

This black-and-white C-mount camera (Table 1) has a resolution of 1,440 × 1,080 pixels and uses USB3.1 for video output. This camera is used instead of the Mako G-040B for tracking worm sleep behavior due to its higher resolution.

##### Sleep recording “chamber”

We use a rectangular polydimethylsiloxane (PDMS) membrane (14.0 mm × 10.5 mm × 0.65 mm) containing six circular openings (3.0 mm in diameter) for recording sleep behavior. This membrane forms six “chambers” when its bottom is in contact with the agarose surface of the nematode culture plate. A CAD design of the sleep chamber is provided in Extended Data Figure 4-1.

### Software installation and hardware configuration

Users need to install the software (standalone or MATLAB-dependent) and appropriate hardware drivers onto their computers. Instructions for software downloading, installation, and hardware configuration are provided in Extended Data 3.

10.1523/ENEURO.0224-25.2025.d3 Extended Data 3 **Installation instructions for the Standalone version and hardware drivers.** This file provides a step-by-step guide for installing and launching the standalone version of the software suite, as well as installing the necessary hardware drivers. It also explains how to verify proper stage operation and how to recenter the stage if needed. Download Extended Data 3, DOCX file.

### *WormTracker* file organization

#### Recording files

*WormTracker* integrates image, stage, time, and spline files for analysis. These files must be organized hierarchically for automatic access. For example, to record 10 wild-type worms (named *wt1* to *wt10*) in a parent folder named *Wild Type*:

1. Create an initial folder structure (*Wild Type\wt1*) in Windows Explorer.
2. In the *Record* module, select *wt1* as the *Image Folder*, and record the first worm's images.
3. For subsequent worms, simply change the number in the path (e.g., from *wt1* to *wt2*) in the *Image Folder* and press Enter to record the next worm's images.

After recording, the *Wild Type* folder will contain 10 subfolders (*wt1*–*wt10*), each with image files (e.g., *img00001.jpeg*, *img00002.jpeg*, etc.), a *Stage File* (e.g., *wt1.txt*), and a *Time File* (e.g., *wt1_times.txt*). If you recorded with ventral and dorsal differentiation, files are prefixed with “*R_*” or “*L_*”.

When reducing frame rate of a recording (e.g., *wt1*) using the *Playback* module, a *Removed* subfolder containing all the removed images is automatically created within the *wt1* subfolder. A new *Time File*—using the same file name as the original—is also generated. The original *Time File* is renamed (e.g., *wt1_times_before_RF.txt*). The *Restore* tab in the *Playback* module can revert all changes generated by frame rate reduction.

#### Spline fitting files

When fitting splines for images in the *wt1* subfolder using the *Fit Spline* or *Batch Spline* module, the resulting spline file (e.g., *wt1_spline.txt*) is automatically saved in the *Wild Type* parent folder, not the *wt1* subfolder. If you have followed the procedures described above, *WormTracker* can (1) automatically identify the existing spline file (if available) when loading a recording in the *Fit Spline* module; (2) automatically identify the existing spline file (if available) when loading a recording in the *Playback module*; and (3) automatically load the *Stage File* and *Time File* when loading a spline file in the *Analyze* and *Batch Analyze* modules.

#### Analysis files

When using the *Analyze* and *Batch Analyze* modules, users are prompted to save results in Excel files. We recommend saving these files in the same parent folder (e.g., *Wild Type*) for easy access.

#### *C. elegans* culture and maintenance

*C. elegans* hermaphrodites were grown on standard nematode growth medium (NGM) plates with a layer of OP50 *Escherichia coli* in the center at 22°C inside an environmental chamber. For *WormTracker* recordings, a young adult worm was transferred to a plate either with or without OP50. For *SleepTracker* recordings, four L4-stage worms were individually placed in the center of each sleep recording chamber, each containing a spot of OP50 in the center.

### Sample recordings

Folders containing sample *WormTracker* and *SleepTracker* recordings are provided as Extended Data 4 and 5, respectively. Two AP recordings, acquired using *pClamp* software (version 11, Molecular Devices), are also provided: one from a mouse suprachiasmatic nucleus neuron (Extended Data 6) and one from a *C. elegans* body-wall muscle cell (Extended Data 7). Users can use these recordings to evaluate the suitability of the software system for their own laboratories.

10.1523/ENEURO.0224-25.2025.d4 Extended Data 4 A sample *WormTracker* recording. This folder contains the recording of a wild-type worm (60 seconds, 15 frames per second), along with the associated stage file, time file, and a spline file generated by the Fit Spline module. The images were captured at 50% of the camera's resolution (4 KB/image). Download Extended Data 4, ZIP file.

10.1523/ENEURO.0224-25.2025.d5 Extended Data 5 A sample *SleepTracker* recording. This folder contains the recording of four wild-type worms (10 hrs., 1 frame/10 sec) along with the associated times file. Due to its large size, this recording is stored on the Zenodo server (https://zenodo.org/records/15857492) for download. Download Extended Data 5, DOCX file.

10.1523/ENEURO.0224-25.2025.d6 Extended Data 6 **A sample trace of action potentials recorded from a mouse suprachiasmatic nucleus neuron.** The action potentials were triggered by current injection steps (-10 to +30 pA at 5-pA intervals, 5 sec/step). This file requires *ClampFit* to open. Download Extended Data 6, ZIP file.

10.1523/ENEURO.0224-25.2025.d7 Extended Data 7 A sample trace of spontaneous action potentials recorded from a *C. elegans* body-wall muscle cell. This file requires *ClampFit* to open. Download Extended Data 7, ZIP file.

## Tracking and Analysis Algorithm

### WormTracker

#### Stage tracking

*WormTracker* adjusts the stage position at 1 s intervals based on worm's movement, with corrections applied between image acquisitions to prevent blurring. A *Stage File* records all stage positions, while a *Time File* logs the timing of each movement.

#### Ventral and dorsal differentiation

Users manually input orientation via the *Record* module before recording begins.

#### Image processing

**Figure 2.**
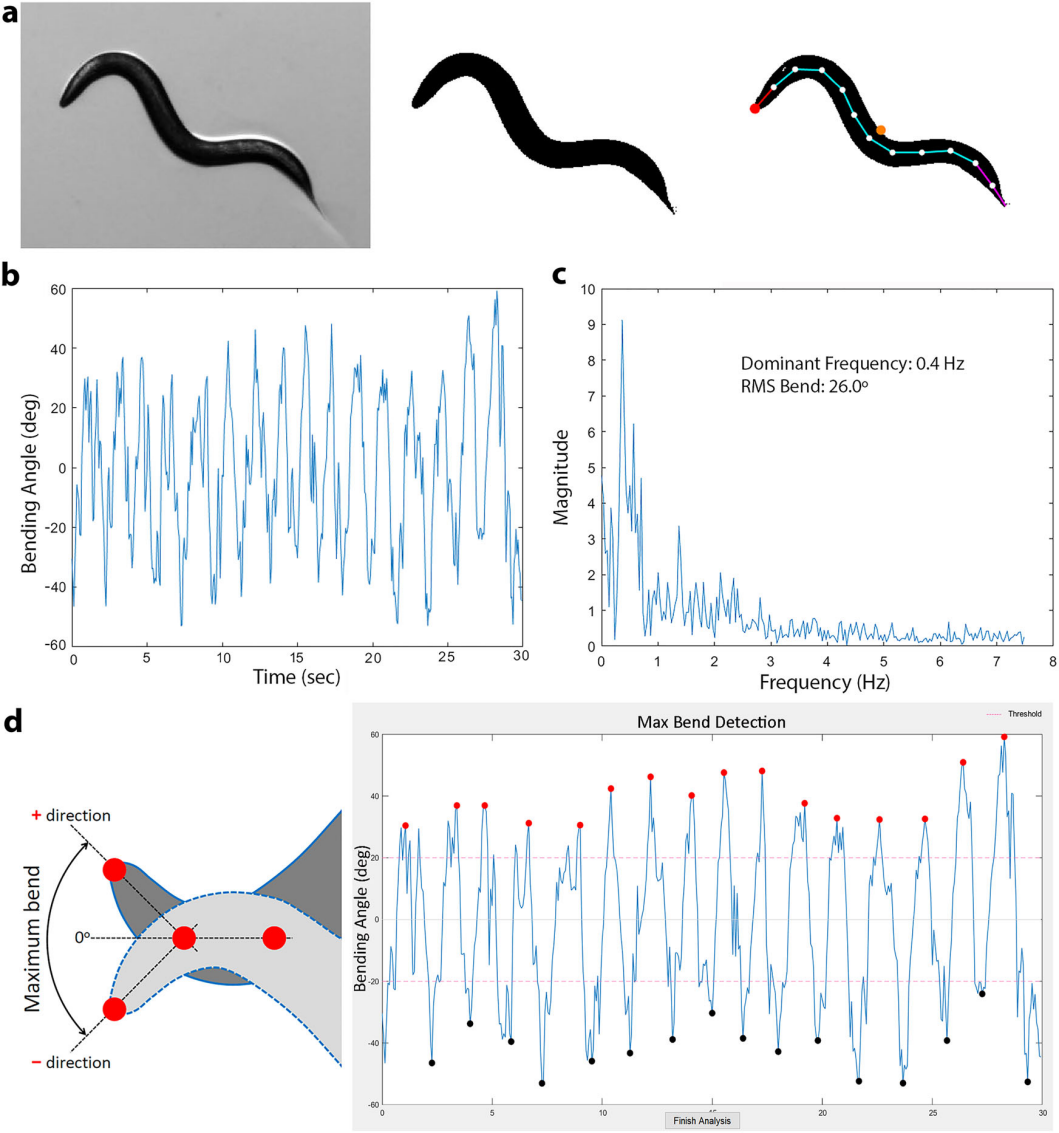
Spline generation and bending property quantification. ***a***, Conversion of a grayscale image (left) to a binary image (middle), followed by spline fitting to the binary image (right). The spline is generated by placing 13 markers at equal intervals along the longitudinal axis of the worm's body, with the marker at the head tip shown in red and the centroid in orange. ***b***, Bend trace of a worm recorded over 30 s. ***c***, Bending frequency spectrum of the same worm, with the dominant bending frequency and RMS (root mean square) bend angle from the Results section displayed within the graph. ***d***, Quantification of the maximum bend. Left, Diagram illustrating the definition of the maximum bend, which is the maximum bending angle of a spline marker point in the ventral and dorsal directions relative to a straight line fitted to the two subsequent spline markers. Right, A bend trace with *x* and *y* coordinates marking the times and bending angles of alternative peaks and troughs. The coordinates are determined by clicking on the peaks and troughs, using a 20° threshold.

Recorded images are processed to produce a spline. The original grayscale images are converted to binary using a user-selected brightness threshold, with pixels above threshold becoming white and darker pixels becoming black. Objects smaller than 3,000 pixels are removed to eliminate debris. Edge detection finds the worm's outline, and the raw edge data are smoothed into *x*–*y* coordinates, transforming the image from grayscale to binary (Fig. 2*a*).

#### Head identification

A corner-detection algorithm identifies the tail (sharpest corner) and head (second-sharpest corner) candidates. The user confirms head orientation for the first frame during spline fitting. For subsequent frames, the software reapplies corner detection while using the previous frame's head position as a spatial reference.

#### Spline generation

A 13-marker midline spline is generated via cubic interpolation (Fig. 2*a*). The centroid is the average marker position. Failed fits (due to poor thresholds, image quality, or Ω shapes) trigger up to 10 automatic threshold adjustments. Full-resolution images are temporarily downsampled to 50% during fitting (stored in “resized_temp”).

#### Bending property quantification

*WormTracker* plots bending activity as a Bend Trace (Fig. 2*b*), which serves as the basis for detailed quantifications. Bending at each spline marker (1–11) is quantified relative to a straight line connecting the two subsequent markers. The dominant bend frequency is identified via Fourier transform of the bend trace (Fig. 2*c*), with the most prominent peak defined as the dominant frequency.

*Maximum Bend* is calculated as the difference between averages of ventral and dorsal dominant bends (Fig. 2*d*). Its utility is demonstrated by the significant increase in Maximum Bend in *slo-1* mutants (encoding the large-conductance Ca^2+^- and voltage-activated potassium channel) versus wild type (Kim et al., 2009; Chen et al., 2010).

RMS Bend is calculated as follows:

$$\mathrm{RMS}=\;\sqrt{\frac{\sum_{i}^{n}\;x_{i}^{2}}{n}},$$

where *n* is the total number of data points and *i* refers to the index of each individual point. While RMS Bend values can be positive or negative, *Analysis* module displays the average absolute values. *Sum of All Bends* provides the total of all 11 RMS bends.

#### Speed, distance, and direction quantification

These metrics can be calculated using the positions of any of the 13 markers along the spline with differing results. The calculation of these movement metrics is based on the actual frames if the frame rates are 1, 3, or 5 per second, but defaults to 3 frames per second (FPS) if the recording was acquired at 15 FPS.

**Figure 3.**
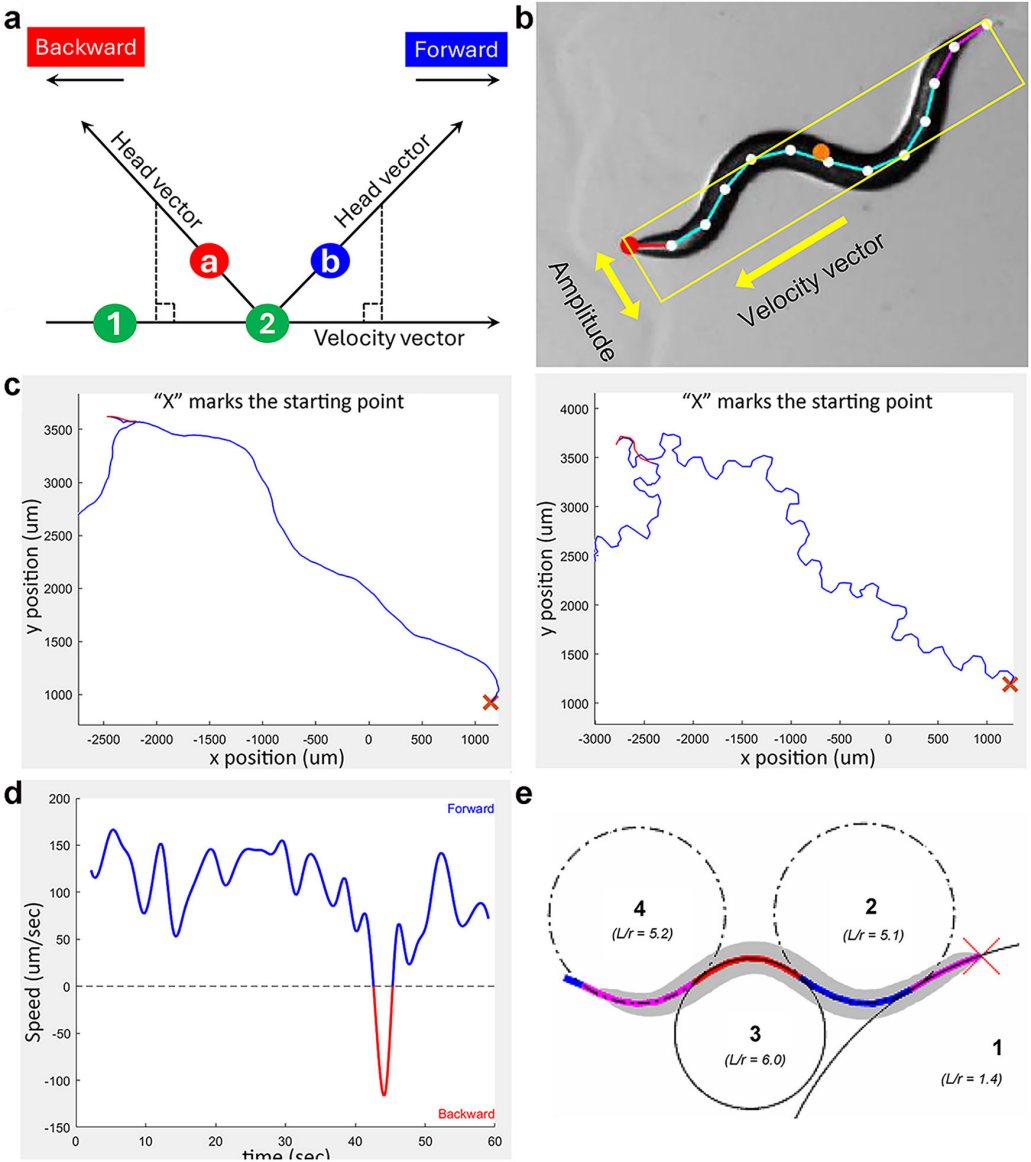
Analysis of locomotion directionality, speed, and body curvatures. ***a***, Directionality is determined by examining the relationship between the velocity vector and the head vector. The velocity vector connects the centroids of the current (#2) and previous (#1) frames, while the head vector connects the current centroid to the current head point (***a*** or ***b***). If the head vector projects onto the positive side of the velocity vector, the worm is moving forward; otherwise, it is moving backward. ***b***, Method for quantifying worm amplitude. A rectangle is drawn parallel to the velocity vector, just large enough to enclose all spline markers. The width of the rectangle is the worm's amplitude. ***c***, Reconstructed worm travel paths based on the positions of the centroid (left) and spline marker 1 (right). ***d***, Plots of forward and backward locomotion speeds over time shown for interpolated data. ***e***, Method for quantifying body curvatures. Circles are fitted to worm segments, with curvature values calculated as the worm length (*L*) divided by the radius *r* of each fitted circle (*L*/*r*).

*WormTracker* measures the average speed, total distance traveled, and net distance traveled (the straight-line distance between the first and last positions). Directionality is determined by comparing the velocity vector (last to current centroid) with the head vector (current centroid to current head tip). If the head vector's projection onto the velocity vector is positive, the worm moves forward; otherwise, it moves backward (Fig. 3*a*).

#### Length and amplitude

Length (*L*) is calculated as the straightened spline. Amplitude (*A*) is measured by aligning a rectangular box with the worm's velocity vector and adjusting its width to fit all spline points (Fig. 3*b*). The *A*/*L* ratio normalizes amplitude to account for differences in worm size.

#### Travel path reconstruction

Travel path can be based on the positions of the centroid or any of the 13 spline markers (Fig. 3*c*). It is plotted according to the actual frame rate for recordings at 1, 3, or 5 FPS, but 3 FPS for recordings acquired at 15 FPS.

*WormTracker* measures forward/backward travel distances and speeds, plotting speed over time using binned or unbinned data (Fig. 3*d*).

#### Body curvature quantification

The *Curve Analyzer* smoothes the spline (13 markers) and detects inflection points, excluding short segments (<3% length) to reduce noise. Using the Kasa method (least-squares circle fitting), it calculates curvature (*L*/*r*) from the radius (*r*) of best-fit circles for each segment (Fig. 3*e*). Segment midpoints are derived from start/end position averages.

### SleepTracker

**Figure 4.**
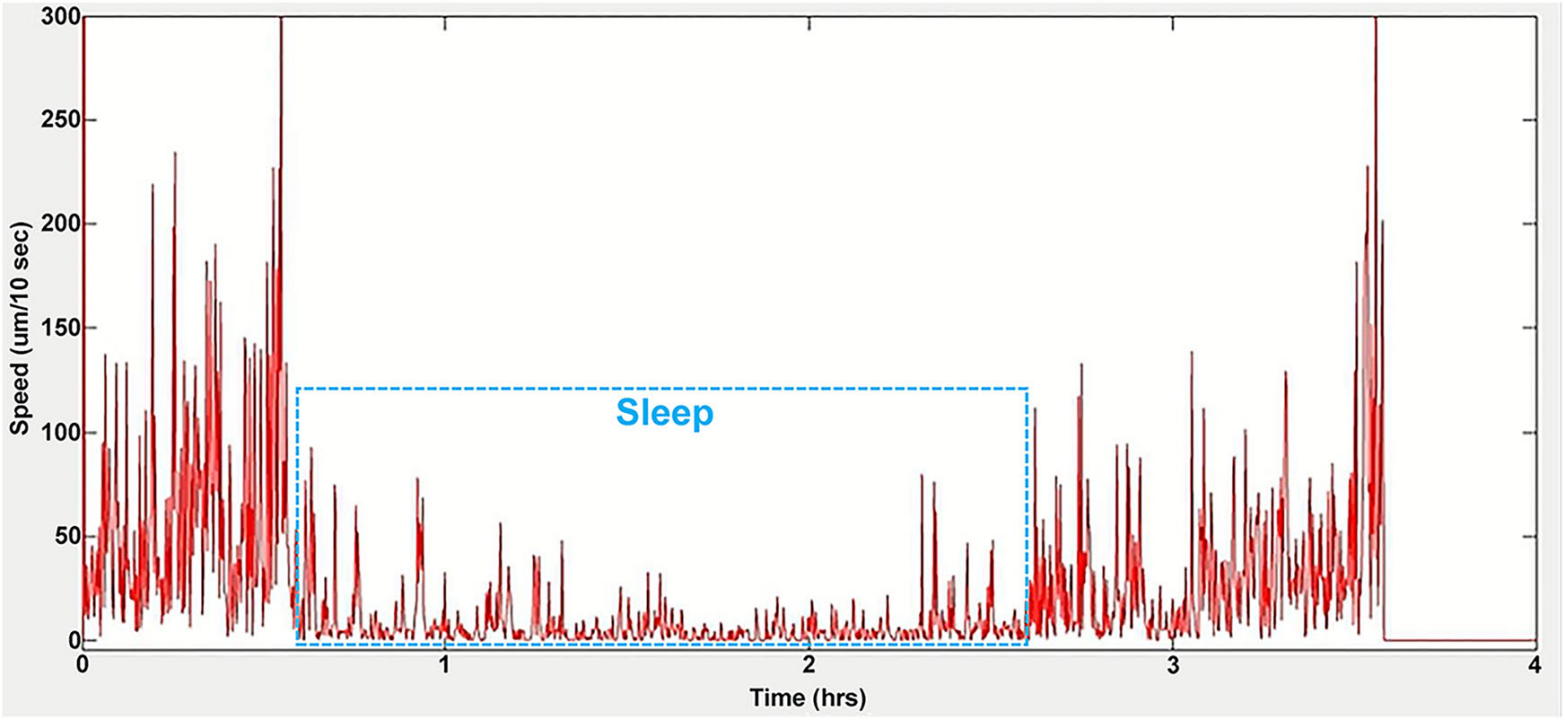
Quantification of sleep duration in freely moving worms using *SleepTracker*. The actogram shows the locomotor activity of a worm developing from the L4 to young adult stages. Images were captured at a rate of 1 frame every 10 s. Sleep duration, highlighted by the rectangular box, is defined as the period from the start of three consecutive motionless frames to the end of the last three motionless frames. The worm is considered motionless if the difference between the centroid positions of two consecutive frames is <10 µm. Spikes within the sleep period are classified as active events. Extended Data Figure 4-1 illustrates the design of the PDMS chamber.

*SleepTracker* identifies sleep-like behavior by measuring worm locomotor activity through centroid displacement between consecutive frames (typically acquired at 1 frame/10 s). A motionless state is defined as <10 µm displacement between frames, while >10 µm indicates activity. Sleep onset begins with three consecutive motionless frames, and sleep ends when the last of three consecutive motionless frames concludes; total sleep duration spans these points (Fig. 4). Third-party software (detailed later) further analyzes active events during sleep and calculates motionless sleep duration by subtracting active periods from total sleep time.

10.1523/ENEURO.0224-25.2025.f4-1 Figure 4-1 **CAD design of the polydimethylsiloxane (PDMS) sleep recording chamber.** This schematic illustrates the custom-fabricated PDMS membrane used to isolate individual C. elegans during sleep behavior recordings. The membrane features six uniformly spaced circular wells (3.0  mm diameter) embedded in a rectangular frame (14.0  mm × 10.5  mm × 0.65  mm). Download Figure 4-1, TIF file.

### AP Analyzer

**Figure 5.**
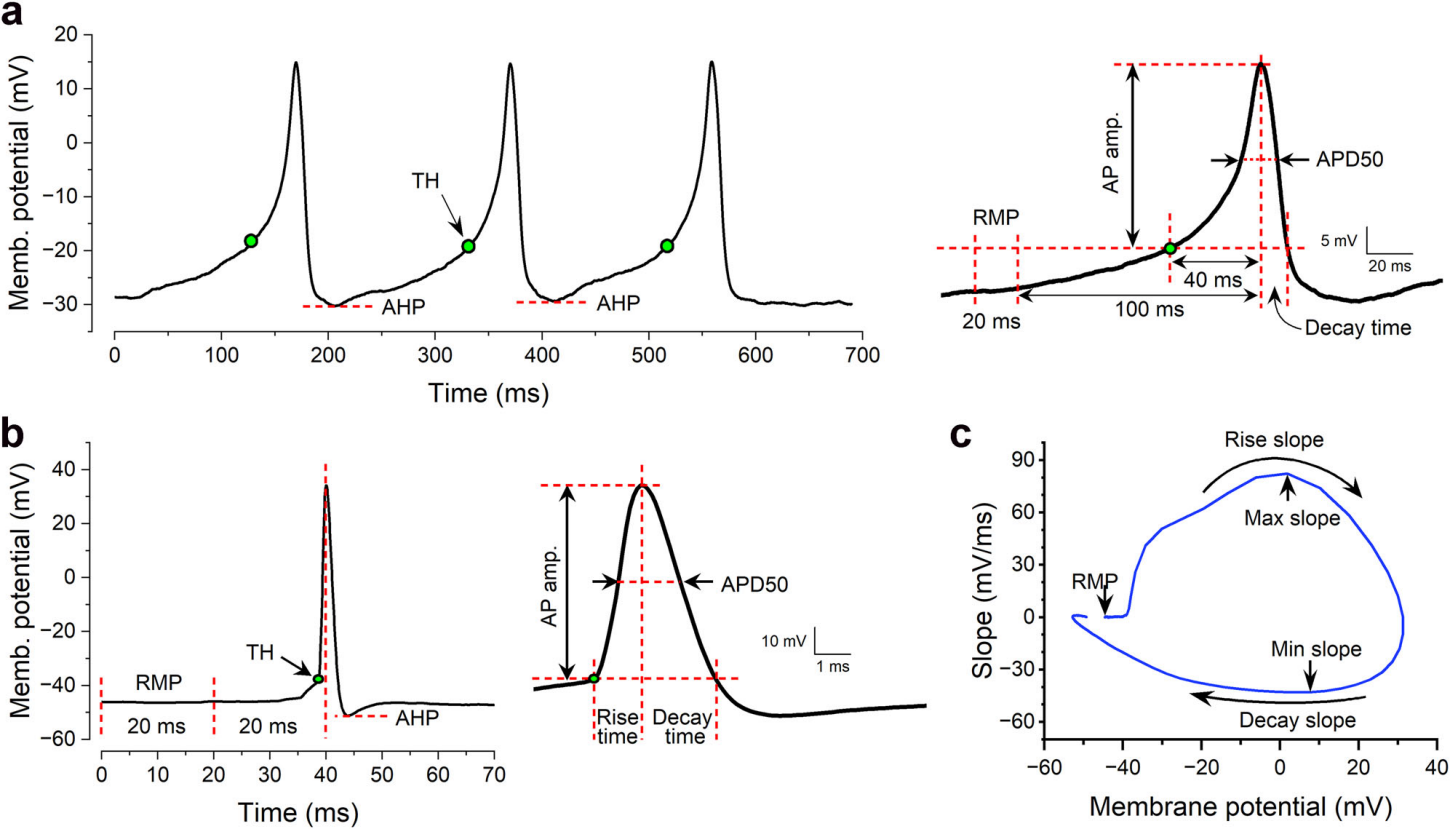
Quantification of action potential (AP) properties using *AP Analyzer* with two different approaches. ***a***, AP threshold is the membrane potential at a user-defined pre-AP peak time for APs lacking a preupstroke inflection point. ***b***, AP threshold is automatically detected for APs with a preupstroke inflection point. The representative APs are from a body-wall muscle cell of a wild-type *C. elegans* (***a***) and a suprachiasmatic nucleus (SCN) neuron of a wild-type CBA/CaJ mouse (***b***). The RMP is defined as the 20 ms average preceding the user-selected time point for non-inflected APs, and as the lowest 3 ms average within that 20 ms window for inflected APs. The rise time is defined as the time from AP threshold to AP peak, the decay time from AP peak back to the membrane potential matching the AP threshold, and afterhyperpolarization (AHP) as the actual membrane voltage. ***c***, Voltage phase plot of an SCN neuron AP from a mouse, illustrating the quantification of AP maximum and minimum slopes, corresponding to the points where the membrane potential increases and decreases most rapidly, respectively.

Nearly all *C. elegans* electrophysiology experiments use the *pClamp* acquisition system. AP Analyzer addresses a key limitation in *ClampFit* by providing automated AP threshold detection through two distinct methods. For APs lacking an inflection point before the upstroke (e.g., *C. elegans* body-wall muscle; Fig. 5*a*), the threshold is defined as the membrane potential at a fixed, user-defined time before the AP peak, enabling standardized parameter measurement across recordings. APs with clear inflection points (Fig. 5*b*) have automatically detected thresholds.

The software measures resting membrane potential (RMP), AP threshold, amplitude, APD50 (AP duration at 50% amplitude), AHP level, rise time (threshold-to-peak), decay time (peak-to-threshold), and maximum/minimum slopes from phase plots (Fig. 5*c*). RMP calculation depends on AP category: for noninflected APs, it is the 20 ms average preceding the user-selected time point; for inflected APs, it is the lowest 3 ms average within that window. When frequent APs prevent complete repolarization, RMP should be manually measured from a quiescent segment of the recording trace using *ClampFit*.

*Track-A-Worm 2.0* includes multiple user software interfaces. The use of these interfaces is demonstrated in the video tutorials (Movies 1–11).

## Discussion

We have developed a comprehensive software and hardware system designed to quantify worm locomotor behavior and bending properties, identify sleep episodes and quantify their durations in freely moving worms, and determine AP properties in *C. elegans*. To our knowledge, this is the only open-source system that combines these functionalities.

*WormTracker* is both powerful and user friendly, capable of quantifying all locomotor and bending metrics typically needed by *C. elegans* researchers. The system allows the user to incorporate the worm's ventral and dorsal orientation information into recordings. It can track a single worm over extended periods by adjusting stage positions between frames and integrate external devices via TTL signals.

One limitation of *WormTracker* is that images of coiled worms sometimes cannot be automatically fitted to the spline, requiring user-assisted semi-automatic methods. However, this generally does not present a significant problem, as typical recordings for quantifying locomotion and body bending metrics last only 30 s to a few minutes.

**Figure 6.**
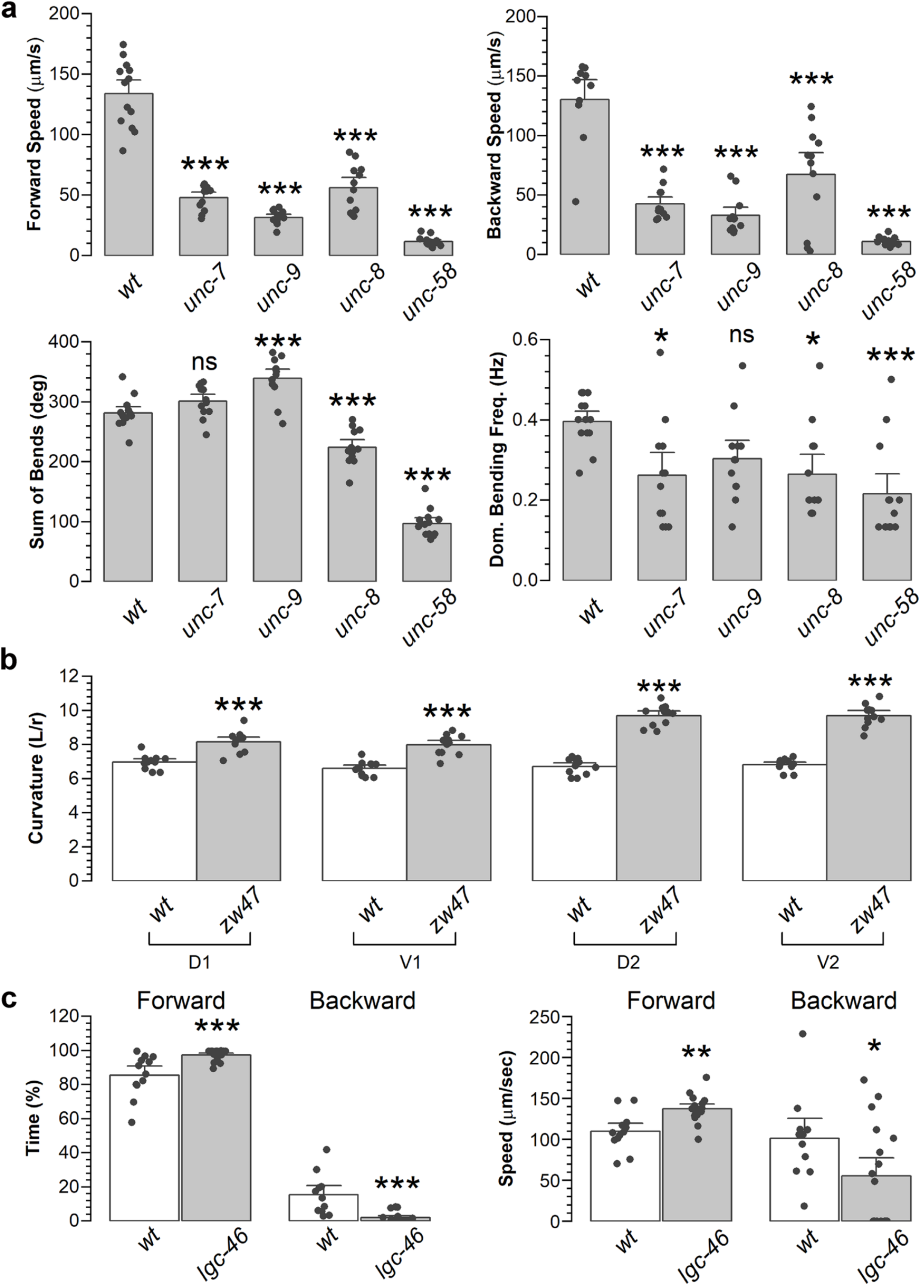
Comparison of locomotion and body bending properties between wild-type and mutant worm strains demonstrating *WormTracker* functionality. ***a***, *unc-7(e5)*, *unc-9(fc16)*, *unc-8(e1069)*, and *unc-58(n495)* mutants showed reduced locomotion speed and abnormal body bending properties compared with wild type (*wt*). The number of worms analyzed was 13 in each group. ***b***, The *zw47* mutant exhibited enhanced body curvatures. Curvatures were measured in the head-to-tail direction, with D1 and D2 representing the first and second dorsal curvatures and V1 and V2 representing the first and second ventral curvatures. If the original V1 or D1 curvature value was less than 2.5, it was excluded from the analysis, and the subsequent V2 or D2 was reassigned as V1 or D1, with V3 or D3 becoming V2 or D2. The number of worms analyzed was 11 in each group. ***c***, The *lgc-46(ok2949)* mutant exhibited enhanced forward locomotion but reduced backward locomotion compared with *wt*. The number of worms analyzed was 12 *wt* and 17 *lgc-46*. All recordings (60 s each, 15 frames/s) were conducted with NGM plates without OP50. Following the transfer of each worm, a 30–60 s interval was allowed before initiating the recording. The single, double, and triple asterisks indicate statistically significant differences compared with *wt* at *p* < 0.05, *p* < 0.01, and *p* < 0.001, respectively, while ns indicates no significant difference compared with *wt* based on one-way *ANOVA* with Tukey's post hoc test (***a***) or un-paired *t*-test (***b***, ***c***).

To illustrate the usefulness of *WormTracker*, we compared locomotor and bending properties between wild type and mutants of several genes, including *unc-7* (innexin), *unc-9* (innexin), *unc-8* (degenerin/epithelial sodium channel), *unc-58* (two-pore domain K^+^ channel), *lgc-46* (an acetylcholine receptor), and *zw47* (a hypomorphic mutant of a calcium-binding protein isolated in one of our genetic screens, unpublished). All the *unc* mutants exhibited significant differences in bending angles, dominant bending frequency, and forward and backward speeds compared with wild type (Fig. 6*a*). The *zw47* mutant showed a significant increase in body curvatures compared with wild type (Fig. 6*b*), while the *lgc-46* mutant spent more time in forward locomotion but less time in backward locomotion and moved faster in the forward direction but slower in the backward direction compared with wild type (Fig. 6*c*).

**Movie 1.** *Calibrate* module of *WormTracker*. This module is used to determine the pixel-to-micrometer conversion factor for quantifying locomotion and bending properties. The resulting conversion factor must be entered into the *Record* module before starting the recording. [View online]

**Movie 2.** *Record* module of *WormTracker*. This module captures worm images, recenters the worm position at 1 s intervals, saves the images, and records the positions and timing of stage movements as the Stage File and Time File, respectively. It allows the user to enter ventral/dorsal orientation information prior to the recording and can control up to three external devices in customizable on/off patterns via TTL signals. [View online]

**Movie 3.** *Playback* module of *WormTracker*. This module allows users to view recorded sequential images as a movie, adjust the frame rate (by reducing or restoring it), and create movies from the recordings. Images can be displayed in grayscale or binary format, with or without the fitted spline. If spline fitting has been performed, the corresponding spline file is automatically detected and can be activated by clicking the *Load* tab next to it. Movies can be exported in grayscale or binary, with or without the spline overlay. This module is particularly useful for initially assessing the spline-fitting threshold, quickly verifying fitting results, reducing frame rates for faster analysis, and creating movies for publications or presentations. [View online]

**Movie 4.** *Fit Spline* module of *WormTracker*. This module performs two functions: (1) automatic head identification and spline fitting and (2) user-assisted verification and correction of the automatically generated spline. The second function can be applied to splines generated by either the *Fit Spline* or *Batch Spline* module. [View online]

**Movie 5.** *Batch Spline* module of *WormTracker*. This module performs automatic spline fitting across multiple recordings, saving users the time required for processing each file individually. As with the *Fit Spline* module, the results require user-assisted verification and correction using the *Fit Spline* module. [View online]

**Movie 6.** *Analyze* module of *WormTracker*. This module quantifies worm bending and locomotion metrics based on the *Spline File* and the corresponding *Stage File* and *Times File*. The results are saved in user-defined Excel files. [View online]

**Movie 7.** *Batch Analyze* module of *WormTracker*. This module allows users to perform automated analysis across multiple recordings. [View online]

**Movie 8.** *Curve Analyzer* module of *WormTracker*. This module calculates curvature values by fitting the spline with circles, which are displayed as solid lines (ventral), dash-dot lines (dorsal), or dotted lines (undifferentiated ventral/dorsal). Users can export the curvature values to an Excel file that includes position data (normalized by body length), ventral/dorsal orientation information (if available), and sequential frame numbers. [View online]

**Movie 9.** *Sleep Recorder* module of *SleepTracker*. This module records worm locomotion activity for detecting sleep-like states. Users can specify the exposure time, interframe interval, and total recording duration. [View online]

**Movie 10.** *Sleep Analyzer* module of *SleepTracker*. This module quantifies worm locomotor activity over time, with the exported data used to calculate total sleep duration. The number and duration of active events during sleep can also be quantified using the Quick Peaks gadget in OriginPro (OriginLab), based on a threshold of ≥10 μm change in centroid position between consecutive images. The duration of motionless sleep is obtained by subtracting the total duration of active events from the total sleep duration. [View online]

**Movie 11.** *AP Analyzer*. This module quantifies action potential (AP) metrics, including threshold, amplitude, APD50 (AP duration at 50% amplitude), rise and decay times, maximum and minimum slopes, and rise and decay slopes. It also measures afterhyperpolarization (AHP) level and resting membrane potential (RMP) and generates an averaged AP trace along with an AP voltage phase plot. The results can be exported to an Excel file, which includes the data used to create both the averaged AP trace and the phase plot. [View online]

*SleepTracker* offers an advantage over methods that confine worms within microfluidic devices by quantifying sleep behavior quickly and accurately in freely moving worms. Recordings must be discarded if the worm had contacted the edge of the recording chamber, as this complicates automatic binary image generation. However, this is generally not a major issue, as most animals (70–80%) remain near the center of the chamber due to the restriction of food source (OP50 bacteria) to the center.

*AP Analyzer* is designed to work with current-clamp data acquired at a 10 kHz sampling rate, which is sufficient for accurately capturing AP waveforms. For APs with a clear preupstroke inflection point, the software automatically detects the AP threshold, enabling accurate quantification of AP metrics. This method is more efficient and objective than the user-assisted manual threshold selection method used in *ClampFit*, which does not account for the effects of baseline fluctuations on measured AP thresholds. While *AP Analyzer* offers an advantage over the widely used *ClampFit* in AP threshold detection, its automatic detection method is not applicable to typical APs in *C. elegans* body-wall muscle cells due to the absence of a preupstroke inflection point. In these cases, our alternative approach of defining the threshold from a specific time before the AP peak ensures consistency across APs, though it remains subjective.

*AP Analyzer* also measures the RMP. However, the measured RMP can differ significantly from the true RMP when interspike intervals are short. We recommend that users, whenever possible, measure the RMP from a segment of the recording without APs to obtain a more accurate measurement.

We hope that many *C. elegans* research laboratories will find our system to be a valuable tool for their work. As an open-source system, users can contribute to improving its functionality by providing feedback and refining software codes.

## Synthesis

Reviewing Editor: Arianna Maffei, Stony Brook University Decisions are customarily a result of the Reviewing Editor and the peer reviewers coming together and discussing their recommendations until a consensus is reached. When revisions are invited, a fact-based synthesis statement explaining their decision and outlining what is needed to prepare a revision will be listed below. The following reviewer(s) agreed to reveal their identity: Linda Dansereau.

The reviewers agree that the software for analysis included in the manuscript is an important contribution to the field. The code was downloaded and tested and appears to work well aside from extended data 4. Addressing this issue would make the code more valuable.

In addition, a few clarifications about sampling and experimental conditions would greatly improve the manuscript.

Specific reviewers concerns are listed below.

Reviewer #1

Because extended data include instructions and codes, they are essential. The code was properly downloaded and tested. One issue was extended data 4, which contains sample recording. This recording file does not work with both standalone and MATLAB-dependent codes (incorrectly says 1800 image, while contains 900 images). This seems specific to the file, as the other one downloaded from the source in the lab worked properly.

Software Comments:

Testing was somewhat limited. This is because a proper test will also require hardware. However, it works fine with downloaded image files (from the source, but not extended data 4).

Statistics:

The one figure that includes analysis of a data-set is missing the N values, or information on how the data was collected to confirm if the analysis was performed correctly.

This manuscript describes a software suite that allows for C. elegans behavior and action potential analysis. While a commercial version is available, it is limited to tracking analysis. This software suite will be very useful to many C. elegans labs and electrophysiology labs. The overall instruction for installation and use is clearly described. The tracker module (analysis, spline, playback, and fit spline) works well. One suggestion- unrelated to the publication recommendation- is that the analysis module's ability to process images from cameras with different pixel sizes and resolutions without changing configuration in MATLAB is truly helpful to the researchers in the field.

Reviewer #2

The manuscript describes a software package that combines 3 forms of behavioural analysis for C. elegans: movement, sleep, and electrophysiology. While other types of these analysis programs exist in separate programs, each module in this system includes additional features that improves the functionality of the software. In addition, the software is open source which makes it more accessible to the broader research community.

The software is easy to install and use. The current functionality as described in the videos will be very useful to researchers, particularly the sleep tracking module for freely moving animals. In addition, the AP analysis module can be used for mammalian neurons as well as C. elegans neurons which will be advantageous for those labs that use both models.

Major comments:

I am curious how easy it is to switch from the movement tracking camera to the sleep tracking camera. Does it involve removing the previous camera, or can both be connected to the microscope at the same time with the right hardware? Can the recommended sleep tracking camera be used for movement tracking?

There are no n-values for the data that is shown in extended data fig 3-2. Also, there is no information on how the animals were maintained.

It would be useful to include sample data for the sleep tracking and AP analysis modules to be used for trial use as well.

## Author Response

Responses to Reviewers' Comments Dear Editor, Thank you for overseeing the review of our manuscript. We sincerely appreciate the reviewers' careful evaluation and their thoughtful comments and suggestions. We have carefully revised the manuscript to address all comments.

Following the instructions from the eNeuro editorial office, we have made the following specific changes:

1. The word count in the "Results" section (Tracking and Analysis Algorithms) has been reduced to fewer than 1,000 words.

2. The "WormTracker File Organization" section has been moved from the "Results" to the Methods section and edited for conciseness.

3. Descriptions of the software interfaces have been removed from the "Results" section and incorporated into the new legends for the Extended Data Movies.

4. Descriptions of selected mutant phenotypes are now included in the Discussion section, and the original Extended Data Figure 3-2 has been renamed Figure 6.

5. Still images corresponding to Movies 1-11 have been added as new figures.

The revised submission includes two versions of the manuscript in Word format for double-blinded review, one with tracked changes and one with changes accepted. Below, we provide our point-by-point responses to the reviewers' comments.

Reviewer #1 Comment: Because extended data include instructions and codes, they are essential. The code was properly downloaded and tested. One issue was extended data 4, which contains sample recording. This recording file does not work with both standalone and MATLAB-dependent codes (incorrectly says 1800 image, while contains 900 images). This seems specific to the file, as the other one downloaded from the source in the lab worked properly.

Response: We thank the reviewer for pointing out this issue. The image folder in Extended Data 4 has been updated and tested to ensure that the recording can now be properly downloaded and analyzed with both the standalone and MATLAB-dependent versions of the software.

Software Comments: Testing was somewhat limited. This is because a proper test will also require hardware. However, it works fine with downloaded image files (from the source, but not extended data 4).

Response: We are glad to hear that the reviewer was able to successfully test our software using the downloaded image files.

Statistics: The one figure that includes analysis of a data-set is missing the N values, or information on how the data was collected to confirm if the analysis was performed correctly.

Response: We thank the reviewer for pointing out these oversights. The revised figure legend of Extended Data Figure 3-2 now contains the following updates: 1) sample size (n) for all groups, and 2) methods used in data collection: "All recordings (60 s each, 15 frames/s) were conducted with NGM plates without OP50. Following the transfer of each worm, a 30-60 interval was allowed before initiating the recording." Comment: This manuscript describes a software suite that allows for C. elegans behavior and action potential analysis. While a commercial version is available, it is limited to tracking analysis. This software suite will be very useful to many C. elegans labs and electrophysiology labs. The overall instruction for installation and use is clearly described. The tracker module (analysis, spline, playback, and fit spline) works well. One suggestion- unrelated to the publication recommendation- is that the analysis module's ability to process images from cameras with different pixel sizes and resolutions without changing configuration in MATLAB is truly helpful to the researchers in the field.

Response: We thank the reviewer for highlighting the strengths of our software system. We agree that enabling the analysis module to automatically process image files from cameras with different resolutions and pixel sizes-without requiring manual configuration in MATLAB-would be highly beneficial to the field. However, our analysis module depends on the use spline files in combination with the corresponding time and stage files. As a result, it is difficult to implement this feature in a generalized way.

Reviewer #2 Comment: The manuscript describes a software package that combines 3 forms of behavioral analysis for C. elegans: movement, sleep, and electrophysiology. While other types of these analysis programs exist in separate programs, each module in this system includes additional features that improves the functionality of the software. In addition, the software is open source which makes it more accessible to the broader research community. The software is easy to install and use. The current functionality as described in the videos will be very useful to researchers, particularly the sleep tracking module for freely moving animals. In addition, the AP analysis module can be used for mammalian neurons as well as C. elegans neurons which will be advantageous for those labs that use both models.

Response: We thank the reviewer for these thoughtful comments.

Major comments:

Comment: I am curious how easy it is to switch from the movement tracking camera to the sleep tracking camera. Does it involve removing the previous camera, or can both be connected to the microscope at the same time with the right hardware? Can the recommended sleep tracking camera be used for movement tracking? Response: For stereomicroscopes commonly used in C. elegans research labs, there is typically only one camera port. As a result, the cameras used for worm tracking and sleep tracking must be physically swapped as needed. Two different cameras are used because these applications require very different resolutions: worm tracking benefits from a low-resolution camera that enables fast image processing, while sleep tracking requires a high-resolution camera to capture finer detail.

Although the sleep tracking camera can be configured to match the resolution of the worm tracking camera, the binary images often fail to clearly distinguish the worm from the background, particularly when OP50 is present or the microscope base illumination is not properly adjusted. This suggests that factors beyond resolution, possibly related to sensor characteristics or image processing compatibility, influence a camera's suitability for worm tracking. For instance, the Sony XCD-V60 used in our original worm tracking system has a resolution similar to our current camera, yet it could not track worms reliably on NGM plates with OP50.

Comment: There are no n-values for the data that is shown in extended data fig 3-2. Also, there is no information on how the animals were maintained.

Response: We thank the reviewer for pointing out these oversights. In the revised manuscript, the sample sizes (n values) have been added to the legend of Extended Data Figure 3-2. In addition, details on how C. elegans were maintained are now included in the Materials and Methods section (pages 8-9), as shown below: "C. elegans culture and maintenance C. elegans hermaphrodites were grown on standard nematode growth medium (NGM) plates with a layer of OP50 Escherichia coli in the center at 22{degree sign}C inside an environmental chamber. For WormTracker recordings, a young adult worm was transferred to a plate either with or without OP50. For SleepTracker recordings, four L4-stage worms were individually placed in the center of each sleep recording chamber, each containing a spot of OP50 in the center." Comment: It would be useful to include sample data for the sleep tracking and AP analysis modules to be used for trial use as well.

Response: We thank the reviewer for this suggestion. The revised manuscript now has sample data for SleepTracker (Extended Data 5) and AP Analyzer (Extended Data 6 &7).
