# Supplementary figures and images for: Track-A-Worm 2.0: A Software Suite for Quantifying Properties of C. elegans Locomotion, Bending, Sleep, and Action Potentials (part 1 of 3)
Source: eNeuro. 2025 Aug 13;12(8):ENEURO.0224-25.2025. doi: 10.1523/ENEURO.0224-25.2025 (PMC12393025; doi:10.1523/ENEURO.0224-25.2025)

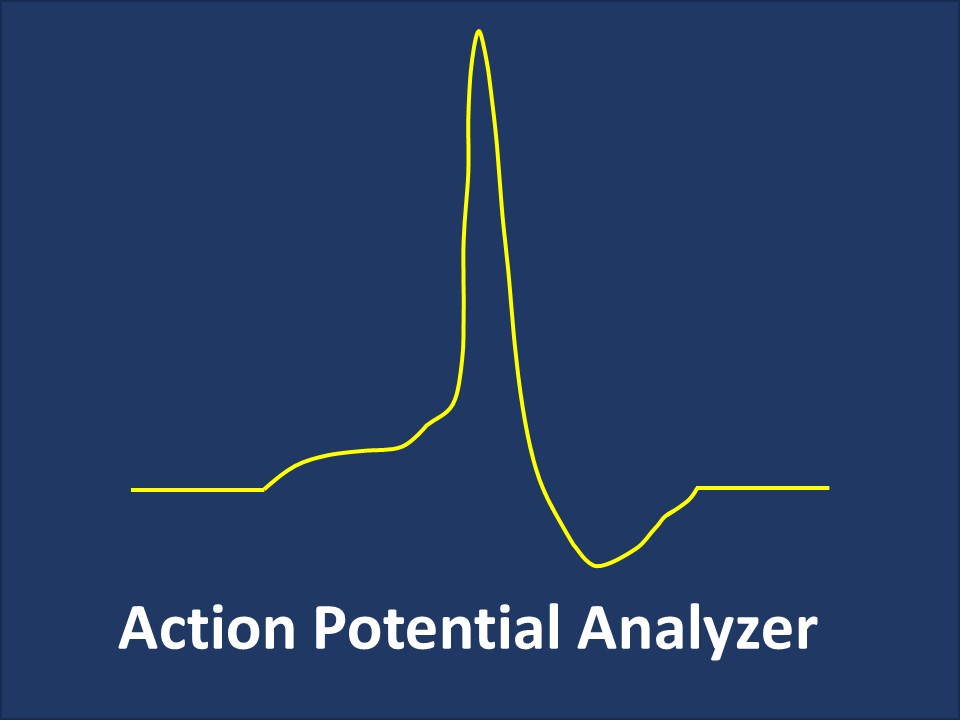

Supplement: Extended Data 2 — MATLAB codes for the software suite. This folder contains the MATLAB codes of the software. Users can run the software and configure none “standard” hardware with MATLAB (R2021a or later) installed on their computers. Download Extended Data 2, ZIP file. [file eneuro-12-ENEURO.0224-25.2025-s004.zip › Extended Data 2/AP_Analyzer.jpg]

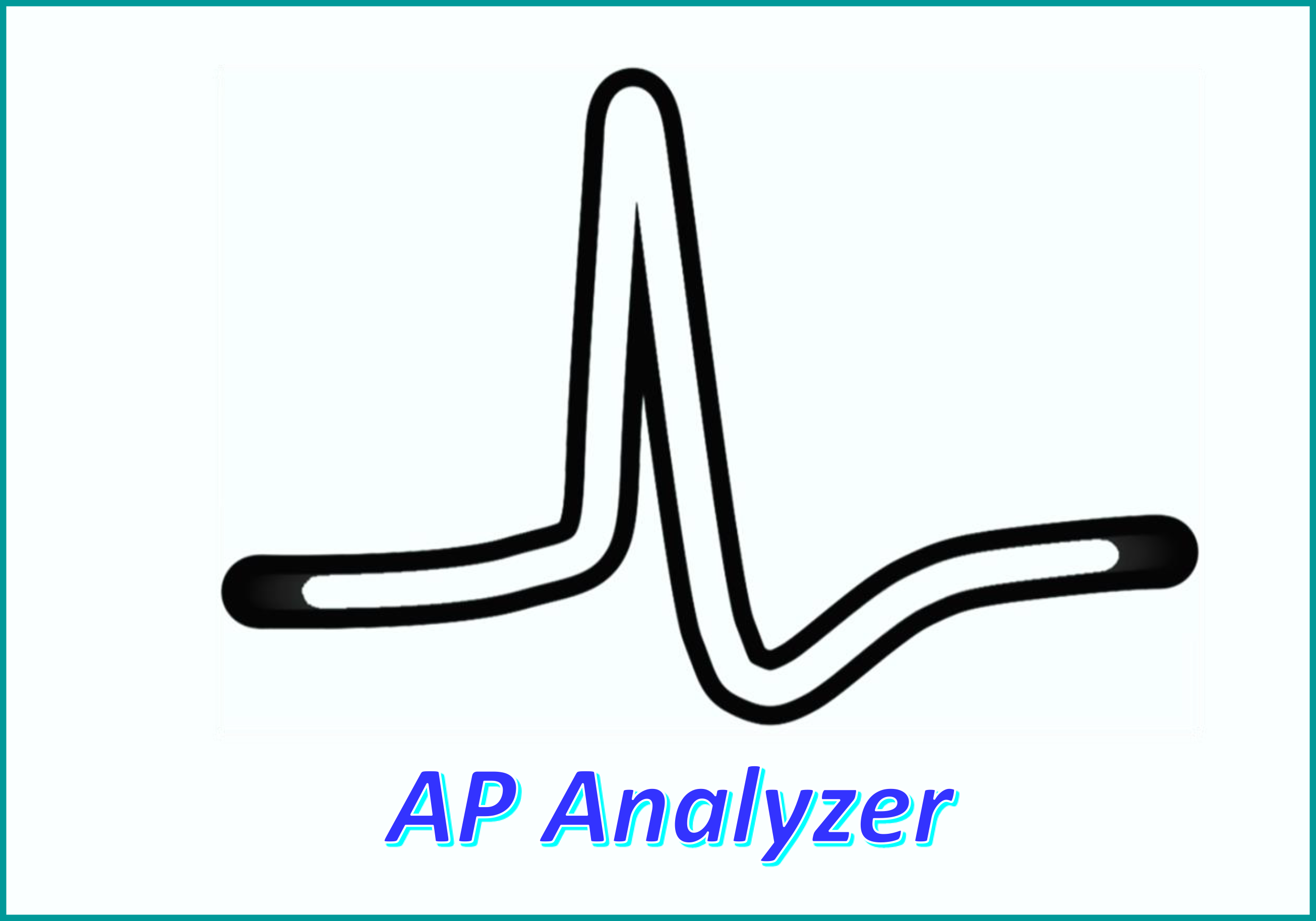

Supplement: Extended Data 2 — MATLAB codes for the software suite. This folder contains the MATLAB codes of the software. Users can run the software and configure none “standard” hardware with MATLAB (R2021a or later) installed on their computers. Download Extended Data 2, ZIP file. [file eneuro-12-ENEURO.0224-25.2025-s004.zip › Extended Data 2/APAnalyzer_logo.tif]

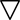

Supplement: Extended Data 2 — MATLAB codes for the software suite. This folder contains the MATLAB codes of the software. Users can run the software and configure none “standard” hardware with MATLAB (R2021a or later) installed on their computers. Download Extended Data 2, ZIP file. [file eneuro-12-ENEURO.0224-25.2025-s004.zip › Extended Data 2/backup/down-button.png]

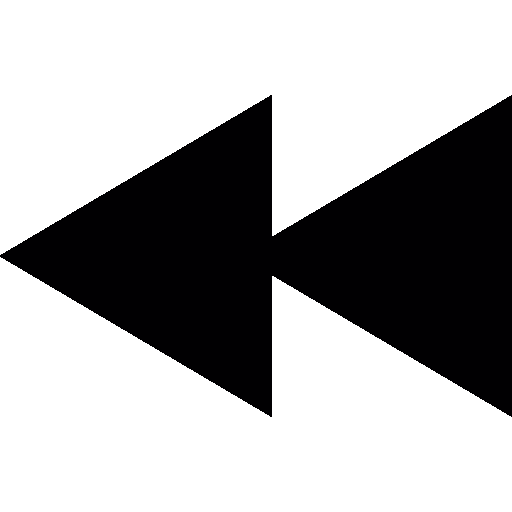

Supplement: Extended Data 2 — MATLAB codes for the software suite. This folder contains the MATLAB codes of the software. Users can run the software and configure none “standard” hardware with MATLAB (R2021a or later) installed on their computers. Download Extended Data 2, ZIP file. [file eneuro-12-ENEURO.0224-25.2025-s004.zip › Extended Data 2/backup/fast-backward-button.png]

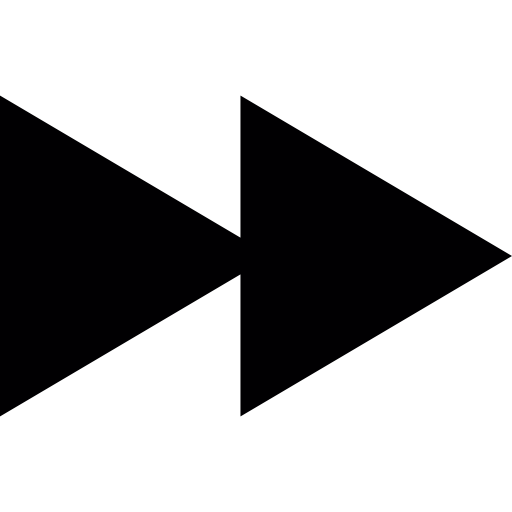

Supplement: Extended Data 2 — MATLAB codes for the software suite. This folder contains the MATLAB codes of the software. Users can run the software and configure none “standard” hardware with MATLAB (R2021a or later) installed on their computers. Download Extended Data 2, ZIP file. [file eneuro-12-ENEURO.0224-25.2025-s004.zip › Extended Data 2/backup/fast-forward-button.png]

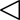

Supplement: Extended Data 2 — MATLAB codes for the software suite. This folder contains the MATLAB codes of the software. Users can run the software and configure none “standard” hardware with MATLAB (R2021a or later) installed on their computers. Download Extended Data 2, ZIP file. [file eneuro-12-ENEURO.0224-25.2025-s004.zip › Extended Data 2/backup/left-button.png]

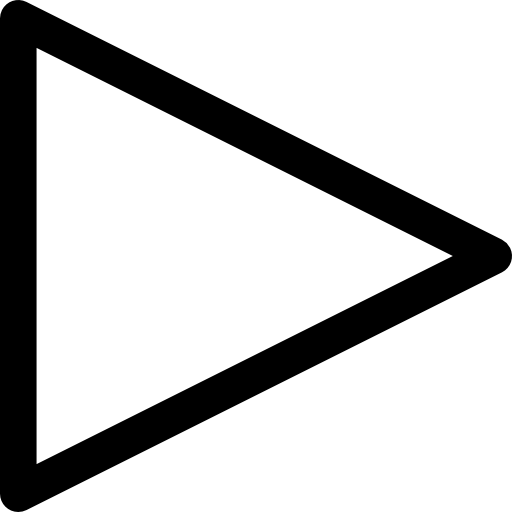

Supplement: Extended Data 2 — MATLAB codes for the software suite. This folder contains the MATLAB codes of the software. Users can run the software and configure none “standard” hardware with MATLAB (R2021a or later) installed on their computers. Download Extended Data 2, ZIP file. [file eneuro-12-ENEURO.0224-25.2025-s004.zip › Extended Data 2/backup/right-button.png]

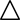

Supplement: Extended Data 2 — MATLAB codes for the software suite. This folder contains the MATLAB codes of the software. Users can run the software and configure none “standard” hardware with MATLAB (R2021a or later) installed on their computers. Download Extended Data 2, ZIP file. [file eneuro-12-ENEURO.0224-25.2025-s004.zip › Extended Data 2/backup/up-button.png]

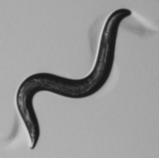

Supplement: Extended Data 2 — MATLAB codes for the software suite. This folder contains the MATLAB codes of the software. Users can run the software and configure none “standard” hardware with MATLAB (R2021a or later) installed on their computers. Download Extended Data 2, ZIP file. [file eneuro-12-ENEURO.0224-25.2025-s004.zip › Extended Data 2/backup/wormrecorder.png]

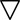

Supplement: Extended Data 2 — MATLAB codes for the software suite. This folder contains the MATLAB codes of the software. Users can run the software and configure none “standard” hardware with MATLAB (R2021a or later) installed on their computers. Download Extended Data 2, ZIP file. [file eneuro-12-ENEURO.0224-25.2025-s004.zip › Extended Data 2/icon/down-button.jpg]

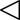

Supplement: Extended Data 2 — MATLAB codes for the software suite. This folder contains the MATLAB codes of the software. Users can run the software and configure none “standard” hardware with MATLAB (R2021a or later) installed on their computers. Download Extended Data 2, ZIP file. [file eneuro-12-ENEURO.0224-25.2025-s004.zip › Extended Data 2/icon/left-button.jpg]

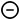

Supplement: Extended Data 2 — MATLAB codes for the software suite. This folder contains the MATLAB codes of the software. Users can run the software and configure none “standard” hardware with MATLAB (R2021a or later) installed on their computers. Download Extended Data 2, ZIP file. [file eneuro-12-ENEURO.0224-25.2025-s004.zip › Extended Data 2/icon/minus-circle.jpg]

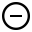

Supplement: Extended Data 2 — MATLAB codes for the software suite. This folder contains the MATLAB codes of the software. Users can run the software and configure none “standard” hardware with MATLAB (R2021a or later) installed on their computers. Download Extended Data 2, ZIP file. [file eneuro-12-ENEURO.0224-25.2025-s004.zip › Extended Data 2/icon/minus-circle.png]

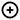

Supplement: Extended Data 2 — MATLAB codes for the software suite. This folder contains the MATLAB codes of the software. Users can run the software and configure none “standard” hardware with MATLAB (R2021a or later) installed on their computers. Download Extended Data 2, ZIP file. [file eneuro-12-ENEURO.0224-25.2025-s004.zip › Extended Data 2/icon/plus-circle.jpg]

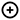

Supplement: Extended Data 2 — MATLAB codes for the software suite. This folder contains the MATLAB codes of the software. Users can run the software and configure none “standard” hardware with MATLAB (R2021a or later) installed on their computers. Download Extended Data 2, ZIP file. [file eneuro-12-ENEURO.0224-25.2025-s004.zip › Extended Data 2/icon/plus-circle.png]

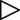

Supplement: Extended Data 2 — MATLAB codes for the software suite. This folder contains the MATLAB codes of the software. Users can run the software and configure none “standard” hardware with MATLAB (R2021a or later) installed on their computers. Download Extended Data 2, ZIP file. [file eneuro-12-ENEURO.0224-25.2025-s004.zip › Extended Data 2/icon/right-button.jpg]

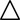

Supplement: Extended Data 2 — MATLAB codes for the software suite. This folder contains the MATLAB codes of the software. Users can run the software and configure none “standard” hardware with MATLAB (R2021a or later) installed on their computers. Download Extended Data 2, ZIP file. [file eneuro-12-ENEURO.0224-25.2025-s004.zip › Extended Data 2/icon/up-button.jpg]

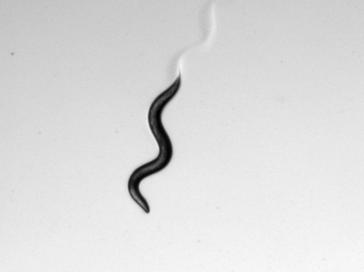

Supplement: Extended Data 4 — A sample WormTracker recording. This folder contains the recording of a wild-type worm (60 seconds, 15 frames per second), along with the associated stage file, time file, and a spline file generated by the Fit Spline module. The images were captured at 50% of the camera's resolution (4 KB/image). Download Extended Data 4, ZIP file. [file eneuro-12-ENEURO.0224-25.2025-s006.zip › Extended Data 4/wt1/L_img00001.jpeg]

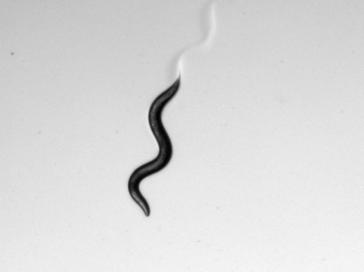

Supplement: Extended Data 4 — A sample WormTracker recording. This folder contains the recording of a wild-type worm (60 seconds, 15 frames per second), along with the associated stage file, time file, and a spline file generated by the Fit Spline module. The images were captured at 50% of the camera's resolution (4 KB/image). Download Extended Data 4, ZIP file. [file eneuro-12-ENEURO.0224-25.2025-s006.zip › Extended Data 4/wt1/L_img00002.jpeg]

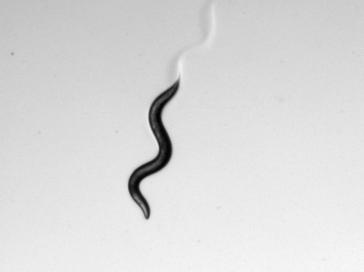

Supplement: Extended Data 4 — A sample WormTracker recording. This folder contains the recording of a wild-type worm (60 seconds, 15 frames per second), along with the associated stage file, time file, and a spline file generated by the Fit Spline module. The images were captured at 50% of the camera's resolution (4 KB/image). Download Extended Data 4, ZIP file. [file eneuro-12-ENEURO.0224-25.2025-s006.zip › Extended Data 4/wt1/L_img00003.jpeg]

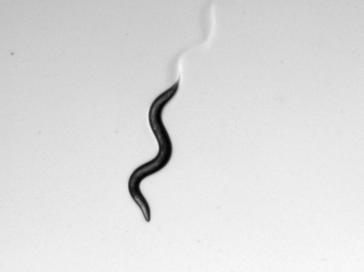

Supplement: Extended Data 4 — A sample WormTracker recording. This folder contains the recording of a wild-type worm (60 seconds, 15 frames per second), along with the associated stage file, time file, and a spline file generated by the Fit Spline module. The images were captured at 50% of the camera's resolution (4 KB/image). Download Extended Data 4, ZIP file. [file eneuro-12-ENEURO.0224-25.2025-s006.zip › Extended Data 4/wt1/L_img00004.jpeg]

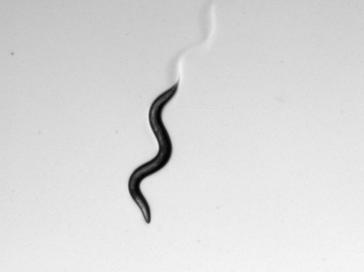

Supplement: Extended Data 4 — A sample WormTracker recording. This folder contains the recording of a wild-type worm (60 seconds, 15 frames per second), along with the associated stage file, time file, and a spline file generated by the Fit Spline module. The images were captured at 50% of the camera's resolution (4 KB/image). Download Extended Data 4, ZIP file. [file eneuro-12-ENEURO.0224-25.2025-s006.zip › Extended Data 4/wt1/L_img00005.jpeg]

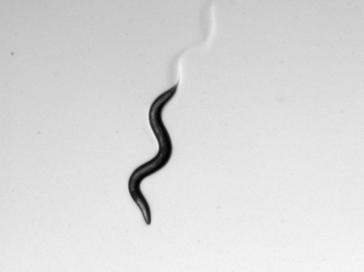

Supplement: Extended Data 4 — A sample WormTracker recording. This folder contains the recording of a wild-type worm (60 seconds, 15 frames per second), along with the associated stage file, time file, and a spline file generated by the Fit Spline module. The images were captured at 50% of the camera's resolution (4 KB/image). Download Extended Data 4, ZIP file. [file eneuro-12-ENEURO.0224-25.2025-s006.zip › Extended Data 4/wt1/L_img00006.jpeg]

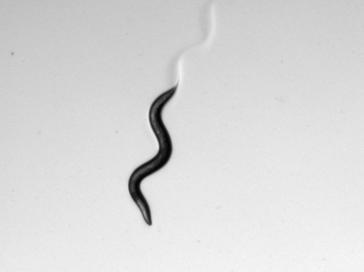

Supplement: Extended Data 4 — A sample WormTracker recording. This folder contains the recording of a wild-type worm (60 seconds, 15 frames per second), along with the associated stage file, time file, and a spline file generated by the Fit Spline module. The images were captured at 50% of the camera's resolution (4 KB/image). Download Extended Data 4, ZIP file. [file eneuro-12-ENEURO.0224-25.2025-s006.zip › Extended Data 4/wt1/L_img00007.jpeg]

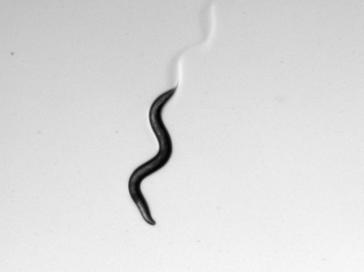

Supplement: Extended Data 4 — A sample WormTracker recording. This folder contains the recording of a wild-type worm (60 seconds, 15 frames per second), along with the associated stage file, time file, and a spline file generated by the Fit Spline module. The images were captured at 50% of the camera's resolution (4 KB/image). Download Extended Data 4, ZIP file. [file eneuro-12-ENEURO.0224-25.2025-s006.zip › Extended Data 4/wt1/L_img00008.jpeg]

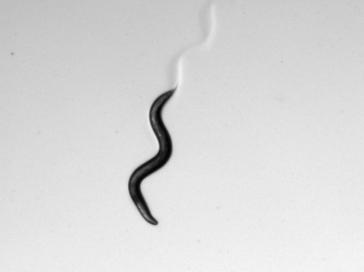

Supplement: Extended Data 4 — A sample WormTracker recording. This folder contains the recording of a wild-type worm (60 seconds, 15 frames per second), along with the associated stage file, time file, and a spline file generated by the Fit Spline module. The images were captured at 50% of the camera's resolution (4 KB/image). Download Extended Data 4, ZIP file. [file eneuro-12-ENEURO.0224-25.2025-s006.zip › Extended Data 4/wt1/L_img00009.jpeg]

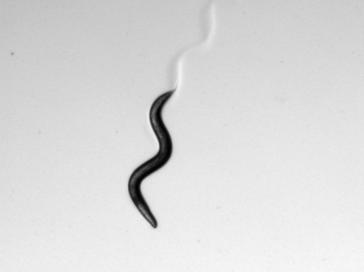

Supplement: Extended Data 4 — A sample WormTracker recording. This folder contains the recording of a wild-type worm (60 seconds, 15 frames per second), along with the associated stage file, time file, and a spline file generated by the Fit Spline module. The images were captured at 50% of the camera's resolution (4 KB/image). Download Extended Data 4, ZIP file. [file eneuro-12-ENEURO.0224-25.2025-s006.zip › Extended Data 4/wt1/L_img00010.jpeg]

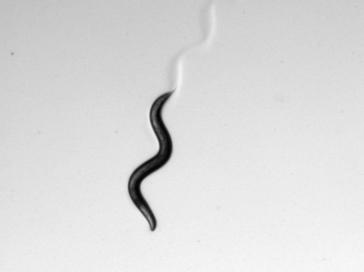

Supplement: Extended Data 4 — A sample WormTracker recording. This folder contains the recording of a wild-type worm (60 seconds, 15 frames per second), along with the associated stage file, time file, and a spline file generated by the Fit Spline module. The images were captured at 50% of the camera's resolution (4 KB/image). Download Extended Data 4, ZIP file. [file eneuro-12-ENEURO.0224-25.2025-s006.zip › Extended Data 4/wt1/L_img00011.jpeg]

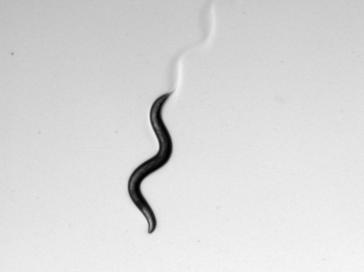

Supplement: Extended Data 4 — A sample WormTracker recording. This folder contains the recording of a wild-type worm (60 seconds, 15 frames per second), along with the associated stage file, time file, and a spline file generated by the Fit Spline module. The images were captured at 50% of the camera's resolution (4 KB/image). Download Extended Data 4, ZIP file. [file eneuro-12-ENEURO.0224-25.2025-s006.zip › Extended Data 4/wt1/L_img00012.jpeg]

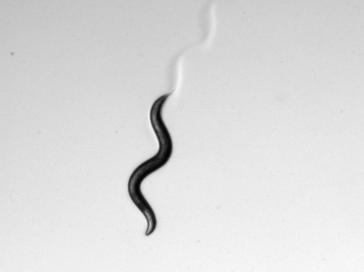

Supplement: Extended Data 4 — A sample WormTracker recording. This folder contains the recording of a wild-type worm (60 seconds, 15 frames per second), along with the associated stage file, time file, and a spline file generated by the Fit Spline module. The images were captured at 50% of the camera's resolution (4 KB/image). Download Extended Data 4, ZIP file. [file eneuro-12-ENEURO.0224-25.2025-s006.zip › Extended Data 4/wt1/L_img00013.jpeg]

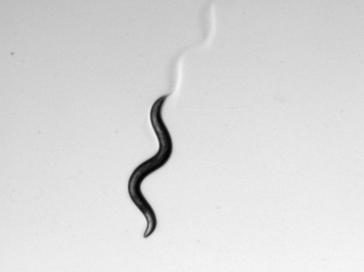

Supplement: Extended Data 4 — A sample WormTracker recording. This folder contains the recording of a wild-type worm (60 seconds, 15 frames per second), along with the associated stage file, time file, and a spline file generated by the Fit Spline module. The images were captured at 50% of the camera's resolution (4 KB/image). Download Extended Data 4, ZIP file. [file eneuro-12-ENEURO.0224-25.2025-s006.zip › Extended Data 4/wt1/L_img00014.jpeg]

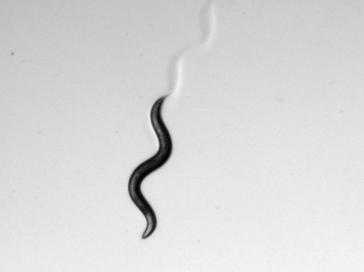

Supplement: Extended Data 4 — A sample WormTracker recording. This folder contains the recording of a wild-type worm (60 seconds, 15 frames per second), along with the associated stage file, time file, and a spline file generated by the Fit Spline module. The images were captured at 50% of the camera's resolution (4 KB/image). Download Extended Data 4, ZIP file. [file eneuro-12-ENEURO.0224-25.2025-s006.zip › Extended Data 4/wt1/L_img00015.jpeg]

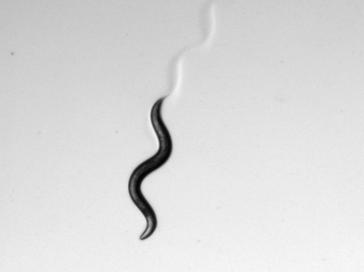

Supplement: Extended Data 4 — A sample WormTracker recording. This folder contains the recording of a wild-type worm (60 seconds, 15 frames per second), along with the associated stage file, time file, and a spline file generated by the Fit Spline module. The images were captured at 50% of the camera's resolution (4 KB/image). Download Extended Data 4, ZIP file. [file eneuro-12-ENEURO.0224-25.2025-s006.zip › Extended Data 4/wt1/L_img00016.jpeg]

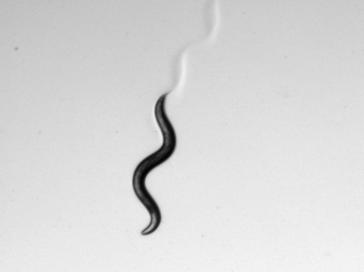

Supplement: Extended Data 4 — A sample WormTracker recording. This folder contains the recording of a wild-type worm (60 seconds, 15 frames per second), along with the associated stage file, time file, and a spline file generated by the Fit Spline module. The images were captured at 50% of the camera's resolution (4 KB/image). Download Extended Data 4, ZIP file. [file eneuro-12-ENEURO.0224-25.2025-s006.zip › Extended Data 4/wt1/L_img00017.jpeg]

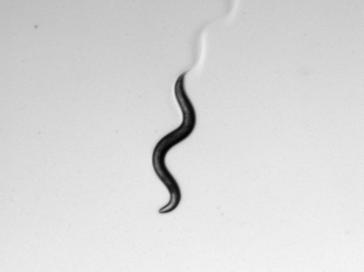

Supplement: Extended Data 4 — A sample WormTracker recording. This folder contains the recording of a wild-type worm (60 seconds, 15 frames per second), along with the associated stage file, time file, and a spline file generated by the Fit Spline module. The images were captured at 50% of the camera's resolution (4 KB/image). Download Extended Data 4, ZIP file. [file eneuro-12-ENEURO.0224-25.2025-s006.zip › Extended Data 4/wt1/L_img00018.jpeg]

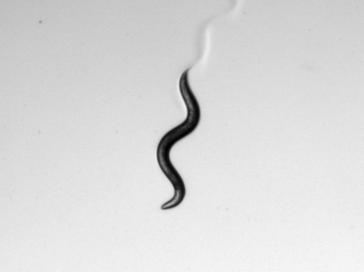

Supplement: Extended Data 4 — A sample WormTracker recording. This folder contains the recording of a wild-type worm (60 seconds, 15 frames per second), along with the associated stage file, time file, and a spline file generated by the Fit Spline module. The images were captured at 50% of the camera's resolution (4 KB/image). Download Extended Data 4, ZIP file. [file eneuro-12-ENEURO.0224-25.2025-s006.zip › Extended Data 4/wt1/L_img00019.jpeg]

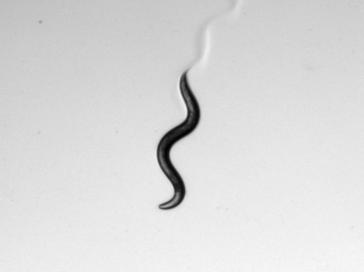

Supplement: Extended Data 4 — A sample WormTracker recording. This folder contains the recording of a wild-type worm (60 seconds, 15 frames per second), along with the associated stage file, time file, and a spline file generated by the Fit Spline module. The images were captured at 50% of the camera's resolution (4 KB/image). Download Extended Data 4, ZIP file. [file eneuro-12-ENEURO.0224-25.2025-s006.zip › Extended Data 4/wt1/L_img00020.jpeg]

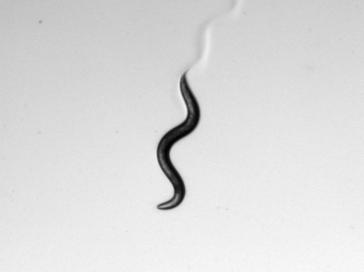

Supplement: Extended Data 4 — A sample WormTracker recording. This folder contains the recording of a wild-type worm (60 seconds, 15 frames per second), along with the associated stage file, time file, and a spline file generated by the Fit Spline module. The images were captured at 50% of the camera's resolution (4 KB/image). Download Extended Data 4, ZIP file. [file eneuro-12-ENEURO.0224-25.2025-s006.zip › Extended Data 4/wt1/L_img00021.jpeg]

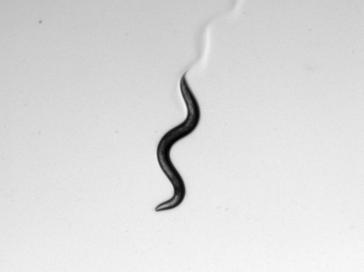

Supplement: Extended Data 4 — A sample WormTracker recording. This folder contains the recording of a wild-type worm (60 seconds, 15 frames per second), along with the associated stage file, time file, and a spline file generated by the Fit Spline module. The images were captured at 50% of the camera's resolution (4 KB/image). Download Extended Data 4, ZIP file. [file eneuro-12-ENEURO.0224-25.2025-s006.zip › Extended Data 4/wt1/L_img00022.jpeg]

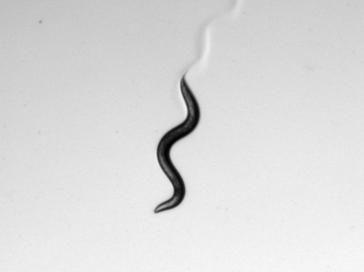

Supplement: Extended Data 4 — A sample WormTracker recording. This folder contains the recording of a wild-type worm (60 seconds, 15 frames per second), along with the associated stage file, time file, and a spline file generated by the Fit Spline module. The images were captured at 50% of the camera's resolution (4 KB/image). Download Extended Data 4, ZIP file. [file eneuro-12-ENEURO.0224-25.2025-s006.zip › Extended Data 4/wt1/L_img00023.jpeg]

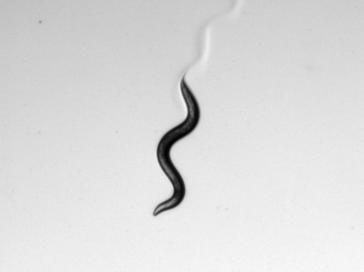

Supplement: Extended Data 4 — A sample WormTracker recording. This folder contains the recording of a wild-type worm (60 seconds, 15 frames per second), along with the associated stage file, time file, and a spline file generated by the Fit Spline module. The images were captured at 50% of the camera's resolution (4 KB/image). Download Extended Data 4, ZIP file. [file eneuro-12-ENEURO.0224-25.2025-s006.zip › Extended Data 4/wt1/L_img00024.jpeg]

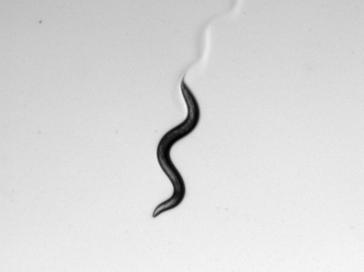

Supplement: Extended Data 4 — A sample WormTracker recording. This folder contains the recording of a wild-type worm (60 seconds, 15 frames per second), along with the associated stage file, time file, and a spline file generated by the Fit Spline module. The images were captured at 50% of the camera's resolution (4 KB/image). Download Extended Data 4, ZIP file. [file eneuro-12-ENEURO.0224-25.2025-s006.zip › Extended Data 4/wt1/L_img00025.jpeg]

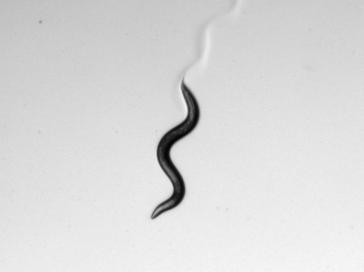

Supplement: Extended Data 4 — A sample WormTracker recording. This folder contains the recording of a wild-type worm (60 seconds, 15 frames per second), along with the associated stage file, time file, and a spline file generated by the Fit Spline module. The images were captured at 50% of the camera's resolution (4 KB/image). Download Extended Data 4, ZIP file. [file eneuro-12-ENEURO.0224-25.2025-s006.zip › Extended Data 4/wt1/L_img00026.jpeg]

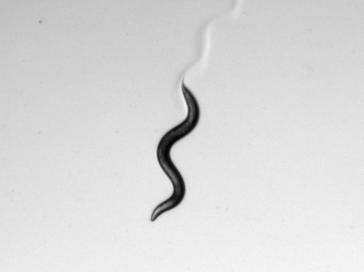

Supplement: Extended Data 4 — A sample WormTracker recording. This folder contains the recording of a wild-type worm (60 seconds, 15 frames per second), along with the associated stage file, time file, and a spline file generated by the Fit Spline module. The images were captured at 50% of the camera's resolution (4 KB/image). Download Extended Data 4, ZIP file. [file eneuro-12-ENEURO.0224-25.2025-s006.zip › Extended Data 4/wt1/L_img00027.jpeg]

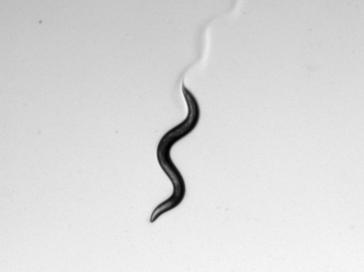

Supplement: Extended Data 4 — A sample WormTracker recording. This folder contains the recording of a wild-type worm (60 seconds, 15 frames per second), along with the associated stage file, time file, and a spline file generated by the Fit Spline module. The images were captured at 50% of the camera's resolution (4 KB/image). Download Extended Data 4, ZIP file. [file eneuro-12-ENEURO.0224-25.2025-s006.zip › Extended Data 4/wt1/L_img00028.jpeg]

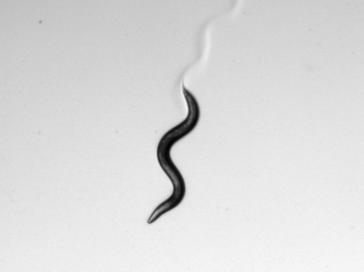

Supplement: Extended Data 4 — A sample WormTracker recording. This folder contains the recording of a wild-type worm (60 seconds, 15 frames per second), along with the associated stage file, time file, and a spline file generated by the Fit Spline module. The images were captured at 50% of the camera's resolution (4 KB/image). Download Extended Data 4, ZIP file. [file eneuro-12-ENEURO.0224-25.2025-s006.zip › Extended Data 4/wt1/L_img00029.jpeg]

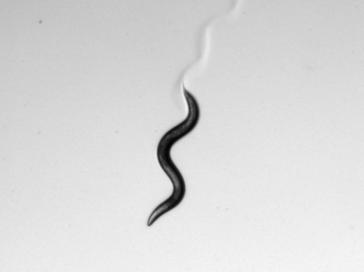

Supplement: Extended Data 4 — A sample WormTracker recording. This folder contains the recording of a wild-type worm (60 seconds, 15 frames per second), along with the associated stage file, time file, and a spline file generated by the Fit Spline module. The images were captured at 50% of the camera's resolution (4 KB/image). Download Extended Data 4, ZIP file. [file eneuro-12-ENEURO.0224-25.2025-s006.zip › Extended Data 4/wt1/L_img00030.jpeg]

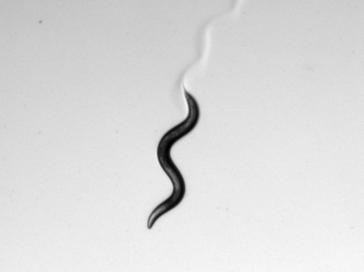

Supplement: Extended Data 4 — A sample WormTracker recording. This folder contains the recording of a wild-type worm (60 seconds, 15 frames per second), along with the associated stage file, time file, and a spline file generated by the Fit Spline module. The images were captured at 50% of the camera's resolution (4 KB/image). Download Extended Data 4, ZIP file. [file eneuro-12-ENEURO.0224-25.2025-s006.zip › Extended Data 4/wt1/L_img00031.jpeg]

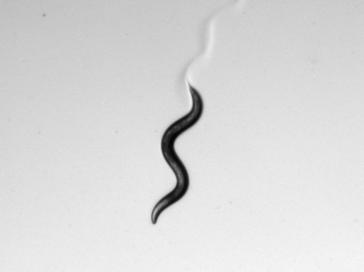

Supplement: Extended Data 4 — A sample WormTracker recording. This folder contains the recording of a wild-type worm (60 seconds, 15 frames per second), along with the associated stage file, time file, and a spline file generated by the Fit Spline module. The images were captured at 50% of the camera's resolution (4 KB/image). Download Extended Data 4, ZIP file. [file eneuro-12-ENEURO.0224-25.2025-s006.zip › Extended Data 4/wt1/L_img00032.jpeg]

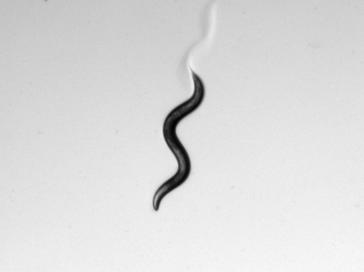

Supplement: Extended Data 4 — A sample WormTracker recording. This folder contains the recording of a wild-type worm (60 seconds, 15 frames per second), along with the associated stage file, time file, and a spline file generated by the Fit Spline module. The images were captured at 50% of the camera's resolution (4 KB/image). Download Extended Data 4, ZIP file. [file eneuro-12-ENEURO.0224-25.2025-s006.zip › Extended Data 4/wt1/L_img00033.jpeg]

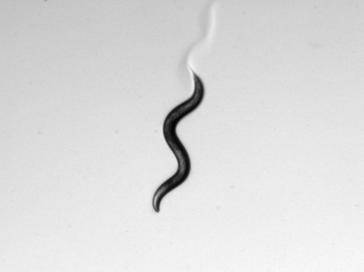

Supplement: Extended Data 4 — A sample WormTracker recording. This folder contains the recording of a wild-type worm (60 seconds, 15 frames per second), along with the associated stage file, time file, and a spline file generated by the Fit Spline module. The images were captured at 50% of the camera's resolution (4 KB/image). Download Extended Data 4, ZIP file. [file eneuro-12-ENEURO.0224-25.2025-s006.zip › Extended Data 4/wt1/L_img00034.jpeg]

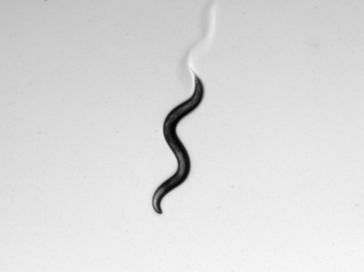

Supplement: Extended Data 4 — A sample WormTracker recording. This folder contains the recording of a wild-type worm (60 seconds, 15 frames per second), along with the associated stage file, time file, and a spline file generated by the Fit Spline module. The images were captured at 50% of the camera's resolution (4 KB/image). Download Extended Data 4, ZIP file. [file eneuro-12-ENEURO.0224-25.2025-s006.zip › Extended Data 4/wt1/L_img00035.jpeg]

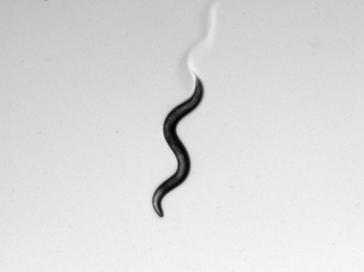

Supplement: Extended Data 4 — A sample WormTracker recording. This folder contains the recording of a wild-type worm (60 seconds, 15 frames per second), along with the associated stage file, time file, and a spline file generated by the Fit Spline module. The images were captured at 50% of the camera's resolution (4 KB/image). Download Extended Data 4, ZIP file. [file eneuro-12-ENEURO.0224-25.2025-s006.zip › Extended Data 4/wt1/L_img00036.jpeg]

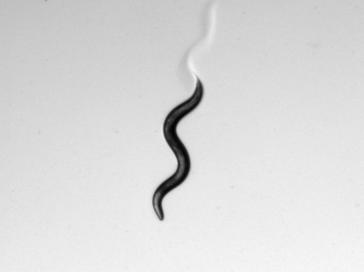

Supplement: Extended Data 4 — A sample WormTracker recording. This folder contains the recording of a wild-type worm (60 seconds, 15 frames per second), along with the associated stage file, time file, and a spline file generated by the Fit Spline module. The images were captured at 50% of the camera's resolution (4 KB/image). Download Extended Data 4, ZIP file. [file eneuro-12-ENEURO.0224-25.2025-s006.zip › Extended Data 4/wt1/L_img00037.jpeg]

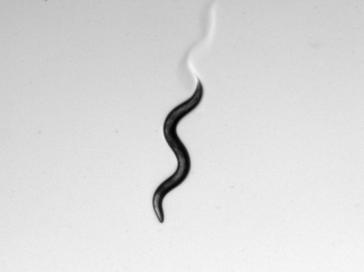

Supplement: Extended Data 4 — A sample WormTracker recording. This folder contains the recording of a wild-type worm (60 seconds, 15 frames per second), along with the associated stage file, time file, and a spline file generated by the Fit Spline module. The images were captured at 50% of the camera's resolution (4 KB/image). Download Extended Data 4, ZIP file. [file eneuro-12-ENEURO.0224-25.2025-s006.zip › Extended Data 4/wt1/L_img00038.jpeg]

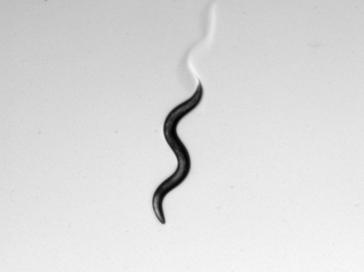

Supplement: Extended Data 4 — A sample WormTracker recording. This folder contains the recording of a wild-type worm (60 seconds, 15 frames per second), along with the associated stage file, time file, and a spline file generated by the Fit Spline module. The images were captured at 50% of the camera's resolution (4 KB/image). Download Extended Data 4, ZIP file. [file eneuro-12-ENEURO.0224-25.2025-s006.zip › Extended Data 4/wt1/L_img00039.jpeg]

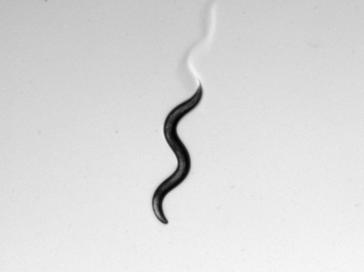

Supplement: Extended Data 4 — A sample WormTracker recording. This folder contains the recording of a wild-type worm (60 seconds, 15 frames per second), along with the associated stage file, time file, and a spline file generated by the Fit Spline module. The images were captured at 50% of the camera's resolution (4 KB/image). Download Extended Data 4, ZIP file. [file eneuro-12-ENEURO.0224-25.2025-s006.zip › Extended Data 4/wt1/L_img00040.jpeg]

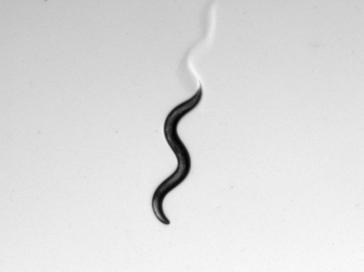

Supplement: Extended Data 4 — A sample WormTracker recording. This folder contains the recording of a wild-type worm (60 seconds, 15 frames per second), along with the associated stage file, time file, and a spline file generated by the Fit Spline module. The images were captured at 50% of the camera's resolution (4 KB/image). Download Extended Data 4, ZIP file. [file eneuro-12-ENEURO.0224-25.2025-s006.zip › Extended Data 4/wt1/L_img00041.jpeg]

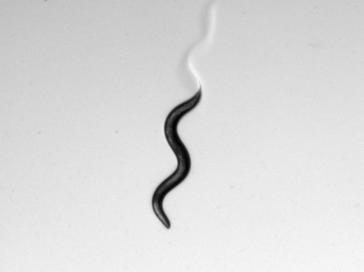

Supplement: Extended Data 4 — A sample WormTracker recording. This folder contains the recording of a wild-type worm (60 seconds, 15 frames per second), along with the associated stage file, time file, and a spline file generated by the Fit Spline module. The images were captured at 50% of the camera's resolution (4 KB/image). Download Extended Data 4, ZIP file. [file eneuro-12-ENEURO.0224-25.2025-s006.zip › Extended Data 4/wt1/L_img00042.jpeg]

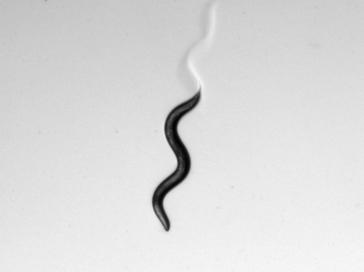

Supplement: Extended Data 4 — A sample WormTracker recording. This folder contains the recording of a wild-type worm (60 seconds, 15 frames per second), along with the associated stage file, time file, and a spline file generated by the Fit Spline module. The images were captured at 50% of the camera's resolution (4 KB/image). Download Extended Data 4, ZIP file. [file eneuro-12-ENEURO.0224-25.2025-s006.zip › Extended Data 4/wt1/L_img00043.jpeg]

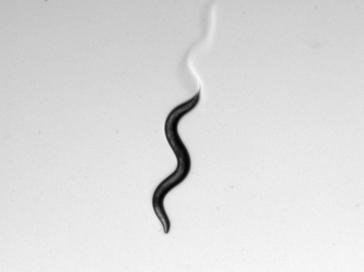

Supplement: Extended Data 4 — A sample WormTracker recording. This folder contains the recording of a wild-type worm (60 seconds, 15 frames per second), along with the associated stage file, time file, and a spline file generated by the Fit Spline module. The images were captured at 50% of the camera's resolution (4 KB/image). Download Extended Data 4, ZIP file. [file eneuro-12-ENEURO.0224-25.2025-s006.zip › Extended Data 4/wt1/L_img00044.jpeg]

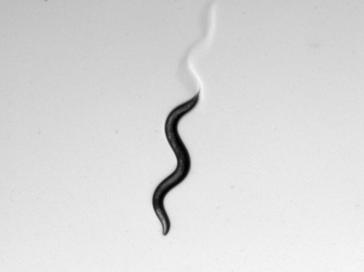

Supplement: Extended Data 4 — A sample WormTracker recording. This folder contains the recording of a wild-type worm (60 seconds, 15 frames per second), along with the associated stage file, time file, and a spline file generated by the Fit Spline module. The images were captured at 50% of the camera's resolution (4 KB/image). Download Extended Data 4, ZIP file. [file eneuro-12-ENEURO.0224-25.2025-s006.zip › Extended Data 4/wt1/L_img00045.jpeg]

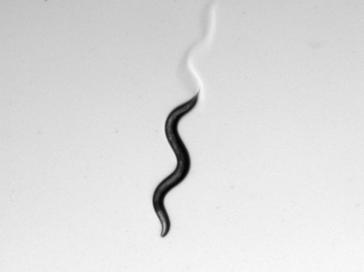

Supplement: Extended Data 4 — A sample WormTracker recording. This folder contains the recording of a wild-type worm (60 seconds, 15 frames per second), along with the associated stage file, time file, and a spline file generated by the Fit Spline module. The images were captured at 50% of the camera's resolution (4 KB/image). Download Extended Data 4, ZIP file. [file eneuro-12-ENEURO.0224-25.2025-s006.zip › Extended Data 4/wt1/L_img00046.jpeg]

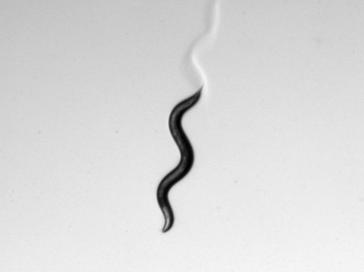

Supplement: Extended Data 4 — A sample WormTracker recording. This folder contains the recording of a wild-type worm (60 seconds, 15 frames per second), along with the associated stage file, time file, and a spline file generated by the Fit Spline module. The images were captured at 50% of the camera's resolution (4 KB/image). Download Extended Data 4, ZIP file. [file eneuro-12-ENEURO.0224-25.2025-s006.zip › Extended Data 4/wt1/L_img00047.jpeg]

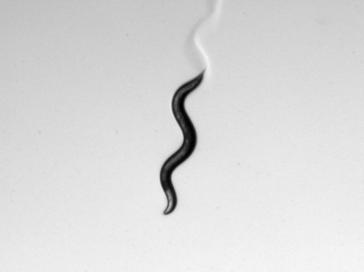

Supplement: Extended Data 4 — A sample WormTracker recording. This folder contains the recording of a wild-type worm (60 seconds, 15 frames per second), along with the associated stage file, time file, and a spline file generated by the Fit Spline module. The images were captured at 50% of the camera's resolution (4 KB/image). Download Extended Data 4, ZIP file. [file eneuro-12-ENEURO.0224-25.2025-s006.zip › Extended Data 4/wt1/L_img00048.jpeg]

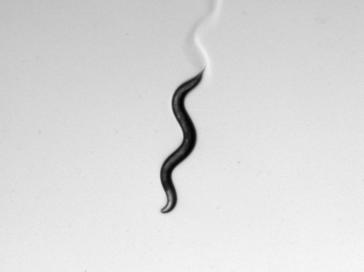

Supplement: Extended Data 4 — A sample WormTracker recording. This folder contains the recording of a wild-type worm (60 seconds, 15 frames per second), along with the associated stage file, time file, and a spline file generated by the Fit Spline module. The images were captured at 50% of the camera's resolution (4 KB/image). Download Extended Data 4, ZIP file. [file eneuro-12-ENEURO.0224-25.2025-s006.zip › Extended Data 4/wt1/L_img00049.jpeg]

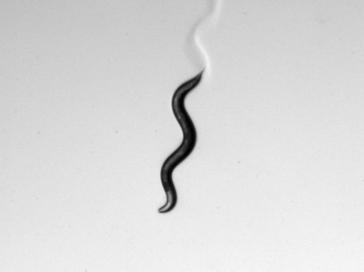

Supplement: Extended Data 4 — A sample WormTracker recording. This folder contains the recording of a wild-type worm (60 seconds, 15 frames per second), along with the associated stage file, time file, and a spline file generated by the Fit Spline module. The images were captured at 50% of the camera's resolution (4 KB/image). Download Extended Data 4, ZIP file. [file eneuro-12-ENEURO.0224-25.2025-s006.zip › Extended Data 4/wt1/L_img00050.jpeg]

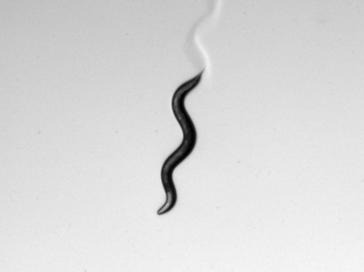

Supplement: Extended Data 4 — A sample WormTracker recording. This folder contains the recording of a wild-type worm (60 seconds, 15 frames per second), along with the associated stage file, time file, and a spline file generated by the Fit Spline module. The images were captured at 50% of the camera's resolution (4 KB/image). Download Extended Data 4, ZIP file. [file eneuro-12-ENEURO.0224-25.2025-s006.zip › Extended Data 4/wt1/L_img00051.jpeg]

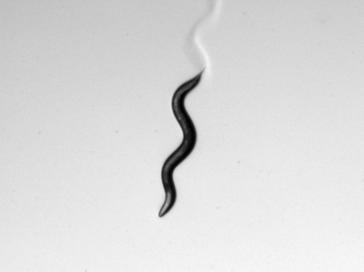

Supplement: Extended Data 4 — A sample WormTracker recording. This folder contains the recording of a wild-type worm (60 seconds, 15 frames per second), along with the associated stage file, time file, and a spline file generated by the Fit Spline module. The images were captured at 50% of the camera's resolution (4 KB/image). Download Extended Data 4, ZIP file. [file eneuro-12-ENEURO.0224-25.2025-s006.zip › Extended Data 4/wt1/L_img00052.jpeg]

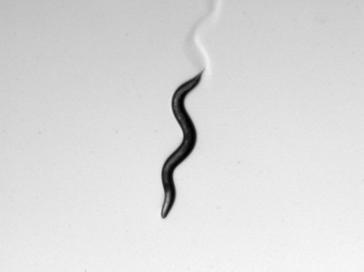

Supplement: Extended Data 4 — A sample WormTracker recording. This folder contains the recording of a wild-type worm (60 seconds, 15 frames per second), along with the associated stage file, time file, and a spline file generated by the Fit Spline module. The images were captured at 50% of the camera's resolution (4 KB/image). Download Extended Data 4, ZIP file. [file eneuro-12-ENEURO.0224-25.2025-s006.zip › Extended Data 4/wt1/L_img00053.jpeg]

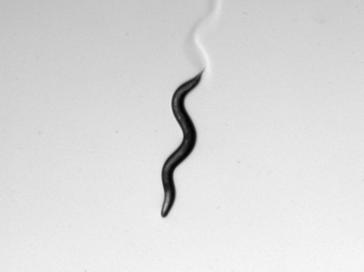

Supplement: Extended Data 4 — A sample WormTracker recording. This folder contains the recording of a wild-type worm (60 seconds, 15 frames per second), along with the associated stage file, time file, and a spline file generated by the Fit Spline module. The images were captured at 50% of the camera's resolution (4 KB/image). Download Extended Data 4, ZIP file. [file eneuro-12-ENEURO.0224-25.2025-s006.zip › Extended Data 4/wt1/L_img00054.jpeg]

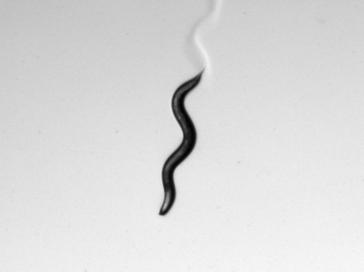

Supplement: Extended Data 4 — A sample WormTracker recording. This folder contains the recording of a wild-type worm (60 seconds, 15 frames per second), along with the associated stage file, time file, and a spline file generated by the Fit Spline module. The images were captured at 50% of the camera's resolution (4 KB/image). Download Extended Data 4, ZIP file. [file eneuro-12-ENEURO.0224-25.2025-s006.zip › Extended Data 4/wt1/L_img00055.jpeg]

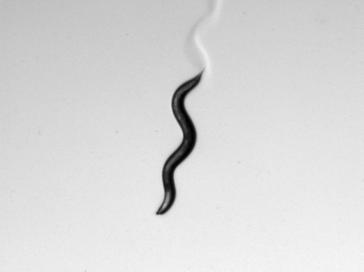

Supplement: Extended Data 4 — A sample WormTracker recording. This folder contains the recording of a wild-type worm (60 seconds, 15 frames per second), along with the associated stage file, time file, and a spline file generated by the Fit Spline module. The images were captured at 50% of the camera's resolution (4 KB/image). Download Extended Data 4, ZIP file. [file eneuro-12-ENEURO.0224-25.2025-s006.zip › Extended Data 4/wt1/L_img00056.jpeg]

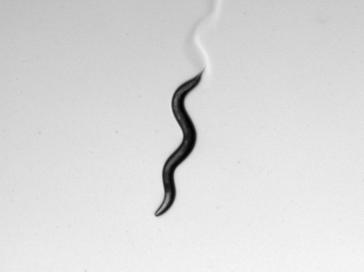

Supplement: Extended Data 4 — A sample WormTracker recording. This folder contains the recording of a wild-type worm (60 seconds, 15 frames per second), along with the associated stage file, time file, and a spline file generated by the Fit Spline module. The images were captured at 50% of the camera's resolution (4 KB/image). Download Extended Data 4, ZIP file. [file eneuro-12-ENEURO.0224-25.2025-s006.zip › Extended Data 4/wt1/L_img00057.jpeg]

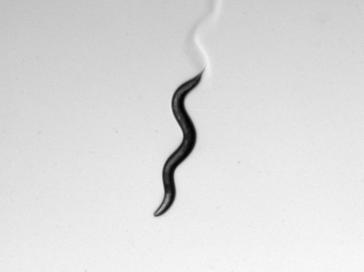

Supplement: Extended Data 4 — A sample WormTracker recording. This folder contains the recording of a wild-type worm (60 seconds, 15 frames per second), along with the associated stage file, time file, and a spline file generated by the Fit Spline module. The images were captured at 50% of the camera's resolution (4 KB/image). Download Extended Data 4, ZIP file. [file eneuro-12-ENEURO.0224-25.2025-s006.zip › Extended Data 4/wt1/L_img00058.jpeg]

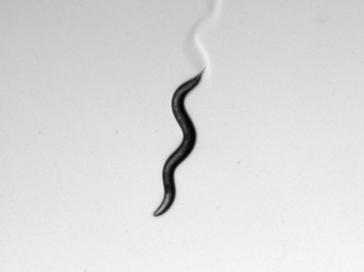

Supplement: Extended Data 4 — A sample WormTracker recording. This folder contains the recording of a wild-type worm (60 seconds, 15 frames per second), along with the associated stage file, time file, and a spline file generated by the Fit Spline module. The images were captured at 50% of the camera's resolution (4 KB/image). Download Extended Data 4, ZIP file. [file eneuro-12-ENEURO.0224-25.2025-s006.zip › Extended Data 4/wt1/L_img00059.jpeg]

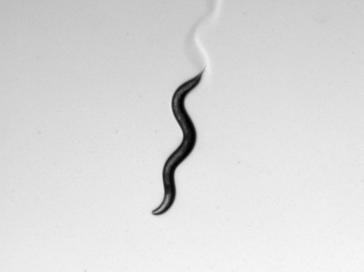

Supplement: Extended Data 4 — A sample WormTracker recording. This folder contains the recording of a wild-type worm (60 seconds, 15 frames per second), along with the associated stage file, time file, and a spline file generated by the Fit Spline module. The images were captured at 50% of the camera's resolution (4 KB/image). Download Extended Data 4, ZIP file. [file eneuro-12-ENEURO.0224-25.2025-s006.zip › Extended Data 4/wt1/L_img00060.jpeg]

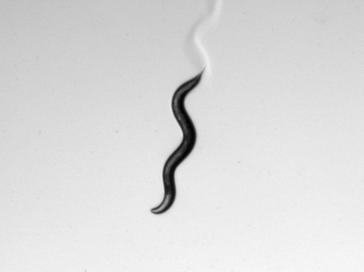

Supplement: Extended Data 4 — A sample WormTracker recording. This folder contains the recording of a wild-type worm (60 seconds, 15 frames per second), along with the associated stage file, time file, and a spline file generated by the Fit Spline module. The images were captured at 50% of the camera's resolution (4 KB/image). Download Extended Data 4, ZIP file. [file eneuro-12-ENEURO.0224-25.2025-s006.zip › Extended Data 4/wt1/L_img00061.jpeg]

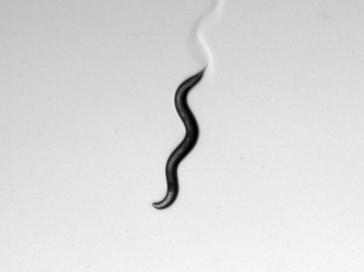

Supplement: Extended Data 4 — A sample WormTracker recording. This folder contains the recording of a wild-type worm (60 seconds, 15 frames per second), along with the associated stage file, time file, and a spline file generated by the Fit Spline module. The images were captured at 50% of the camera's resolution (4 KB/image). Download Extended Data 4, ZIP file. [file eneuro-12-ENEURO.0224-25.2025-s006.zip › Extended Data 4/wt1/L_img00062.jpeg]

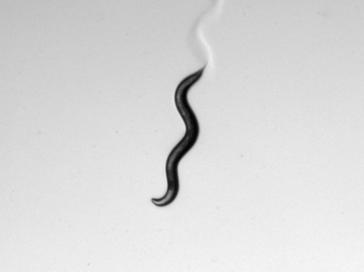

Supplement: Extended Data 4 — A sample WormTracker recording. This folder contains the recording of a wild-type worm (60 seconds, 15 frames per second), along with the associated stage file, time file, and a spline file generated by the Fit Spline module. The images were captured at 50% of the camera's resolution (4 KB/image). Download Extended Data 4, ZIP file. [file eneuro-12-ENEURO.0224-25.2025-s006.zip › Extended Data 4/wt1/L_img00063.jpeg]

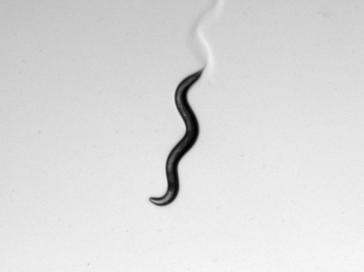

Supplement: Extended Data 4 — A sample WormTracker recording. This folder contains the recording of a wild-type worm (60 seconds, 15 frames per second), along with the associated stage file, time file, and a spline file generated by the Fit Spline module. The images were captured at 50% of the camera's resolution (4 KB/image). Download Extended Data 4, ZIP file. [file eneuro-12-ENEURO.0224-25.2025-s006.zip › Extended Data 4/wt1/L_img00064.jpeg]

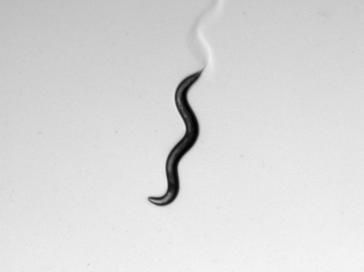

Supplement: Extended Data 4 — A sample WormTracker recording. This folder contains the recording of a wild-type worm (60 seconds, 15 frames per second), along with the associated stage file, time file, and a spline file generated by the Fit Spline module. The images were captured at 50% of the camera's resolution (4 KB/image). Download Extended Data 4, ZIP file. [file eneuro-12-ENEURO.0224-25.2025-s006.zip › Extended Data 4/wt1/L_img00065.jpeg]

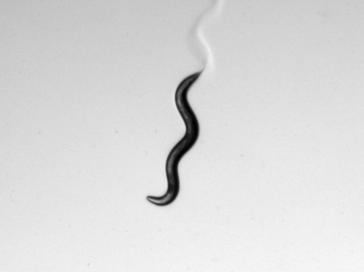

Supplement: Extended Data 4 — A sample WormTracker recording. This folder contains the recording of a wild-type worm (60 seconds, 15 frames per second), along with the associated stage file, time file, and a spline file generated by the Fit Spline module. The images were captured at 50% of the camera's resolution (4 KB/image). Download Extended Data 4, ZIP file. [file eneuro-12-ENEURO.0224-25.2025-s006.zip › Extended Data 4/wt1/L_img00066.jpeg]

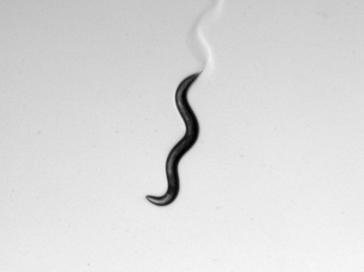

Supplement: Extended Data 4 — A sample WormTracker recording. This folder contains the recording of a wild-type worm (60 seconds, 15 frames per second), along with the associated stage file, time file, and a spline file generated by the Fit Spline module. The images were captured at 50% of the camera's resolution (4 KB/image). Download Extended Data 4, ZIP file. [file eneuro-12-ENEURO.0224-25.2025-s006.zip › Extended Data 4/wt1/L_img00067.jpeg]

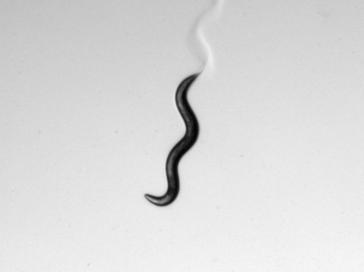

Supplement: Extended Data 4 — A sample WormTracker recording. This folder contains the recording of a wild-type worm (60 seconds, 15 frames per second), along with the associated stage file, time file, and a spline file generated by the Fit Spline module. The images were captured at 50% of the camera's resolution (4 KB/image). Download Extended Data 4, ZIP file. [file eneuro-12-ENEURO.0224-25.2025-s006.zip › Extended Data 4/wt1/L_img00068.jpeg]

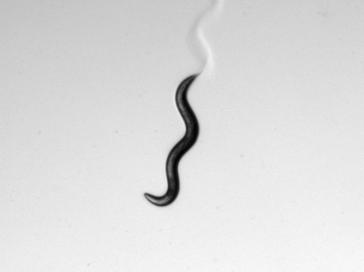

Supplement: Extended Data 4 — A sample WormTracker recording. This folder contains the recording of a wild-type worm (60 seconds, 15 frames per second), along with the associated stage file, time file, and a spline file generated by the Fit Spline module. The images were captured at 50% of the camera's resolution (4 KB/image). Download Extended Data 4, ZIP file. [file eneuro-12-ENEURO.0224-25.2025-s006.zip › Extended Data 4/wt1/L_img00069.jpeg]

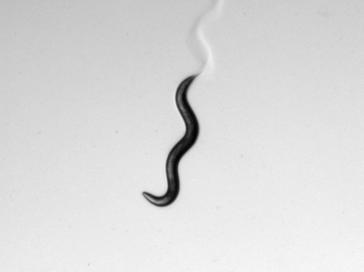

Supplement: Extended Data 4 — A sample WormTracker recording. This folder contains the recording of a wild-type worm (60 seconds, 15 frames per second), along with the associated stage file, time file, and a spline file generated by the Fit Spline module. The images were captured at 50% of the camera's resolution (4 KB/image). Download Extended Data 4, ZIP file. [file eneuro-12-ENEURO.0224-25.2025-s006.zip › Extended Data 4/wt1/L_img00070.jpeg]

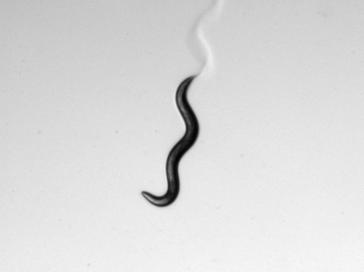

Supplement: Extended Data 4 — A sample WormTracker recording. This folder contains the recording of a wild-type worm (60 seconds, 15 frames per second), along with the associated stage file, time file, and a spline file generated by the Fit Spline module. The images were captured at 50% of the camera's resolution (4 KB/image). Download Extended Data 4, ZIP file. [file eneuro-12-ENEURO.0224-25.2025-s006.zip › Extended Data 4/wt1/L_img00071.jpeg]

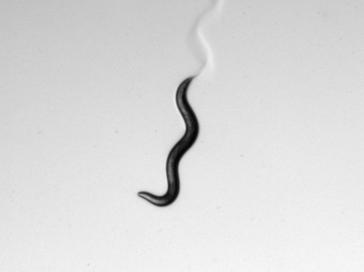

Supplement: Extended Data 4 — A sample WormTracker recording. This folder contains the recording of a wild-type worm (60 seconds, 15 frames per second), along with the associated stage file, time file, and a spline file generated by the Fit Spline module. The images were captured at 50% of the camera's resolution (4 KB/image). Download Extended Data 4, ZIP file. [file eneuro-12-ENEURO.0224-25.2025-s006.zip › Extended Data 4/wt1/L_img00072.jpeg]

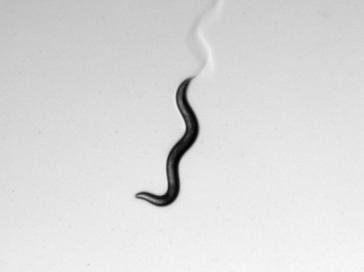

Supplement: Extended Data 4 — A sample WormTracker recording. This folder contains the recording of a wild-type worm (60 seconds, 15 frames per second), along with the associated stage file, time file, and a spline file generated by the Fit Spline module. The images were captured at 50% of the camera's resolution (4 KB/image). Download Extended Data 4, ZIP file. [file eneuro-12-ENEURO.0224-25.2025-s006.zip › Extended Data 4/wt1/L_img00073.jpeg]

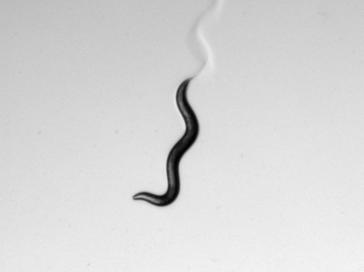

Supplement: Extended Data 4 — A sample WormTracker recording. This folder contains the recording of a wild-type worm (60 seconds, 15 frames per second), along with the associated stage file, time file, and a spline file generated by the Fit Spline module. The images were captured at 50% of the camera's resolution (4 KB/image). Download Extended Data 4, ZIP file. [file eneuro-12-ENEURO.0224-25.2025-s006.zip › Extended Data 4/wt1/L_img00074.jpeg]

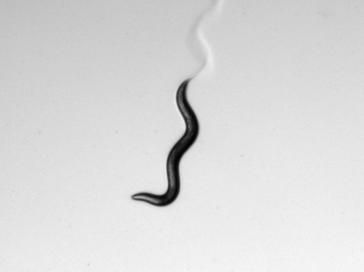

Supplement: Extended Data 4 — A sample WormTracker recording. This folder contains the recording of a wild-type worm (60 seconds, 15 frames per second), along with the associated stage file, time file, and a spline file generated by the Fit Spline module. The images were captured at 50% of the camera's resolution (4 KB/image). Download Extended Data 4, ZIP file. [file eneuro-12-ENEURO.0224-25.2025-s006.zip › Extended Data 4/wt1/L_img00075.jpeg]

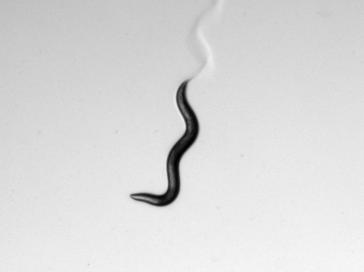

Supplement: Extended Data 4 — A sample WormTracker recording. This folder contains the recording of a wild-type worm (60 seconds, 15 frames per second), along with the associated stage file, time file, and a spline file generated by the Fit Spline module. The images were captured at 50% of the camera's resolution (4 KB/image). Download Extended Data 4, ZIP file. [file eneuro-12-ENEURO.0224-25.2025-s006.zip › Extended Data 4/wt1/L_img00076.jpeg]

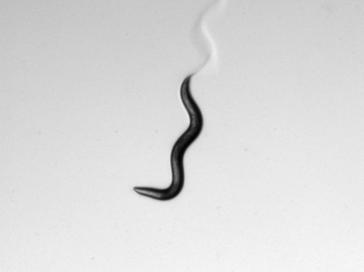

Supplement: Extended Data 4 — A sample WormTracker recording. This folder contains the recording of a wild-type worm (60 seconds, 15 frames per second), along with the associated stage file, time file, and a spline file generated by the Fit Spline module. The images were captured at 50% of the camera's resolution (4 KB/image). Download Extended Data 4, ZIP file. [file eneuro-12-ENEURO.0224-25.2025-s006.zip › Extended Data 4/wt1/L_img00077.jpeg]

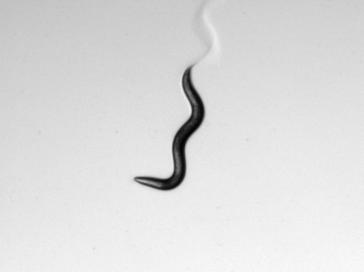

Supplement: Extended Data 4 — A sample WormTracker recording. This folder contains the recording of a wild-type worm (60 seconds, 15 frames per second), along with the associated stage file, time file, and a spline file generated by the Fit Spline module. The images were captured at 50% of the camera's resolution (4 KB/image). Download Extended Data 4, ZIP file. [file eneuro-12-ENEURO.0224-25.2025-s006.zip › Extended Data 4/wt1/L_img00078.jpeg]

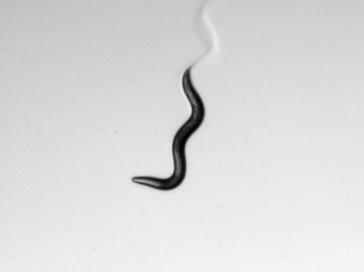

Supplement: Extended Data 4 — A sample WormTracker recording. This folder contains the recording of a wild-type worm (60 seconds, 15 frames per second), along with the associated stage file, time file, and a spline file generated by the Fit Spline module. The images were captured at 50% of the camera's resolution (4 KB/image). Download Extended Data 4, ZIP file. [file eneuro-12-ENEURO.0224-25.2025-s006.zip › Extended Data 4/wt1/L_img00079.jpeg]

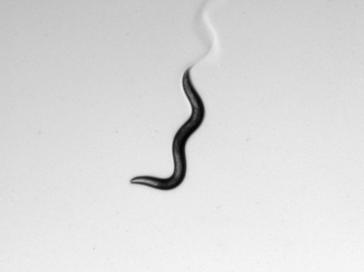

Supplement: Extended Data 4 — A sample WormTracker recording. This folder contains the recording of a wild-type worm (60 seconds, 15 frames per second), along with the associated stage file, time file, and a spline file generated by the Fit Spline module. The images were captured at 50% of the camera's resolution (4 KB/image). Download Extended Data 4, ZIP file. [file eneuro-12-ENEURO.0224-25.2025-s006.zip › Extended Data 4/wt1/L_img00080.jpeg]

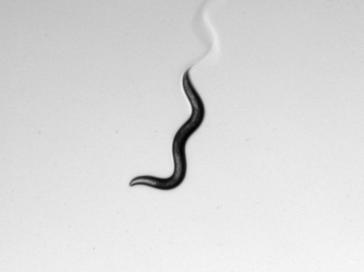

Supplement: Extended Data 4 — A sample WormTracker recording. This folder contains the recording of a wild-type worm (60 seconds, 15 frames per second), along with the associated stage file, time file, and a spline file generated by the Fit Spline module. The images were captured at 50% of the camera's resolution (4 KB/image). Download Extended Data 4, ZIP file. [file eneuro-12-ENEURO.0224-25.2025-s006.zip › Extended Data 4/wt1/L_img00081.jpeg]

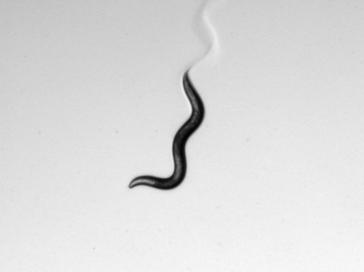

Supplement: Extended Data 4 — A sample WormTracker recording. This folder contains the recording of a wild-type worm (60 seconds, 15 frames per second), along with the associated stage file, time file, and a spline file generated by the Fit Spline module. The images were captured at 50% of the camera's resolution (4 KB/image). Download Extended Data 4, ZIP file. [file eneuro-12-ENEURO.0224-25.2025-s006.zip › Extended Data 4/wt1/L_img00082.jpeg]

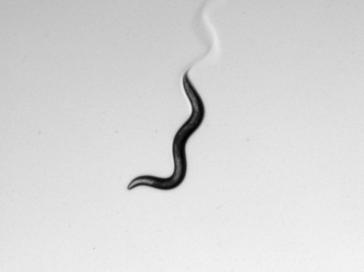

Supplement: Extended Data 4 — A sample WormTracker recording. This folder contains the recording of a wild-type worm (60 seconds, 15 frames per second), along with the associated stage file, time file, and a spline file generated by the Fit Spline module. The images were captured at 50% of the camera's resolution (4 KB/image). Download Extended Data 4, ZIP file. [file eneuro-12-ENEURO.0224-25.2025-s006.zip › Extended Data 4/wt1/L_img00083.jpeg]
