# Supplementary material for: Track-A-Worm 2.0: A Software Suite for Quantifying Properties of C. elegans Locomotion, Bending, Sleep, and Action Potentials
Source: eNeuro. 2025 Aug 13;12(8):ENEURO.0224-25.2025. doi: 10.1523/ENEURO.0224-25.2025 (PMC12393025; doi:10.1523/ENEURO.0224-25.2025)
Supplement: Extended Data 3 — Installation instructions for the Standalone version and hardware drivers. This file provides a step-by-step guide for installing and launching the standalone version of the software suite, as well as installing the necessary hardware drivers. It also explains how to verify proper stage operation and how to recenter the stage if needed. Download Extended Data 3, DOCX file. [file eneuro-12-ENEURO.0224-25.2025-s005.docx]

***Standalone* version software installation**

1. Run the *MyAppInstaller_web.exe* file. After the installer window opens, click Next.


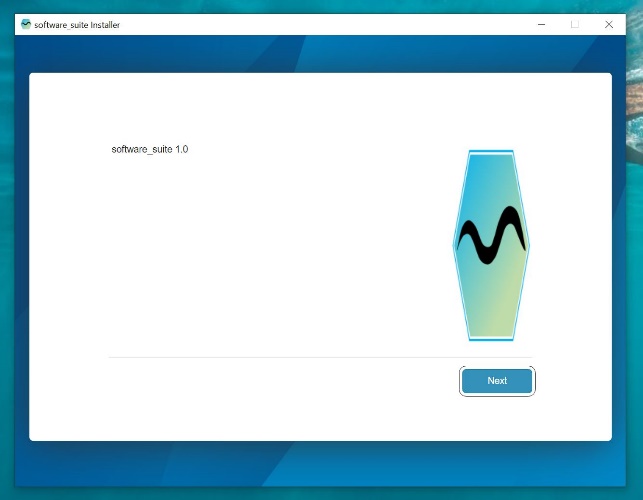


1. Select a destination folder for the installation and check the box to add a desktop shortcut.


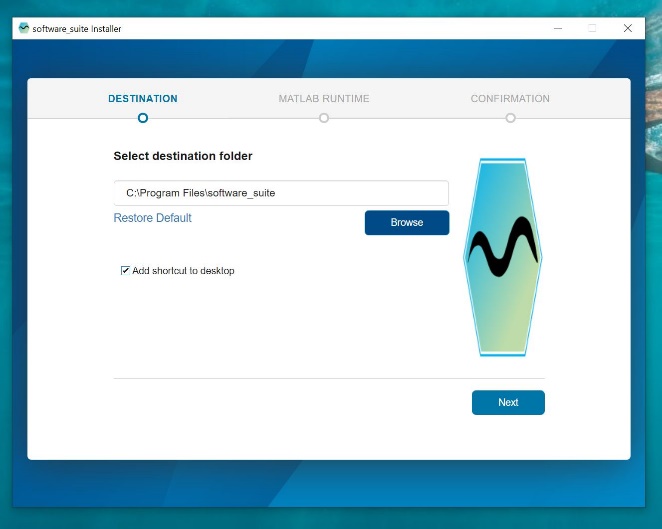


1. Accept the license agreement for MATLAB Runtime.


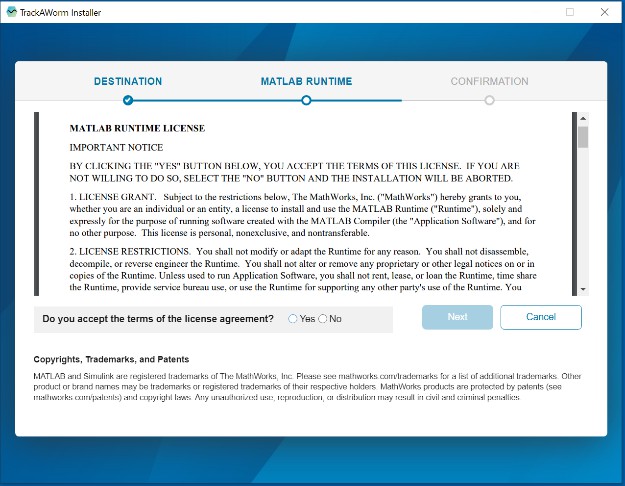


1. Confirm the destination folders for software_suite and MATLAB Runtime, then begin the installation.


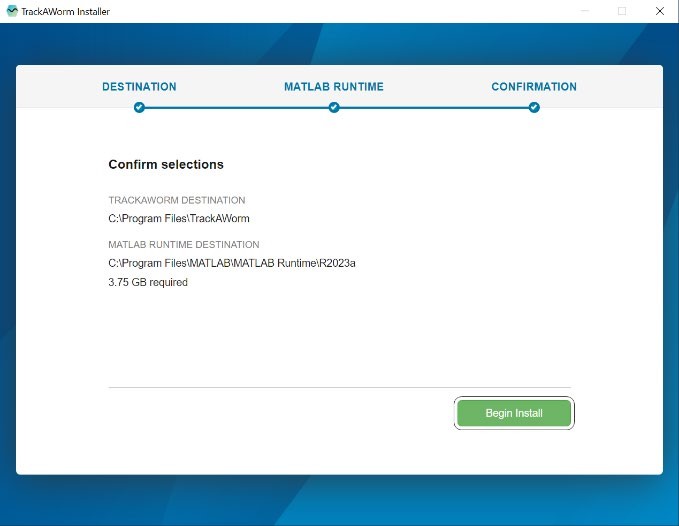


1. Wait until the “Installation Complete” window appears.


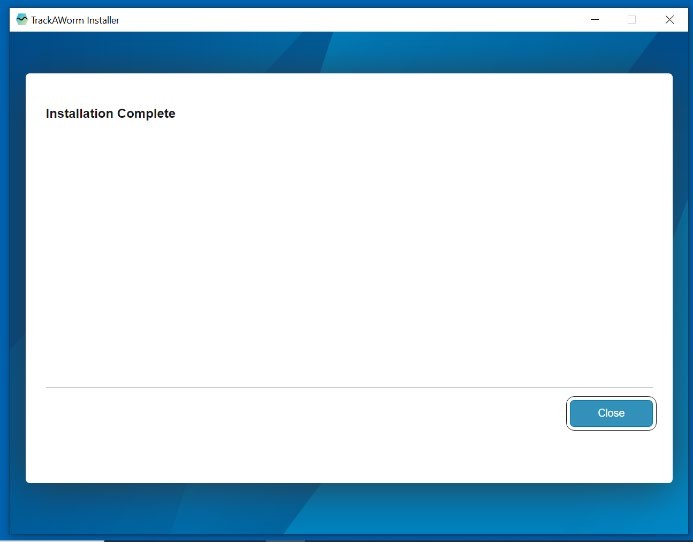


# Camera driver installation

- 1. Download the current version of Vimba (for the CMOS camera) from the manufacturer’s website: <https://www.alliedvision.com/en/products/vimba-sdk/>
  2. Run the Vimba Driver installer, select the option 3rd Party Applications, and proceed with the installation.


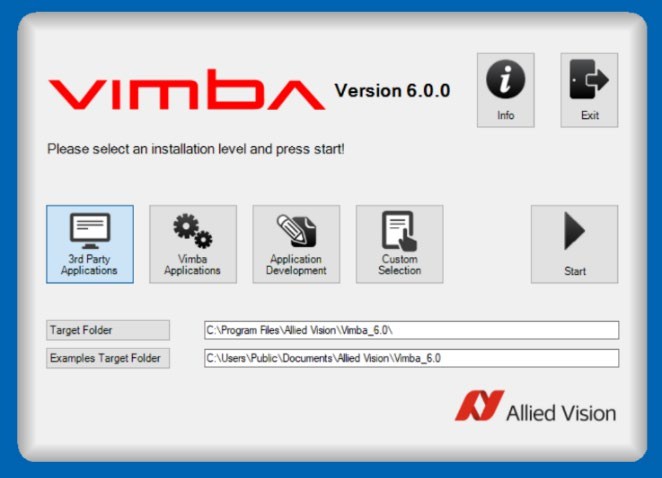


- 1. Select the Transport Layer Interface (e.g. MATLAB) and click OK.


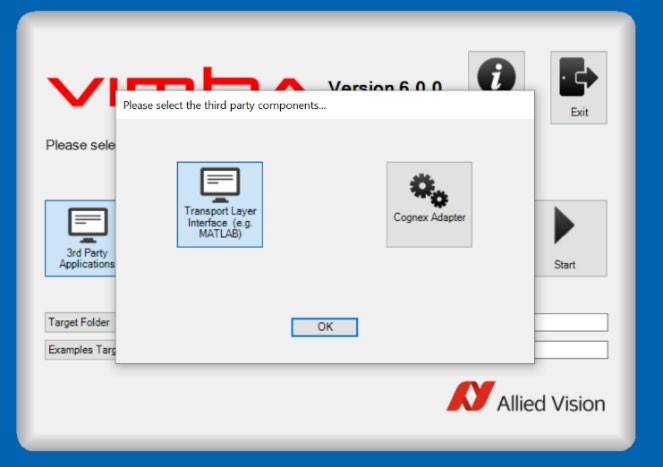


- 1. Confirm the installation is complete and exit the installer.


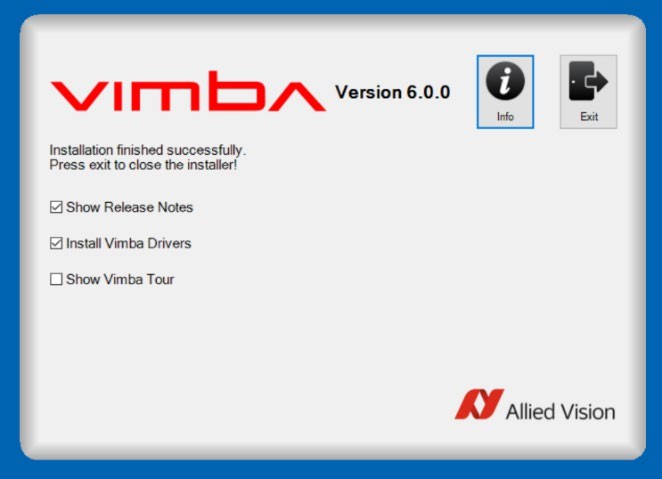


- 1. If you want to install the Vimba Viewer as well, restart the Vimba installer, click on Vimba Applications, and select Camera Demonstration.


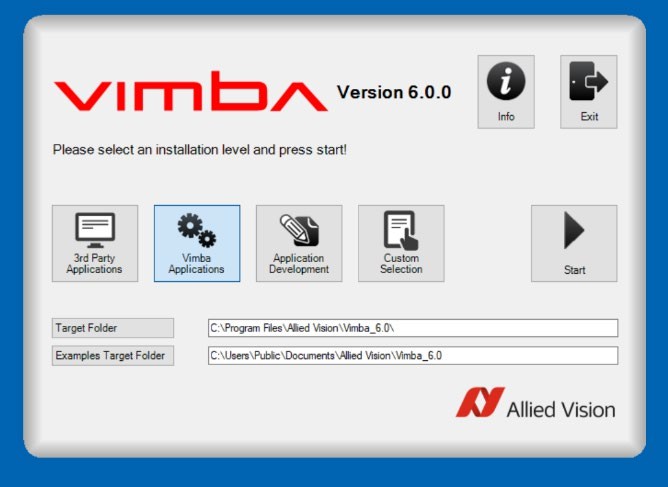


- 1. Open Vimba Viewer to confirm your camera is detected.


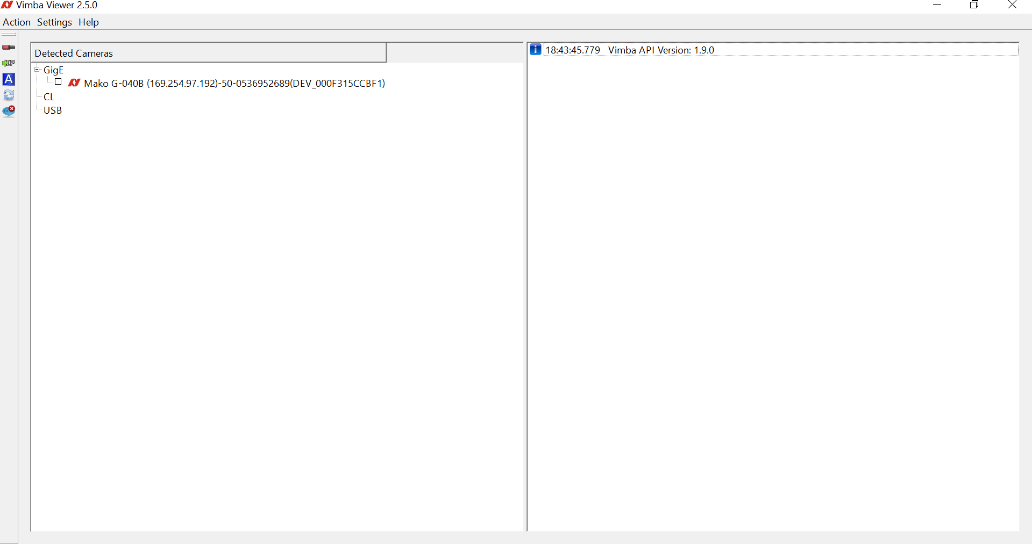


- 1. Select the camera and click Play to confirm the camera is functioning properly.


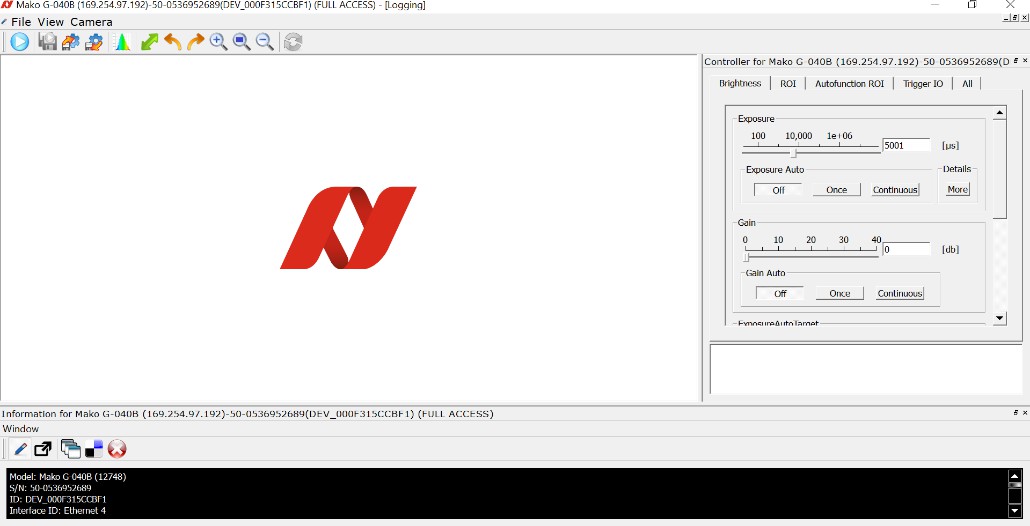


# Stage driver and application software installation

1. Download the “64 Bit Prior Scientific DLL SDK – for installation of Prior terminal and Prior demo programs” (v8.6.5) from the manufacturer’s website: <https://www.prior.com/download-category/software>
2. Run the downloaded file.


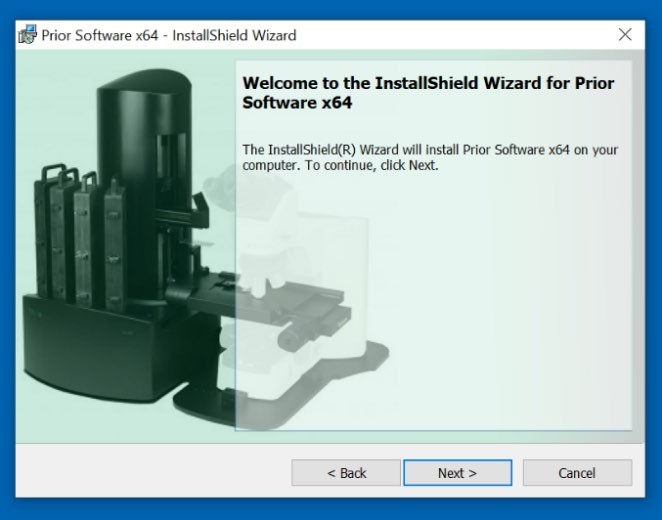


1. Select the destination folder and begin the installation.


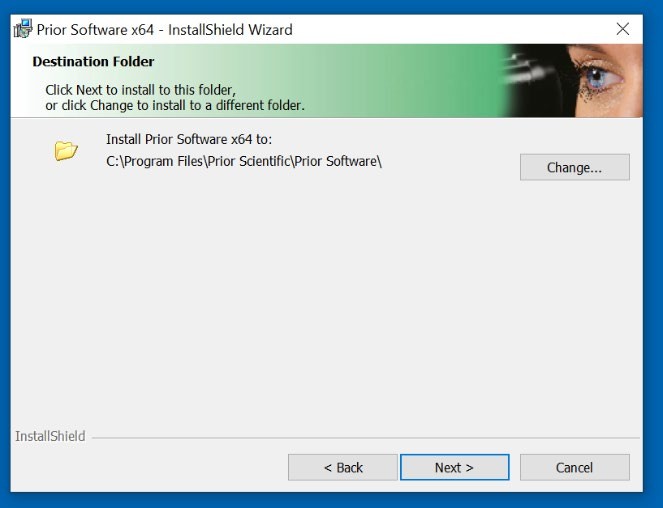


1. Confirm the installation is complete.


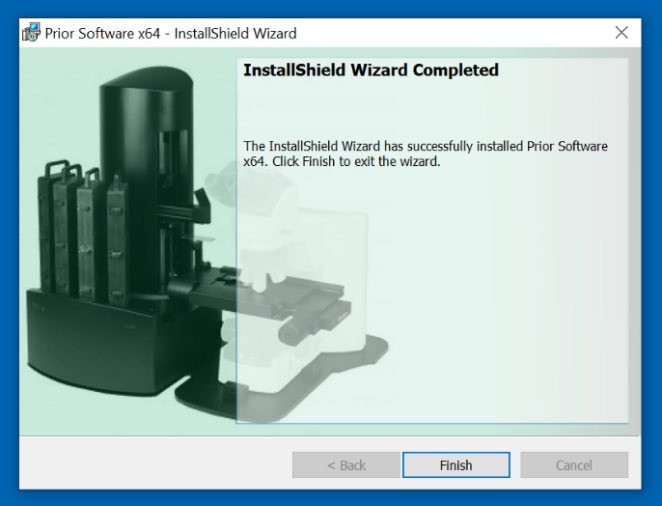


# Stage Functionality Verification and COM Number Identification

Before using the stage, run the Prior Terminal application (installed from the Prior software package) to verify stage functionality and identify the assigned COM number with the following steps.

1. Run the Terminal application.
2. Check the bottom left for the connected COM number and make a note of it. This COM number is needed to enter into the Record module of *WormTracker*.
3. Type “?” and press Enter to see the following:

Optiscan information Drive chips 11111 Joystick not fitted Stage = ES111/1 Focus = None Filter_1 = None Filter_2 = None Shutters = 000

End

1. Test the stage movement by typing each command and pressing Enter: “l,10000”, “r,10000”, “b,10000”, and “f,10000”. The stage should move left, right, backward, and forward, respectively. Each movement covers 1.0 cm when the stage resolution is set to 1.0 µm.

**Manual Re-Centering of the Motorized Stage**

By default, the stage controller knows the center of the stage. However, in extremely rare cases, this information can be lost. As a result, you may not be able to properly center the stage in the *WormTracker*. Follow the steps below to re-center the stage:

1. Run the Terminal application.
2. Type “g,**10000**,**10000**” and press Enter to observe the stage move to the extreme ends of *X* and *Y*.
3. Press Enter again to observe two numbers (e.g. 63509,78591).
4. Type “g,**-10000**,**-10000**” to observe the stage move to the OPPOSITE extreme ends.
5. Press Enter again to observe two negative numbers (e.g. -63189, -47486).
6. Type “gr,63349,63038”, which is ½ of the sum of the two numbers (use absolute numbers) in step 2 and 4 [e. g. (63509+63189)/2 = 63349; (78591+47486)/2 = 63038] and press Enter to observe the stage move to the center.
7. Type “z” and press Enter to save the stage center.
8. Type “m” and press Enter to recall the center.

The numbers in bold are for a stage resolution of 1.0 µm. You must increase them by 10 and 100 times for stage resolutions of 0.1 µm and 0.01 µm, respectively. The higher resolutions are not needed for worm tracking.

The stage controller remembers the new stage center.

# Changing Stage Resolution

1. Run the Terminal application.
2. Type “res,s” and press Enter to see the current stage resolution. It can be 1, 0.1, or 0.01 µm.
3. To change the resolution to 1 µm, type “res,s,1” and press Enter.
4. You can confirm the change by repeating step 1.

**If you have any questions about the stage, you should contact Prior Scientific. Their technical support is easily accessible by phone.**
