# Supplementary figures and images for: Track-A-Worm 2.0: A Software Suite for Quantifying Properties of C. elegans Locomotion, Bending, Sleep, and Action Potentials (part 3 of 3)
Source: eNeuro. 2025 Aug 13;12(8):ENEURO.0224-25.2025. doi: 10.1523/ENEURO.0224-25.2025 (PMC12393025; doi:10.1523/ENEURO.0224-25.2025)

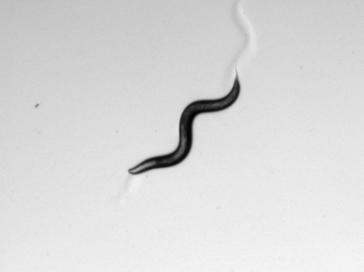

Supplement: Extended Data 4 — A sample WormTracker recording. This folder contains the recording of a wild-type worm (60 seconds, 15 frames per second), along with the associated stage file, time file, and a spline file generated by the Fit Spline module. The images were captured at 50% of the camera's resolution (4 KB/image). Download Extended Data 4, ZIP file. [file eneuro-12-ENEURO.0224-25.2025-s006.zip › Extended Data 4/wt1/L_img00184.jpeg]

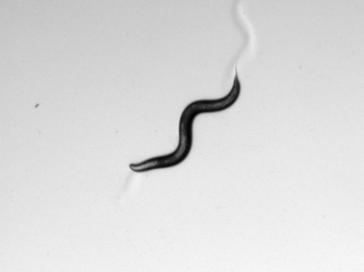

Supplement: Extended Data 4 — A sample WormTracker recording. This folder contains the recording of a wild-type worm (60 seconds, 15 frames per second), along with the associated stage file, time file, and a spline file generated by the Fit Spline module. The images were captured at 50% of the camera's resolution (4 KB/image). Download Extended Data 4, ZIP file. [file eneuro-12-ENEURO.0224-25.2025-s006.zip › Extended Data 4/wt1/L_img00185.jpeg]

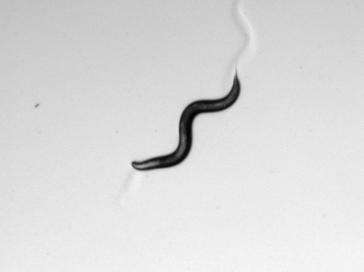

Supplement: Extended Data 4 — A sample WormTracker recording. This folder contains the recording of a wild-type worm (60 seconds, 15 frames per second), along with the associated stage file, time file, and a spline file generated by the Fit Spline module. The images were captured at 50% of the camera's resolution (4 KB/image). Download Extended Data 4, ZIP file. [file eneuro-12-ENEURO.0224-25.2025-s006.zip › Extended Data 4/wt1/L_img00186.jpeg]

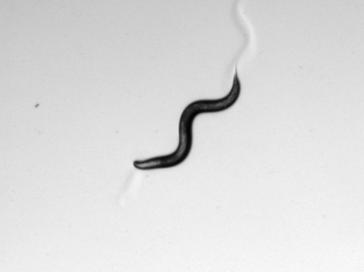

Supplement: Extended Data 4 — A sample WormTracker recording. This folder contains the recording of a wild-type worm (60 seconds, 15 frames per second), along with the associated stage file, time file, and a spline file generated by the Fit Spline module. The images were captured at 50% of the camera's resolution (4 KB/image). Download Extended Data 4, ZIP file. [file eneuro-12-ENEURO.0224-25.2025-s006.zip › Extended Data 4/wt1/L_img00187.jpeg]

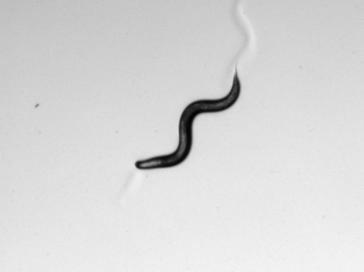

Supplement: Extended Data 4 — A sample WormTracker recording. This folder contains the recording of a wild-type worm (60 seconds, 15 frames per second), along with the associated stage file, time file, and a spline file generated by the Fit Spline module. The images were captured at 50% of the camera's resolution (4 KB/image). Download Extended Data 4, ZIP file. [file eneuro-12-ENEURO.0224-25.2025-s006.zip › Extended Data 4/wt1/L_img00188.jpeg]

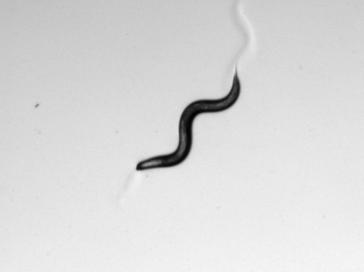

Supplement: Extended Data 4 — A sample WormTracker recording. This folder contains the recording of a wild-type worm (60 seconds, 15 frames per second), along with the associated stage file, time file, and a spline file generated by the Fit Spline module. The images were captured at 50% of the camera's resolution (4 KB/image). Download Extended Data 4, ZIP file. [file eneuro-12-ENEURO.0224-25.2025-s006.zip › Extended Data 4/wt1/L_img00189.jpeg]

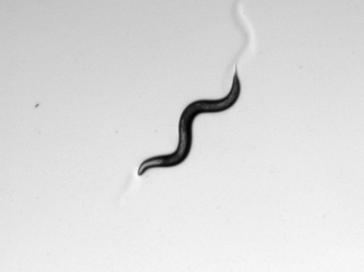

Supplement: Extended Data 4 — A sample WormTracker recording. This folder contains the recording of a wild-type worm (60 seconds, 15 frames per second), along with the associated stage file, time file, and a spline file generated by the Fit Spline module. The images were captured at 50% of the camera's resolution (4 KB/image). Download Extended Data 4, ZIP file. [file eneuro-12-ENEURO.0224-25.2025-s006.zip › Extended Data 4/wt1/L_img00190.jpeg]

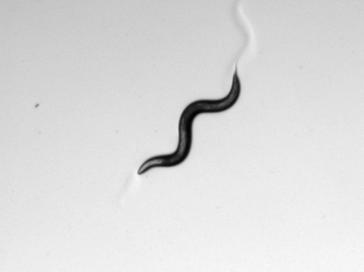

Supplement: Extended Data 4 — A sample WormTracker recording. This folder contains the recording of a wild-type worm (60 seconds, 15 frames per second), along with the associated stage file, time file, and a spline file generated by the Fit Spline module. The images were captured at 50% of the camera's resolution (4 KB/image). Download Extended Data 4, ZIP file. [file eneuro-12-ENEURO.0224-25.2025-s006.zip › Extended Data 4/wt1/L_img00191.jpeg]

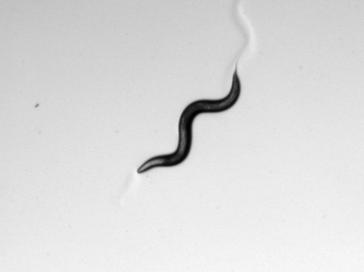

Supplement: Extended Data 4 — A sample WormTracker recording. This folder contains the recording of a wild-type worm (60 seconds, 15 frames per second), along with the associated stage file, time file, and a spline file generated by the Fit Spline module. The images were captured at 50% of the camera's resolution (4 KB/image). Download Extended Data 4, ZIP file. [file eneuro-12-ENEURO.0224-25.2025-s006.zip › Extended Data 4/wt1/L_img00192.jpeg]

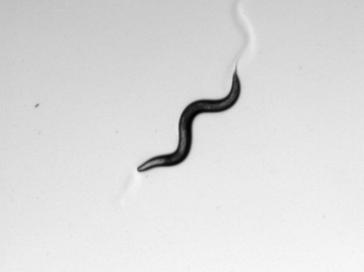

Supplement: Extended Data 4 — A sample WormTracker recording. This folder contains the recording of a wild-type worm (60 seconds, 15 frames per second), along with the associated stage file, time file, and a spline file generated by the Fit Spline module. The images were captured at 50% of the camera's resolution (4 KB/image). Download Extended Data 4, ZIP file. [file eneuro-12-ENEURO.0224-25.2025-s006.zip › Extended Data 4/wt1/L_img00193.jpeg]

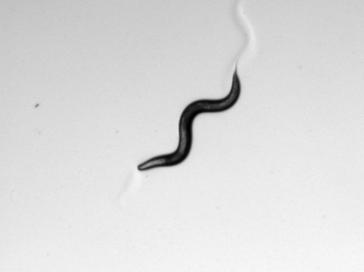

Supplement: Extended Data 4 — A sample WormTracker recording. This folder contains the recording of a wild-type worm (60 seconds, 15 frames per second), along with the associated stage file, time file, and a spline file generated by the Fit Spline module. The images were captured at 50% of the camera's resolution (4 KB/image). Download Extended Data 4, ZIP file. [file eneuro-12-ENEURO.0224-25.2025-s006.zip › Extended Data 4/wt1/L_img00194.jpeg]

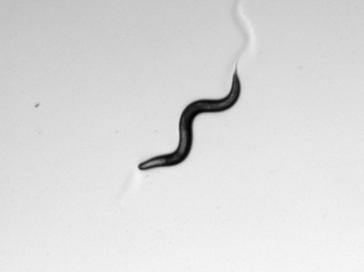

Supplement: Extended Data 4 — A sample WormTracker recording. This folder contains the recording of a wild-type worm (60 seconds, 15 frames per second), along with the associated stage file, time file, and a spline file generated by the Fit Spline module. The images were captured at 50% of the camera's resolution (4 KB/image). Download Extended Data 4, ZIP file. [file eneuro-12-ENEURO.0224-25.2025-s006.zip › Extended Data 4/wt1/L_img00195.jpeg]

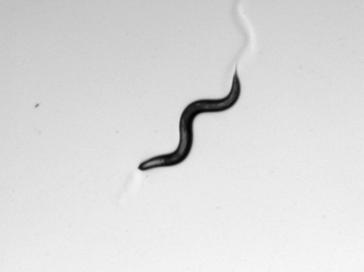

Supplement: Extended Data 4 — A sample WormTracker recording. This folder contains the recording of a wild-type worm (60 seconds, 15 frames per second), along with the associated stage file, time file, and a spline file generated by the Fit Spline module. The images were captured at 50% of the camera's resolution (4 KB/image). Download Extended Data 4, ZIP file. [file eneuro-12-ENEURO.0224-25.2025-s006.zip › Extended Data 4/wt1/L_img00196.jpeg]

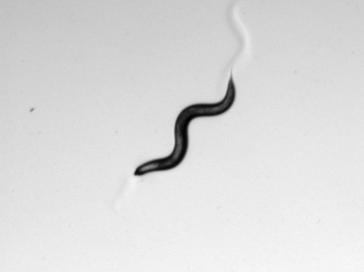

Supplement: Extended Data 4 — A sample WormTracker recording. This folder contains the recording of a wild-type worm (60 seconds, 15 frames per second), along with the associated stage file, time file, and a spline file generated by the Fit Spline module. The images were captured at 50% of the camera's resolution (4 KB/image). Download Extended Data 4, ZIP file. [file eneuro-12-ENEURO.0224-25.2025-s006.zip › Extended Data 4/wt1/L_img00197.jpeg]

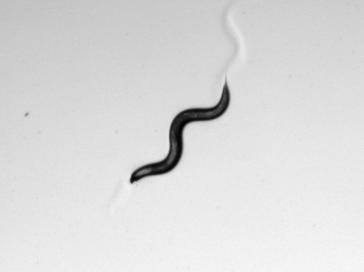

Supplement: Extended Data 4 — A sample WormTracker recording. This folder contains the recording of a wild-type worm (60 seconds, 15 frames per second), along with the associated stage file, time file, and a spline file generated by the Fit Spline module. The images were captured at 50% of the camera's resolution (4 KB/image). Download Extended Data 4, ZIP file. [file eneuro-12-ENEURO.0224-25.2025-s006.zip › Extended Data 4/wt1/L_img00198.jpeg]

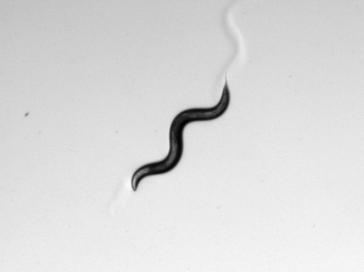

Supplement: Extended Data 4 — A sample WormTracker recording. This folder contains the recording of a wild-type worm (60 seconds, 15 frames per second), along with the associated stage file, time file, and a spline file generated by the Fit Spline module. The images were captured at 50% of the camera's resolution (4 KB/image). Download Extended Data 4, ZIP file. [file eneuro-12-ENEURO.0224-25.2025-s006.zip › Extended Data 4/wt1/L_img00199.jpeg]

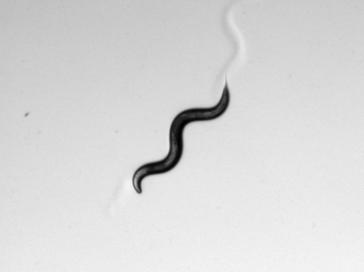

Supplement: Extended Data 4 — A sample WormTracker recording. This folder contains the recording of a wild-type worm (60 seconds, 15 frames per second), along with the associated stage file, time file, and a spline file generated by the Fit Spline module. The images were captured at 50% of the camera's resolution (4 KB/image). Download Extended Data 4, ZIP file. [file eneuro-12-ENEURO.0224-25.2025-s006.zip › Extended Data 4/wt1/L_img00200.jpeg]

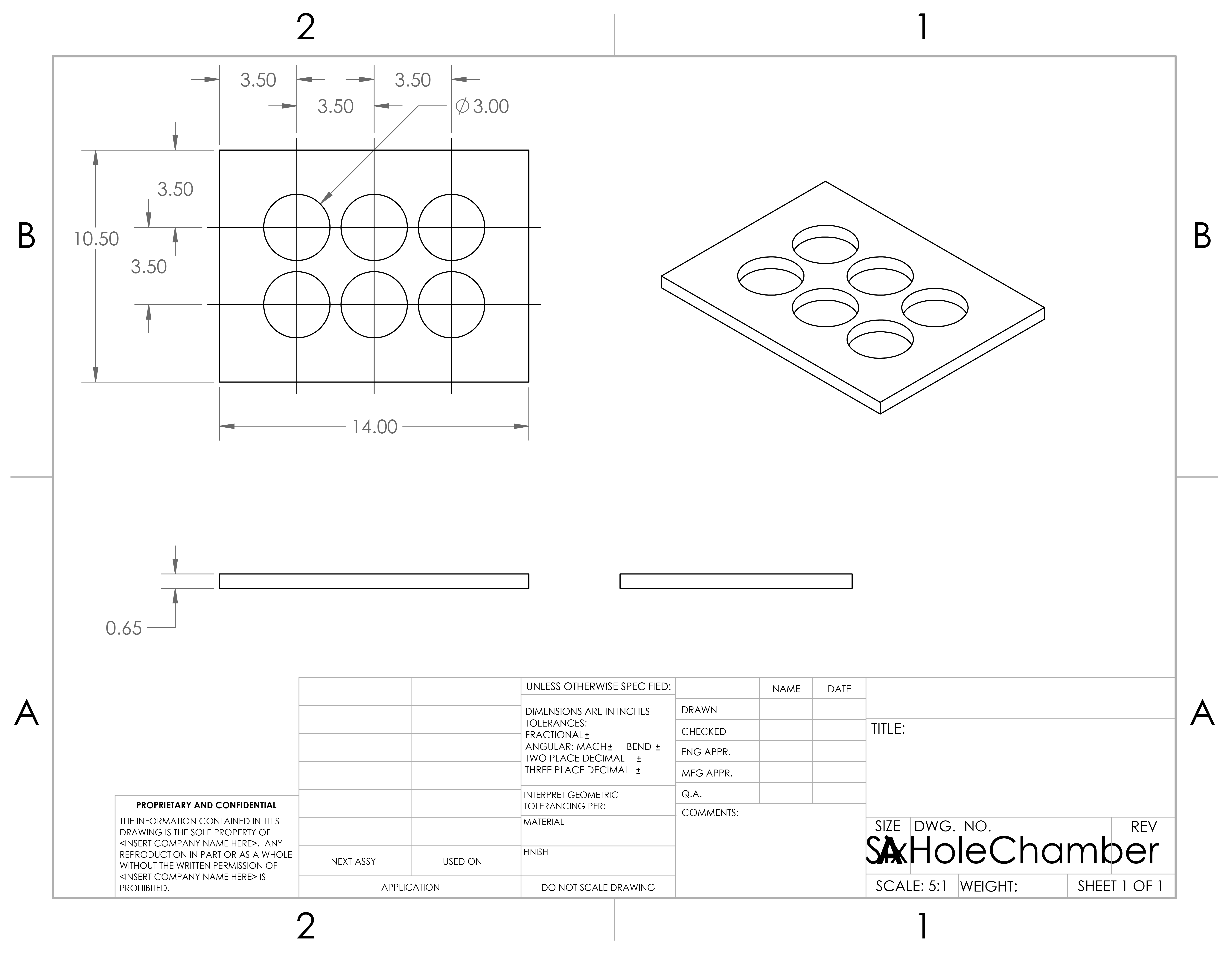

Supplement: Figure 4-1 — CAD design of the polydimethylsiloxane (PDMS) sleep recording chamber. This schematic illustrates the custom-fabricated PDMS membrane used to isolate individual C. elegans during sleep behavior recordings. The membrane features six uniformly spaced circular wells (3.0 mm diameter) embedded in a rectangular frame (14.0 mm × 10.5 mm × 0.65 mm). Download Figure 4-1, TIF file. [file eneuro-12-ENEURO.0224-25.2025-s002.tif]
