# Supplementary figures and images for: Track-A-Worm 2.0: A Software Suite for Quantifying Properties of C. elegans Locomotion, Bending, Sleep, and Action Potentials (part 2 of 3)
Source: eNeuro. 2025 Aug 13;12(8):ENEURO.0224-25.2025. doi: 10.1523/ENEURO.0224-25.2025 (PMC12393025; doi:10.1523/ENEURO.0224-25.2025)

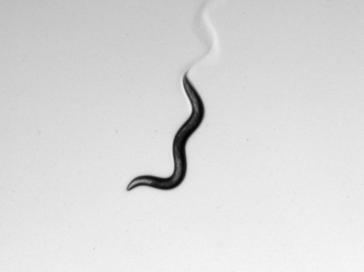

Supplement: Extended Data 4 — A sample WormTracker recording. This folder contains the recording of a wild-type worm (60 seconds, 15 frames per second), along with the associated stage file, time file, and a spline file generated by the Fit Spline module. The images were captured at 50% of the camera's resolution (4 KB/image). Download Extended Data 4, ZIP file. [file eneuro-12-ENEURO.0224-25.2025-s006.zip › Extended Data 4/wt1/L_img00084.jpeg]

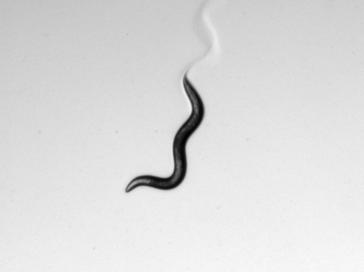

Supplement: Extended Data 4 — A sample WormTracker recording. This folder contains the recording of a wild-type worm (60 seconds, 15 frames per second), along with the associated stage file, time file, and a spline file generated by the Fit Spline module. The images were captured at 50% of the camera's resolution (4 KB/image). Download Extended Data 4, ZIP file. [file eneuro-12-ENEURO.0224-25.2025-s006.zip › Extended Data 4/wt1/L_img00085.jpeg]

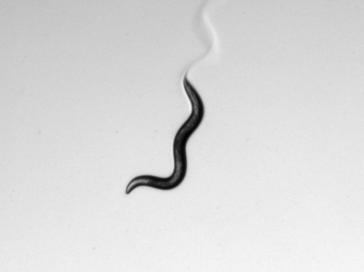

Supplement: Extended Data 4 — A sample WormTracker recording. This folder contains the recording of a wild-type worm (60 seconds, 15 frames per second), along with the associated stage file, time file, and a spline file generated by the Fit Spline module. The images were captured at 50% of the camera's resolution (4 KB/image). Download Extended Data 4, ZIP file. [file eneuro-12-ENEURO.0224-25.2025-s006.zip › Extended Data 4/wt1/L_img00086.jpeg]

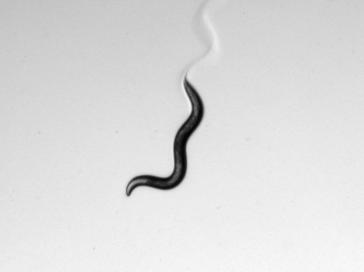

Supplement: Extended Data 4 — A sample WormTracker recording. This folder contains the recording of a wild-type worm (60 seconds, 15 frames per second), along with the associated stage file, time file, and a spline file generated by the Fit Spline module. The images were captured at 50% of the camera's resolution (4 KB/image). Download Extended Data 4, ZIP file. [file eneuro-12-ENEURO.0224-25.2025-s006.zip › Extended Data 4/wt1/L_img00087.jpeg]

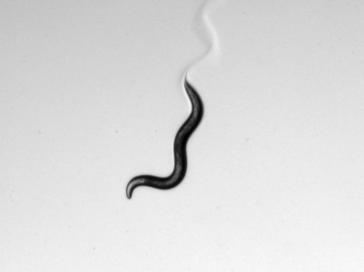

Supplement: Extended Data 4 — A sample WormTracker recording. This folder contains the recording of a wild-type worm (60 seconds, 15 frames per second), along with the associated stage file, time file, and a spline file generated by the Fit Spline module. The images were captured at 50% of the camera's resolution (4 KB/image). Download Extended Data 4, ZIP file. [file eneuro-12-ENEURO.0224-25.2025-s006.zip › Extended Data 4/wt1/L_img00088.jpeg]

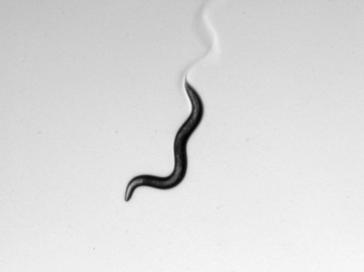

Supplement: Extended Data 4 — A sample WormTracker recording. This folder contains the recording of a wild-type worm (60 seconds, 15 frames per second), along with the associated stage file, time file, and a spline file generated by the Fit Spline module. The images were captured at 50% of the camera's resolution (4 KB/image). Download Extended Data 4, ZIP file. [file eneuro-12-ENEURO.0224-25.2025-s006.zip › Extended Data 4/wt1/L_img00089.jpeg]

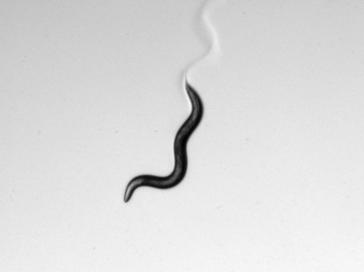

Supplement: Extended Data 4 — A sample WormTracker recording. This folder contains the recording of a wild-type worm (60 seconds, 15 frames per second), along with the associated stage file, time file, and a spline file generated by the Fit Spline module. The images were captured at 50% of the camera's resolution (4 KB/image). Download Extended Data 4, ZIP file. [file eneuro-12-ENEURO.0224-25.2025-s006.zip › Extended Data 4/wt1/L_img00090.jpeg]

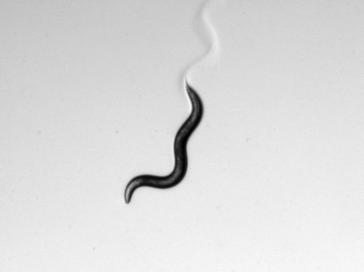

Supplement: Extended Data 4 — A sample WormTracker recording. This folder contains the recording of a wild-type worm (60 seconds, 15 frames per second), along with the associated stage file, time file, and a spline file generated by the Fit Spline module. The images were captured at 50% of the camera's resolution (4 KB/image). Download Extended Data 4, ZIP file. [file eneuro-12-ENEURO.0224-25.2025-s006.zip › Extended Data 4/wt1/L_img00091.jpeg]

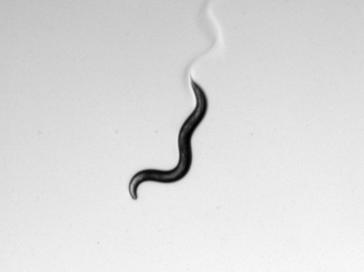

Supplement: Extended Data 4 — A sample WormTracker recording. This folder contains the recording of a wild-type worm (60 seconds, 15 frames per second), along with the associated stage file, time file, and a spline file generated by the Fit Spline module. The images were captured at 50% of the camera's resolution (4 KB/image). Download Extended Data 4, ZIP file. [file eneuro-12-ENEURO.0224-25.2025-s006.zip › Extended Data 4/wt1/L_img00092.jpeg]

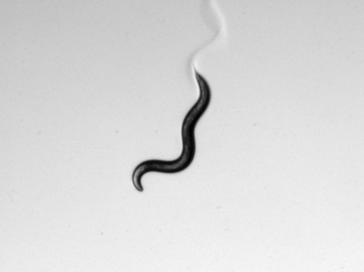

Supplement: Extended Data 4 — A sample WormTracker recording. This folder contains the recording of a wild-type worm (60 seconds, 15 frames per second), along with the associated stage file, time file, and a spline file generated by the Fit Spline module. The images were captured at 50% of the camera's resolution (4 KB/image). Download Extended Data 4, ZIP file. [file eneuro-12-ENEURO.0224-25.2025-s006.zip › Extended Data 4/wt1/L_img00093.jpeg]

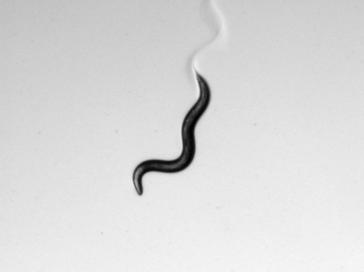

Supplement: Extended Data 4 — A sample WormTracker recording. This folder contains the recording of a wild-type worm (60 seconds, 15 frames per second), along with the associated stage file, time file, and a spline file generated by the Fit Spline module. The images were captured at 50% of the camera's resolution (4 KB/image). Download Extended Data 4, ZIP file. [file eneuro-12-ENEURO.0224-25.2025-s006.zip › Extended Data 4/wt1/L_img00094.jpeg]

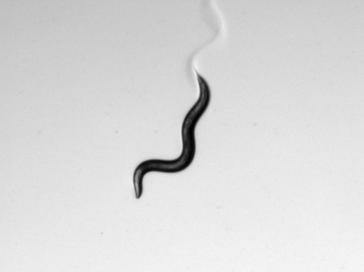

Supplement: Extended Data 4 — A sample WormTracker recording. This folder contains the recording of a wild-type worm (60 seconds, 15 frames per second), along with the associated stage file, time file, and a spline file generated by the Fit Spline module. The images were captured at 50% of the camera's resolution (4 KB/image). Download Extended Data 4, ZIP file. [file eneuro-12-ENEURO.0224-25.2025-s006.zip › Extended Data 4/wt1/L_img00095.jpeg]

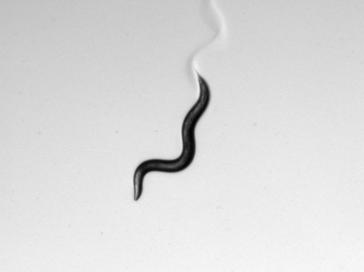

Supplement: Extended Data 4 — A sample WormTracker recording. This folder contains the recording of a wild-type worm (60 seconds, 15 frames per second), along with the associated stage file, time file, and a spline file generated by the Fit Spline module. The images were captured at 50% of the camera's resolution (4 KB/image). Download Extended Data 4, ZIP file. [file eneuro-12-ENEURO.0224-25.2025-s006.zip › Extended Data 4/wt1/L_img00096.jpeg]

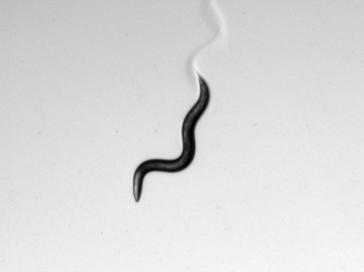

Supplement: Extended Data 4 — A sample WormTracker recording. This folder contains the recording of a wild-type worm (60 seconds, 15 frames per second), along with the associated stage file, time file, and a spline file generated by the Fit Spline module. The images were captured at 50% of the camera's resolution (4 KB/image). Download Extended Data 4, ZIP file. [file eneuro-12-ENEURO.0224-25.2025-s006.zip › Extended Data 4/wt1/L_img00097.jpeg]

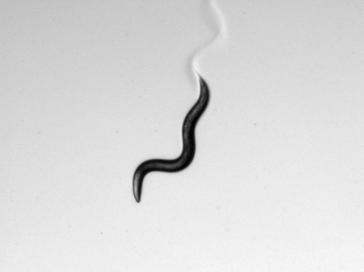

Supplement: Extended Data 4 — A sample WormTracker recording. This folder contains the recording of a wild-type worm (60 seconds, 15 frames per second), along with the associated stage file, time file, and a spline file generated by the Fit Spline module. The images were captured at 50% of the camera's resolution (4 KB/image). Download Extended Data 4, ZIP file. [file eneuro-12-ENEURO.0224-25.2025-s006.zip › Extended Data 4/wt1/L_img00098.jpeg]

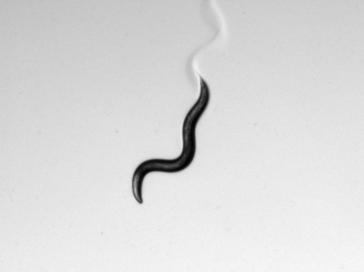

Supplement: Extended Data 4 — A sample WormTracker recording. This folder contains the recording of a wild-type worm (60 seconds, 15 frames per second), along with the associated stage file, time file, and a spline file generated by the Fit Spline module. The images were captured at 50% of the camera's resolution (4 KB/image). Download Extended Data 4, ZIP file. [file eneuro-12-ENEURO.0224-25.2025-s006.zip › Extended Data 4/wt1/L_img00099.jpeg]

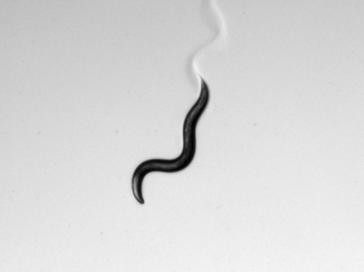

Supplement: Extended Data 4 — A sample WormTracker recording. This folder contains the recording of a wild-type worm (60 seconds, 15 frames per second), along with the associated stage file, time file, and a spline file generated by the Fit Spline module. The images were captured at 50% of the camera's resolution (4 KB/image). Download Extended Data 4, ZIP file. [file eneuro-12-ENEURO.0224-25.2025-s006.zip › Extended Data 4/wt1/L_img00100.jpeg]

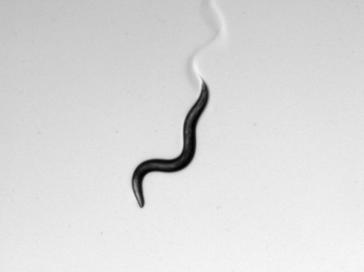

Supplement: Extended Data 4 — A sample WormTracker recording. This folder contains the recording of a wild-type worm (60 seconds, 15 frames per second), along with the associated stage file, time file, and a spline file generated by the Fit Spline module. The images were captured at 50% of the camera's resolution (4 KB/image). Download Extended Data 4, ZIP file. [file eneuro-12-ENEURO.0224-25.2025-s006.zip › Extended Data 4/wt1/L_img00101.jpeg]

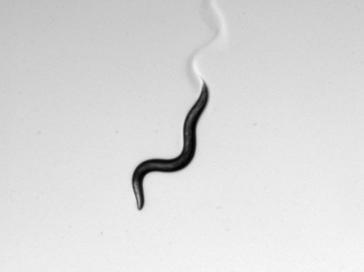

Supplement: Extended Data 4 — A sample WormTracker recording. This folder contains the recording of a wild-type worm (60 seconds, 15 frames per second), along with the associated stage file, time file, and a spline file generated by the Fit Spline module. The images were captured at 50% of the camera's resolution (4 KB/image). Download Extended Data 4, ZIP file. [file eneuro-12-ENEURO.0224-25.2025-s006.zip › Extended Data 4/wt1/L_img00102.jpeg]

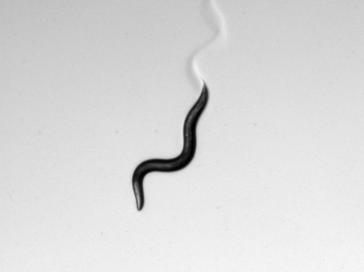

Supplement: Extended Data 4 — A sample WormTracker recording. This folder contains the recording of a wild-type worm (60 seconds, 15 frames per second), along with the associated stage file, time file, and a spline file generated by the Fit Spline module. The images were captured at 50% of the camera's resolution (4 KB/image). Download Extended Data 4, ZIP file. [file eneuro-12-ENEURO.0224-25.2025-s006.zip › Extended Data 4/wt1/L_img00103.jpeg]

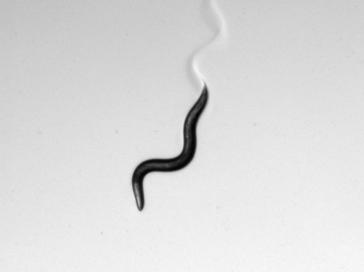

Supplement: Extended Data 4 — A sample WormTracker recording. This folder contains the recording of a wild-type worm (60 seconds, 15 frames per second), along with the associated stage file, time file, and a spline file generated by the Fit Spline module. The images were captured at 50% of the camera's resolution (4 KB/image). Download Extended Data 4, ZIP file. [file eneuro-12-ENEURO.0224-25.2025-s006.zip › Extended Data 4/wt1/L_img00104.jpeg]

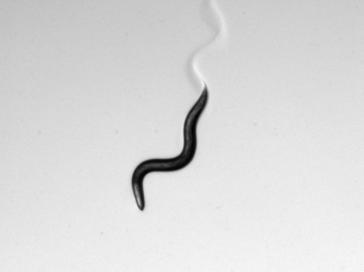

Supplement: Extended Data 4 — A sample WormTracker recording. This folder contains the recording of a wild-type worm (60 seconds, 15 frames per second), along with the associated stage file, time file, and a spline file generated by the Fit Spline module. The images were captured at 50% of the camera's resolution (4 KB/image). Download Extended Data 4, ZIP file. [file eneuro-12-ENEURO.0224-25.2025-s006.zip › Extended Data 4/wt1/L_img00105.jpeg]

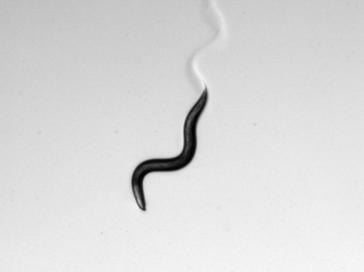

Supplement: Extended Data 4 — A sample WormTracker recording. This folder contains the recording of a wild-type worm (60 seconds, 15 frames per second), along with the associated stage file, time file, and a spline file generated by the Fit Spline module. The images were captured at 50% of the camera's resolution (4 KB/image). Download Extended Data 4, ZIP file. [file eneuro-12-ENEURO.0224-25.2025-s006.zip › Extended Data 4/wt1/L_img00106.jpeg]

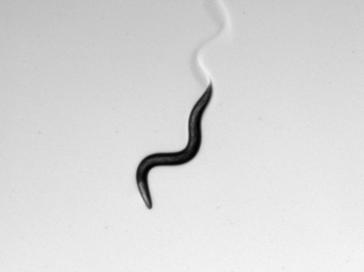

Supplement: Extended Data 4 — A sample WormTracker recording. This folder contains the recording of a wild-type worm (60 seconds, 15 frames per second), along with the associated stage file, time file, and a spline file generated by the Fit Spline module. The images were captured at 50% of the camera's resolution (4 KB/image). Download Extended Data 4, ZIP file. [file eneuro-12-ENEURO.0224-25.2025-s006.zip › Extended Data 4/wt1/L_img00107.jpeg]

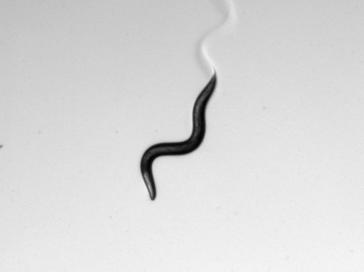

Supplement: Extended Data 4 — A sample WormTracker recording. This folder contains the recording of a wild-type worm (60 seconds, 15 frames per second), along with the associated stage file, time file, and a spline file generated by the Fit Spline module. The images were captured at 50% of the camera's resolution (4 KB/image). Download Extended Data 4, ZIP file. [file eneuro-12-ENEURO.0224-25.2025-s006.zip › Extended Data 4/wt1/L_img00108.jpeg]

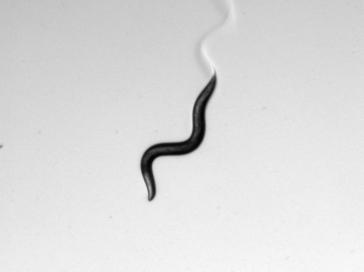

Supplement: Extended Data 4 — A sample WormTracker recording. This folder contains the recording of a wild-type worm (60 seconds, 15 frames per second), along with the associated stage file, time file, and a spline file generated by the Fit Spline module. The images were captured at 50% of the camera's resolution (4 KB/image). Download Extended Data 4, ZIP file. [file eneuro-12-ENEURO.0224-25.2025-s006.zip › Extended Data 4/wt1/L_img00109.jpeg]

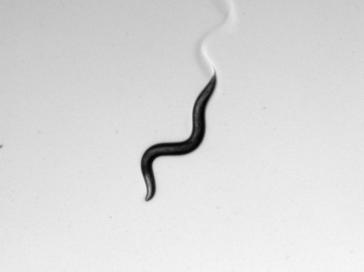

Supplement: Extended Data 4 — A sample WormTracker recording. This folder contains the recording of a wild-type worm (60 seconds, 15 frames per second), along with the associated stage file, time file, and a spline file generated by the Fit Spline module. The images were captured at 50% of the camera's resolution (4 KB/image). Download Extended Data 4, ZIP file. [file eneuro-12-ENEURO.0224-25.2025-s006.zip › Extended Data 4/wt1/L_img00110.jpeg]

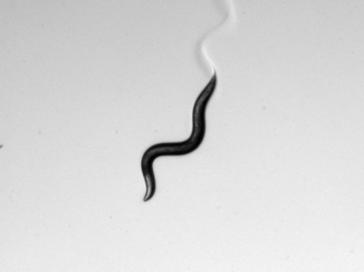

Supplement: Extended Data 4 — A sample WormTracker recording. This folder contains the recording of a wild-type worm (60 seconds, 15 frames per second), along with the associated stage file, time file, and a spline file generated by the Fit Spline module. The images were captured at 50% of the camera's resolution (4 KB/image). Download Extended Data 4, ZIP file. [file eneuro-12-ENEURO.0224-25.2025-s006.zip › Extended Data 4/wt1/L_img00111.jpeg]

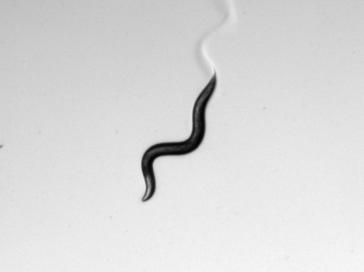

Supplement: Extended Data 4 — A sample WormTracker recording. This folder contains the recording of a wild-type worm (60 seconds, 15 frames per second), along with the associated stage file, time file, and a spline file generated by the Fit Spline module. The images were captured at 50% of the camera's resolution (4 KB/image). Download Extended Data 4, ZIP file. [file eneuro-12-ENEURO.0224-25.2025-s006.zip › Extended Data 4/wt1/L_img00112.jpeg]

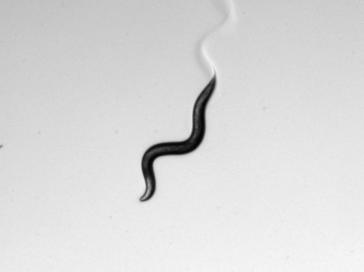

Supplement: Extended Data 4 — A sample WormTracker recording. This folder contains the recording of a wild-type worm (60 seconds, 15 frames per second), along with the associated stage file, time file, and a spline file generated by the Fit Spline module. The images were captured at 50% of the camera's resolution (4 KB/image). Download Extended Data 4, ZIP file. [file eneuro-12-ENEURO.0224-25.2025-s006.zip › Extended Data 4/wt1/L_img00113.jpeg]

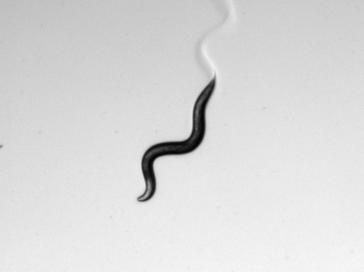

Supplement: Extended Data 4 — A sample WormTracker recording. This folder contains the recording of a wild-type worm (60 seconds, 15 frames per second), along with the associated stage file, time file, and a spline file generated by the Fit Spline module. The images were captured at 50% of the camera's resolution (4 KB/image). Download Extended Data 4, ZIP file. [file eneuro-12-ENEURO.0224-25.2025-s006.zip › Extended Data 4/wt1/L_img00114.jpeg]

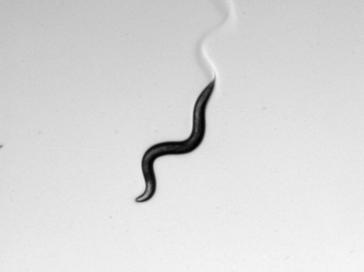

Supplement: Extended Data 4 — A sample WormTracker recording. This folder contains the recording of a wild-type worm (60 seconds, 15 frames per second), along with the associated stage file, time file, and a spline file generated by the Fit Spline module. The images were captured at 50% of the camera's resolution (4 KB/image). Download Extended Data 4, ZIP file. [file eneuro-12-ENEURO.0224-25.2025-s006.zip › Extended Data 4/wt1/L_img00115.jpeg]

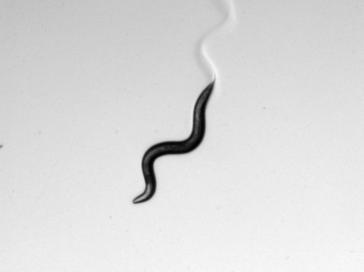

Supplement: Extended Data 4 — A sample WormTracker recording. This folder contains the recording of a wild-type worm (60 seconds, 15 frames per second), along with the associated stage file, time file, and a spline file generated by the Fit Spline module. The images were captured at 50% of the camera's resolution (4 KB/image). Download Extended Data 4, ZIP file. [file eneuro-12-ENEURO.0224-25.2025-s006.zip › Extended Data 4/wt1/L_img00116.jpeg]

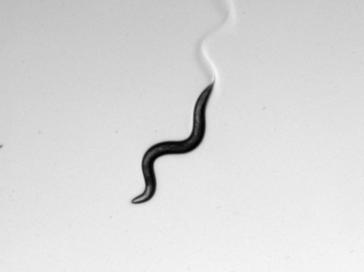

Supplement: Extended Data 4 — A sample WormTracker recording. This folder contains the recording of a wild-type worm (60 seconds, 15 frames per second), along with the associated stage file, time file, and a spline file generated by the Fit Spline module. The images were captured at 50% of the camera's resolution (4 KB/image). Download Extended Data 4, ZIP file. [file eneuro-12-ENEURO.0224-25.2025-s006.zip › Extended Data 4/wt1/L_img00117.jpeg]

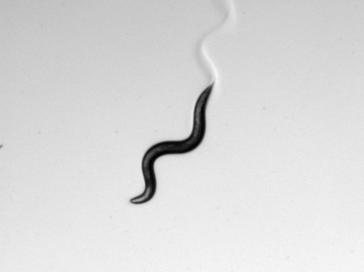

Supplement: Extended Data 4 — A sample WormTracker recording. This folder contains the recording of a wild-type worm (60 seconds, 15 frames per second), along with the associated stage file, time file, and a spline file generated by the Fit Spline module. The images were captured at 50% of the camera's resolution (4 KB/image). Download Extended Data 4, ZIP file. [file eneuro-12-ENEURO.0224-25.2025-s006.zip › Extended Data 4/wt1/L_img00118.jpeg]

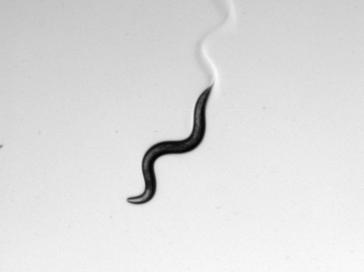

Supplement: Extended Data 4 — A sample WormTracker recording. This folder contains the recording of a wild-type worm (60 seconds, 15 frames per second), along with the associated stage file, time file, and a spline file generated by the Fit Spline module. The images were captured at 50% of the camera's resolution (4 KB/image). Download Extended Data 4, ZIP file. [file eneuro-12-ENEURO.0224-25.2025-s006.zip › Extended Data 4/wt1/L_img00119.jpeg]

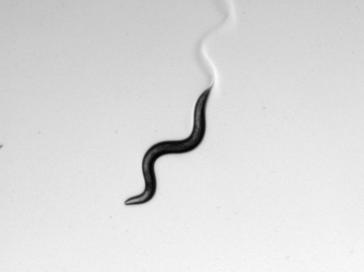

Supplement: Extended Data 4 — A sample WormTracker recording. This folder contains the recording of a wild-type worm (60 seconds, 15 frames per second), along with the associated stage file, time file, and a spline file generated by the Fit Spline module. The images were captured at 50% of the camera's resolution (4 KB/image). Download Extended Data 4, ZIP file. [file eneuro-12-ENEURO.0224-25.2025-s006.zip › Extended Data 4/wt1/L_img00120.jpeg]

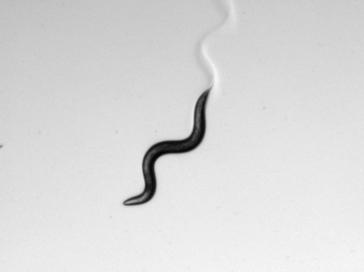

Supplement: Extended Data 4 — A sample WormTracker recording. This folder contains the recording of a wild-type worm (60 seconds, 15 frames per second), along with the associated stage file, time file, and a spline file generated by the Fit Spline module. The images were captured at 50% of the camera's resolution (4 KB/image). Download Extended Data 4, ZIP file. [file eneuro-12-ENEURO.0224-25.2025-s006.zip › Extended Data 4/wt1/L_img00121.jpeg]

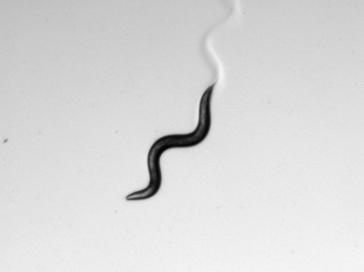

Supplement: Extended Data 4 — A sample WormTracker recording. This folder contains the recording of a wild-type worm (60 seconds, 15 frames per second), along with the associated stage file, time file, and a spline file generated by the Fit Spline module. The images were captured at 50% of the camera's resolution (4 KB/image). Download Extended Data 4, ZIP file. [file eneuro-12-ENEURO.0224-25.2025-s006.zip › Extended Data 4/wt1/L_img00122.jpeg]

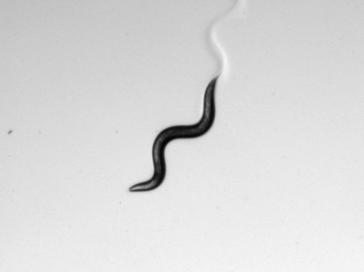

Supplement: Extended Data 4 — A sample WormTracker recording. This folder contains the recording of a wild-type worm (60 seconds, 15 frames per second), along with the associated stage file, time file, and a spline file generated by the Fit Spline module. The images were captured at 50% of the camera's resolution (4 KB/image). Download Extended Data 4, ZIP file. [file eneuro-12-ENEURO.0224-25.2025-s006.zip › Extended Data 4/wt1/L_img00123.jpeg]

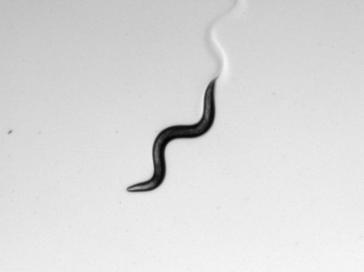

Supplement: Extended Data 4 — A sample WormTracker recording. This folder contains the recording of a wild-type worm (60 seconds, 15 frames per second), along with the associated stage file, time file, and a spline file generated by the Fit Spline module. The images were captured at 50% of the camera's resolution (4 KB/image). Download Extended Data 4, ZIP file. [file eneuro-12-ENEURO.0224-25.2025-s006.zip › Extended Data 4/wt1/L_img00124.jpeg]

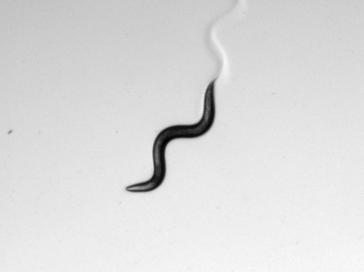

Supplement: Extended Data 4 — A sample WormTracker recording. This folder contains the recording of a wild-type worm (60 seconds, 15 frames per second), along with the associated stage file, time file, and a spline file generated by the Fit Spline module. The images were captured at 50% of the camera's resolution (4 KB/image). Download Extended Data 4, ZIP file. [file eneuro-12-ENEURO.0224-25.2025-s006.zip › Extended Data 4/wt1/L_img00125.jpeg]

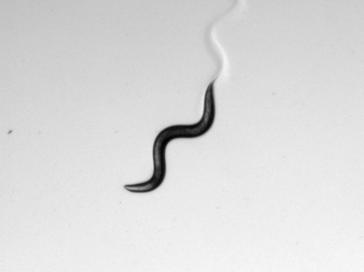

Supplement: Extended Data 4 — A sample WormTracker recording. This folder contains the recording of a wild-type worm (60 seconds, 15 frames per second), along with the associated stage file, time file, and a spline file generated by the Fit Spline module. The images were captured at 50% of the camera's resolution (4 KB/image). Download Extended Data 4, ZIP file. [file eneuro-12-ENEURO.0224-25.2025-s006.zip › Extended Data 4/wt1/L_img00126.jpeg]

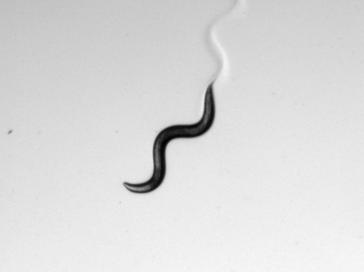

Supplement: Extended Data 4 — A sample WormTracker recording. This folder contains the recording of a wild-type worm (60 seconds, 15 frames per second), along with the associated stage file, time file, and a spline file generated by the Fit Spline module. The images were captured at 50% of the camera's resolution (4 KB/image). Download Extended Data 4, ZIP file. [file eneuro-12-ENEURO.0224-25.2025-s006.zip › Extended Data 4/wt1/L_img00127.jpeg]

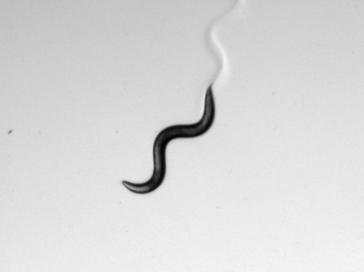

Supplement: Extended Data 4 — A sample WormTracker recording. This folder contains the recording of a wild-type worm (60 seconds, 15 frames per second), along with the associated stage file, time file, and a spline file generated by the Fit Spline module. The images were captured at 50% of the camera's resolution (4 KB/image). Download Extended Data 4, ZIP file. [file eneuro-12-ENEURO.0224-25.2025-s006.zip › Extended Data 4/wt1/L_img00128.jpeg]

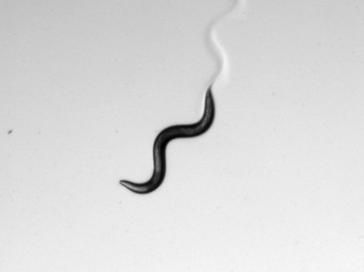

Supplement: Extended Data 4 — A sample WormTracker recording. This folder contains the recording of a wild-type worm (60 seconds, 15 frames per second), along with the associated stage file, time file, and a spline file generated by the Fit Spline module. The images were captured at 50% of the camera's resolution (4 KB/image). Download Extended Data 4, ZIP file. [file eneuro-12-ENEURO.0224-25.2025-s006.zip › Extended Data 4/wt1/L_img00129.jpeg]

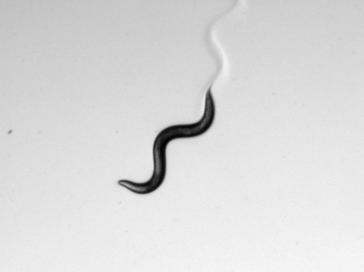

Supplement: Extended Data 4 — A sample WormTracker recording. This folder contains the recording of a wild-type worm (60 seconds, 15 frames per second), along with the associated stage file, time file, and a spline file generated by the Fit Spline module. The images were captured at 50% of the camera's resolution (4 KB/image). Download Extended Data 4, ZIP file. [file eneuro-12-ENEURO.0224-25.2025-s006.zip › Extended Data 4/wt1/L_img00130.jpeg]

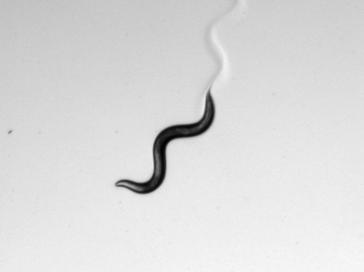

Supplement: Extended Data 4 — A sample WormTracker recording. This folder contains the recording of a wild-type worm (60 seconds, 15 frames per second), along with the associated stage file, time file, and a spline file generated by the Fit Spline module. The images were captured at 50% of the camera's resolution (4 KB/image). Download Extended Data 4, ZIP file. [file eneuro-12-ENEURO.0224-25.2025-s006.zip › Extended Data 4/wt1/L_img00131.jpeg]

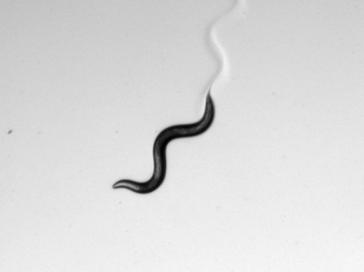

Supplement: Extended Data 4 — A sample WormTracker recording. This folder contains the recording of a wild-type worm (60 seconds, 15 frames per second), along with the associated stage file, time file, and a spline file generated by the Fit Spline module. The images were captured at 50% of the camera's resolution (4 KB/image). Download Extended Data 4, ZIP file. [file eneuro-12-ENEURO.0224-25.2025-s006.zip › Extended Data 4/wt1/L_img00132.jpeg]

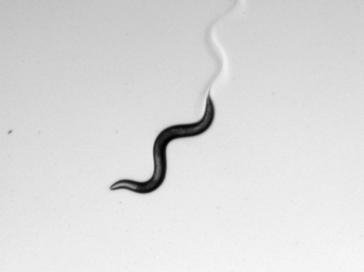

Supplement: Extended Data 4 — A sample WormTracker recording. This folder contains the recording of a wild-type worm (60 seconds, 15 frames per second), along with the associated stage file, time file, and a spline file generated by the Fit Spline module. The images were captured at 50% of the camera's resolution (4 KB/image). Download Extended Data 4, ZIP file. [file eneuro-12-ENEURO.0224-25.2025-s006.zip › Extended Data 4/wt1/L_img00133.jpeg]

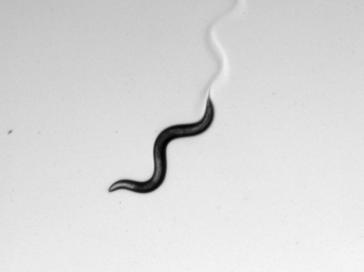

Supplement: Extended Data 4 — A sample WormTracker recording. This folder contains the recording of a wild-type worm (60 seconds, 15 frames per second), along with the associated stage file, time file, and a spline file generated by the Fit Spline module. The images were captured at 50% of the camera's resolution (4 KB/image). Download Extended Data 4, ZIP file. [file eneuro-12-ENEURO.0224-25.2025-s006.zip › Extended Data 4/wt1/L_img00134.jpeg]

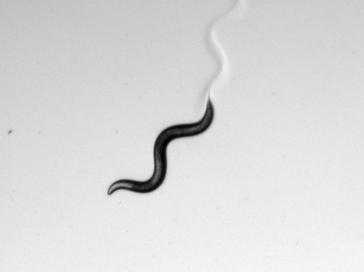

Supplement: Extended Data 4 — A sample WormTracker recording. This folder contains the recording of a wild-type worm (60 seconds, 15 frames per second), along with the associated stage file, time file, and a spline file generated by the Fit Spline module. The images were captured at 50% of the camera's resolution (4 KB/image). Download Extended Data 4, ZIP file. [file eneuro-12-ENEURO.0224-25.2025-s006.zip › Extended Data 4/wt1/L_img00135.jpeg]

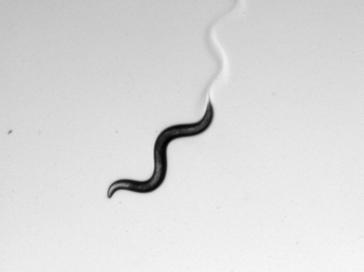

Supplement: Extended Data 4 — A sample WormTracker recording. This folder contains the recording of a wild-type worm (60 seconds, 15 frames per second), along with the associated stage file, time file, and a spline file generated by the Fit Spline module. The images were captured at 50% of the camera's resolution (4 KB/image). Download Extended Data 4, ZIP file. [file eneuro-12-ENEURO.0224-25.2025-s006.zip › Extended Data 4/wt1/L_img00136.jpeg]

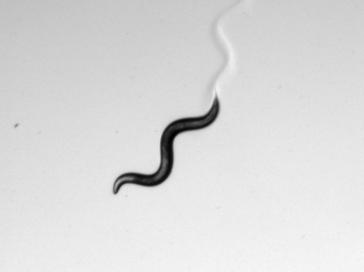

Supplement: Extended Data 4 — A sample WormTracker recording. This folder contains the recording of a wild-type worm (60 seconds, 15 frames per second), along with the associated stage file, time file, and a spline file generated by the Fit Spline module. The images were captured at 50% of the camera's resolution (4 KB/image). Download Extended Data 4, ZIP file. [file eneuro-12-ENEURO.0224-25.2025-s006.zip › Extended Data 4/wt1/L_img00137.jpeg]

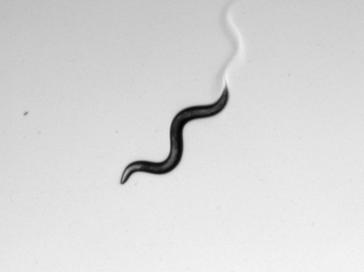

Supplement: Extended Data 4 — A sample WormTracker recording. This folder contains the recording of a wild-type worm (60 seconds, 15 frames per second), along with the associated stage file, time file, and a spline file generated by the Fit Spline module. The images were captured at 50% of the camera's resolution (4 KB/image). Download Extended Data 4, ZIP file. [file eneuro-12-ENEURO.0224-25.2025-s006.zip › Extended Data 4/wt1/L_img00138.jpeg]

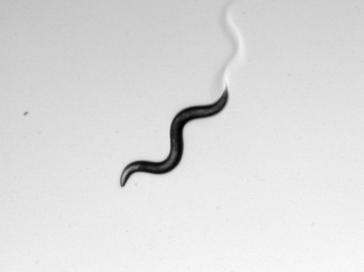

Supplement: Extended Data 4 — A sample WormTracker recording. This folder contains the recording of a wild-type worm (60 seconds, 15 frames per second), along with the associated stage file, time file, and a spline file generated by the Fit Spline module. The images were captured at 50% of the camera's resolution (4 KB/image). Download Extended Data 4, ZIP file. [file eneuro-12-ENEURO.0224-25.2025-s006.zip › Extended Data 4/wt1/L_img00139.jpeg]

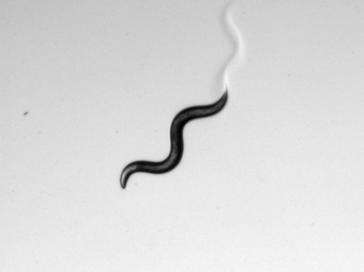

Supplement: Extended Data 4 — A sample WormTracker recording. This folder contains the recording of a wild-type worm (60 seconds, 15 frames per second), along with the associated stage file, time file, and a spline file generated by the Fit Spline module. The images were captured at 50% of the camera's resolution (4 KB/image). Download Extended Data 4, ZIP file. [file eneuro-12-ENEURO.0224-25.2025-s006.zip › Extended Data 4/wt1/L_img00140.jpeg]

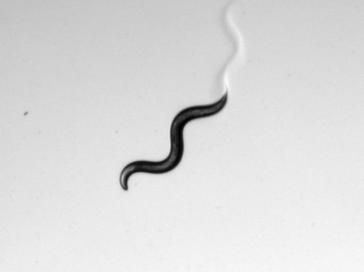

Supplement: Extended Data 4 — A sample WormTracker recording. This folder contains the recording of a wild-type worm (60 seconds, 15 frames per second), along with the associated stage file, time file, and a spline file generated by the Fit Spline module. The images were captured at 50% of the camera's resolution (4 KB/image). Download Extended Data 4, ZIP file. [file eneuro-12-ENEURO.0224-25.2025-s006.zip › Extended Data 4/wt1/L_img00141.jpeg]

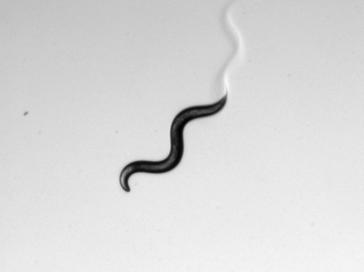

Supplement: Extended Data 4 — A sample WormTracker recording. This folder contains the recording of a wild-type worm (60 seconds, 15 frames per second), along with the associated stage file, time file, and a spline file generated by the Fit Spline module. The images were captured at 50% of the camera's resolution (4 KB/image). Download Extended Data 4, ZIP file. [file eneuro-12-ENEURO.0224-25.2025-s006.zip › Extended Data 4/wt1/L_img00142.jpeg]

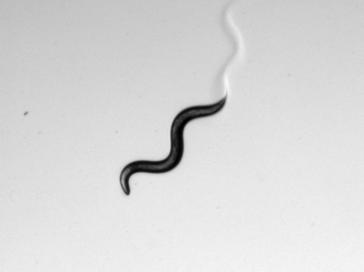

Supplement: Extended Data 4 — A sample WormTracker recording. This folder contains the recording of a wild-type worm (60 seconds, 15 frames per second), along with the associated stage file, time file, and a spline file generated by the Fit Spline module. The images were captured at 50% of the camera's resolution (4 KB/image). Download Extended Data 4, ZIP file. [file eneuro-12-ENEURO.0224-25.2025-s006.zip › Extended Data 4/wt1/L_img00143.jpeg]

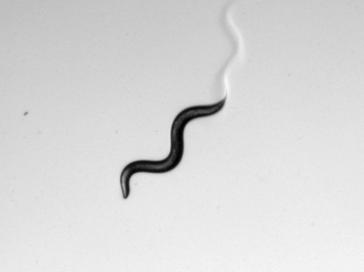

Supplement: Extended Data 4 — A sample WormTracker recording. This folder contains the recording of a wild-type worm (60 seconds, 15 frames per second), along with the associated stage file, time file, and a spline file generated by the Fit Spline module. The images were captured at 50% of the camera's resolution (4 KB/image). Download Extended Data 4, ZIP file. [file eneuro-12-ENEURO.0224-25.2025-s006.zip › Extended Data 4/wt1/L_img00144.jpeg]

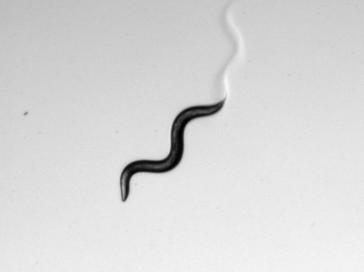

Supplement: Extended Data 4 — A sample WormTracker recording. This folder contains the recording of a wild-type worm (60 seconds, 15 frames per second), along with the associated stage file, time file, and a spline file generated by the Fit Spline module. The images were captured at 50% of the camera's resolution (4 KB/image). Download Extended Data 4, ZIP file. [file eneuro-12-ENEURO.0224-25.2025-s006.zip › Extended Data 4/wt1/L_img00145.jpeg]

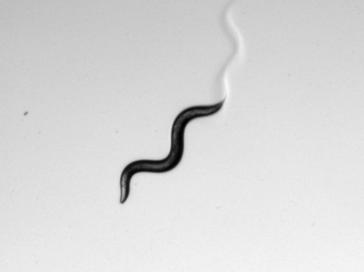

Supplement: Extended Data 4 — A sample WormTracker recording. This folder contains the recording of a wild-type worm (60 seconds, 15 frames per second), along with the associated stage file, time file, and a spline file generated by the Fit Spline module. The images were captured at 50% of the camera's resolution (4 KB/image). Download Extended Data 4, ZIP file. [file eneuro-12-ENEURO.0224-25.2025-s006.zip › Extended Data 4/wt1/L_img00146.jpeg]

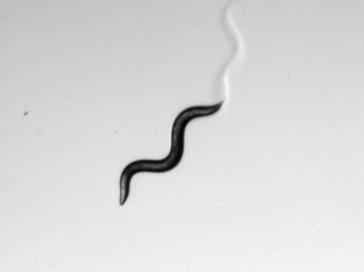

Supplement: Extended Data 4 — A sample WormTracker recording. This folder contains the recording of a wild-type worm (60 seconds, 15 frames per second), along with the associated stage file, time file, and a spline file generated by the Fit Spline module. The images were captured at 50% of the camera's resolution (4 KB/image). Download Extended Data 4, ZIP file. [file eneuro-12-ENEURO.0224-25.2025-s006.zip › Extended Data 4/wt1/L_img00147.jpeg]

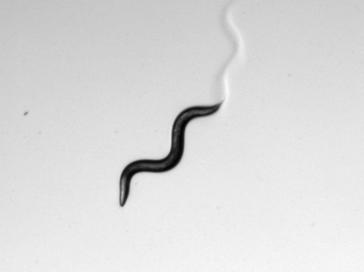

Supplement: Extended Data 4 — A sample WormTracker recording. This folder contains the recording of a wild-type worm (60 seconds, 15 frames per second), along with the associated stage file, time file, and a spline file generated by the Fit Spline module. The images were captured at 50% of the camera's resolution (4 KB/image). Download Extended Data 4, ZIP file. [file eneuro-12-ENEURO.0224-25.2025-s006.zip › Extended Data 4/wt1/L_img00148.jpeg]

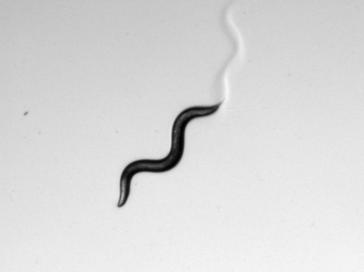

Supplement: Extended Data 4 — A sample WormTracker recording. This folder contains the recording of a wild-type worm (60 seconds, 15 frames per second), along with the associated stage file, time file, and a spline file generated by the Fit Spline module. The images were captured at 50% of the camera's resolution (4 KB/image). Download Extended Data 4, ZIP file. [file eneuro-12-ENEURO.0224-25.2025-s006.zip › Extended Data 4/wt1/L_img00149.jpeg]

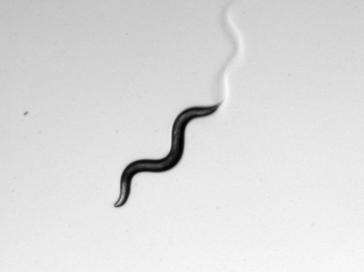

Supplement: Extended Data 4 — A sample WormTracker recording. This folder contains the recording of a wild-type worm (60 seconds, 15 frames per second), along with the associated stage file, time file, and a spline file generated by the Fit Spline module. The images were captured at 50% of the camera's resolution (4 KB/image). Download Extended Data 4, ZIP file. [file eneuro-12-ENEURO.0224-25.2025-s006.zip › Extended Data 4/wt1/L_img00150.jpeg]

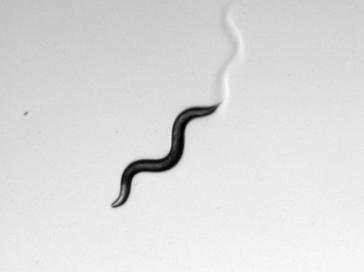

Supplement: Extended Data 4 — A sample WormTracker recording. This folder contains the recording of a wild-type worm (60 seconds, 15 frames per second), along with the associated stage file, time file, and a spline file generated by the Fit Spline module. The images were captured at 50% of the camera's resolution (4 KB/image). Download Extended Data 4, ZIP file. [file eneuro-12-ENEURO.0224-25.2025-s006.zip › Extended Data 4/wt1/L_img00151.jpeg]

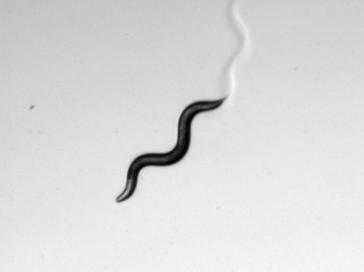

Supplement: Extended Data 4 — A sample WormTracker recording. This folder contains the recording of a wild-type worm (60 seconds, 15 frames per second), along with the associated stage file, time file, and a spline file generated by the Fit Spline module. The images were captured at 50% of the camera's resolution (4 KB/image). Download Extended Data 4, ZIP file. [file eneuro-12-ENEURO.0224-25.2025-s006.zip › Extended Data 4/wt1/L_img00152.jpeg]

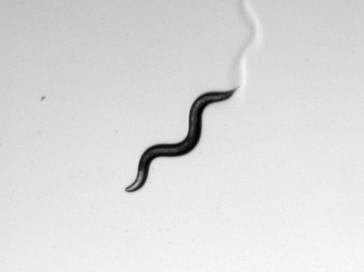

Supplement: Extended Data 4 — A sample WormTracker recording. This folder contains the recording of a wild-type worm (60 seconds, 15 frames per second), along with the associated stage file, time file, and a spline file generated by the Fit Spline module. The images were captured at 50% of the camera's resolution (4 KB/image). Download Extended Data 4, ZIP file. [file eneuro-12-ENEURO.0224-25.2025-s006.zip › Extended Data 4/wt1/L_img00153.jpeg]

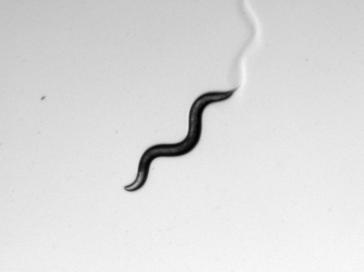

Supplement: Extended Data 4 — A sample WormTracker recording. This folder contains the recording of a wild-type worm (60 seconds, 15 frames per second), along with the associated stage file, time file, and a spline file generated by the Fit Spline module. The images were captured at 50% of the camera's resolution (4 KB/image). Download Extended Data 4, ZIP file. [file eneuro-12-ENEURO.0224-25.2025-s006.zip › Extended Data 4/wt1/L_img00154.jpeg]

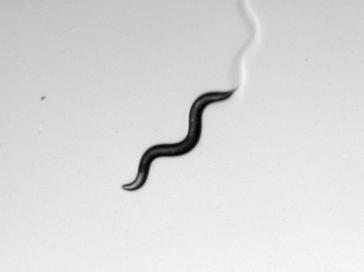

Supplement: Extended Data 4 — A sample WormTracker recording. This folder contains the recording of a wild-type worm (60 seconds, 15 frames per second), along with the associated stage file, time file, and a spline file generated by the Fit Spline module. The images were captured at 50% of the camera's resolution (4 KB/image). Download Extended Data 4, ZIP file. [file eneuro-12-ENEURO.0224-25.2025-s006.zip › Extended Data 4/wt1/L_img00155.jpeg]

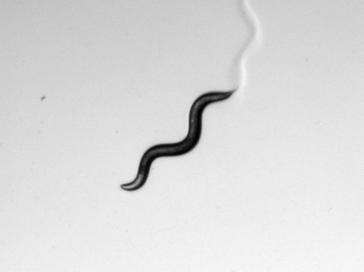

Supplement: Extended Data 4 — A sample WormTracker recording. This folder contains the recording of a wild-type worm (60 seconds, 15 frames per second), along with the associated stage file, time file, and a spline file generated by the Fit Spline module. The images were captured at 50% of the camera's resolution (4 KB/image). Download Extended Data 4, ZIP file. [file eneuro-12-ENEURO.0224-25.2025-s006.zip › Extended Data 4/wt1/L_img00156.jpeg]

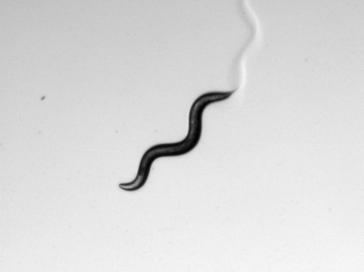

Supplement: Extended Data 4 — A sample WormTracker recording. This folder contains the recording of a wild-type worm (60 seconds, 15 frames per second), along with the associated stage file, time file, and a spline file generated by the Fit Spline module. The images were captured at 50% of the camera's resolution (4 KB/image). Download Extended Data 4, ZIP file. [file eneuro-12-ENEURO.0224-25.2025-s006.zip › Extended Data 4/wt1/L_img00157.jpeg]

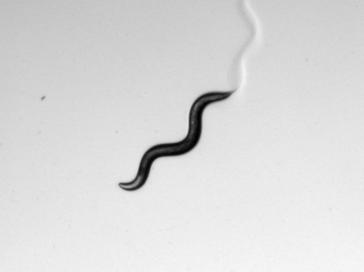

Supplement: Extended Data 4 — A sample WormTracker recording. This folder contains the recording of a wild-type worm (60 seconds, 15 frames per second), along with the associated stage file, time file, and a spline file generated by the Fit Spline module. The images were captured at 50% of the camera's resolution (4 KB/image). Download Extended Data 4, ZIP file. [file eneuro-12-ENEURO.0224-25.2025-s006.zip › Extended Data 4/wt1/L_img00158.jpeg]

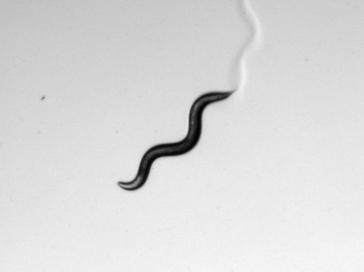

Supplement: Extended Data 4 — A sample WormTracker recording. This folder contains the recording of a wild-type worm (60 seconds, 15 frames per second), along with the associated stage file, time file, and a spline file generated by the Fit Spline module. The images were captured at 50% of the camera's resolution (4 KB/image). Download Extended Data 4, ZIP file. [file eneuro-12-ENEURO.0224-25.2025-s006.zip › Extended Data 4/wt1/L_img00159.jpeg]

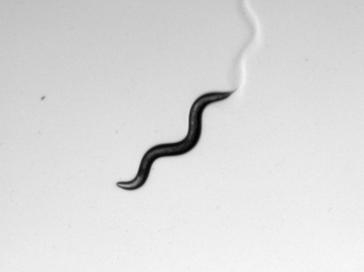

Supplement: Extended Data 4 — A sample WormTracker recording. This folder contains the recording of a wild-type worm (60 seconds, 15 frames per second), along with the associated stage file, time file, and a spline file generated by the Fit Spline module. The images were captured at 50% of the camera's resolution (4 KB/image). Download Extended Data 4, ZIP file. [file eneuro-12-ENEURO.0224-25.2025-s006.zip › Extended Data 4/wt1/L_img00160.jpeg]

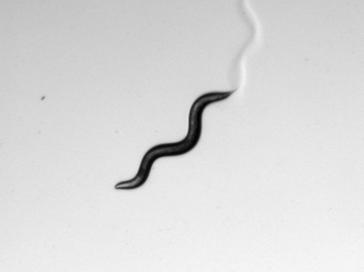

Supplement: Extended Data 4 — A sample WormTracker recording. This folder contains the recording of a wild-type worm (60 seconds, 15 frames per second), along with the associated stage file, time file, and a spline file generated by the Fit Spline module. The images were captured at 50% of the camera's resolution (4 KB/image). Download Extended Data 4, ZIP file. [file eneuro-12-ENEURO.0224-25.2025-s006.zip › Extended Data 4/wt1/L_img00161.jpeg]

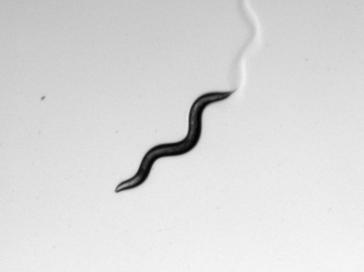

Supplement: Extended Data 4 — A sample WormTracker recording. This folder contains the recording of a wild-type worm (60 seconds, 15 frames per second), along with the associated stage file, time file, and a spline file generated by the Fit Spline module. The images were captured at 50% of the camera's resolution (4 KB/image). Download Extended Data 4, ZIP file. [file eneuro-12-ENEURO.0224-25.2025-s006.zip › Extended Data 4/wt1/L_img00162.jpeg]

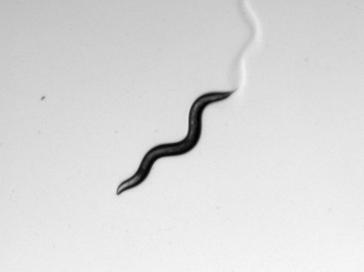

Supplement: Extended Data 4 — A sample WormTracker recording. This folder contains the recording of a wild-type worm (60 seconds, 15 frames per second), along with the associated stage file, time file, and a spline file generated by the Fit Spline module. The images were captured at 50% of the camera's resolution (4 KB/image). Download Extended Data 4, ZIP file. [file eneuro-12-ENEURO.0224-25.2025-s006.zip › Extended Data 4/wt1/L_img00163.jpeg]

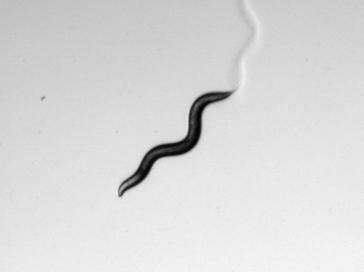

Supplement: Extended Data 4 — A sample WormTracker recording. This folder contains the recording of a wild-type worm (60 seconds, 15 frames per second), along with the associated stage file, time file, and a spline file generated by the Fit Spline module. The images were captured at 50% of the camera's resolution (4 KB/image). Download Extended Data 4, ZIP file. [file eneuro-12-ENEURO.0224-25.2025-s006.zip › Extended Data 4/wt1/L_img00164.jpeg]

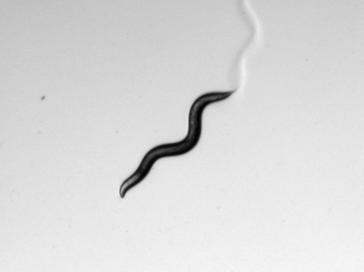

Supplement: Extended Data 4 — A sample WormTracker recording. This folder contains the recording of a wild-type worm (60 seconds, 15 frames per second), along with the associated stage file, time file, and a spline file generated by the Fit Spline module. The images were captured at 50% of the camera's resolution (4 KB/image). Download Extended Data 4, ZIP file. [file eneuro-12-ENEURO.0224-25.2025-s006.zip › Extended Data 4/wt1/L_img00165.jpeg]

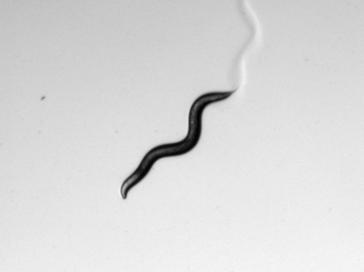

Supplement: Extended Data 4 — A sample WormTracker recording. This folder contains the recording of a wild-type worm (60 seconds, 15 frames per second), along with the associated stage file, time file, and a spline file generated by the Fit Spline module. The images were captured at 50% of the camera's resolution (4 KB/image). Download Extended Data 4, ZIP file. [file eneuro-12-ENEURO.0224-25.2025-s006.zip › Extended Data 4/wt1/L_img00166.jpeg]

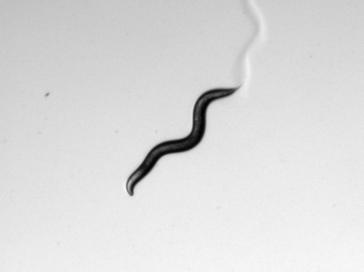

Supplement: Extended Data 4 — A sample WormTracker recording. This folder contains the recording of a wild-type worm (60 seconds, 15 frames per second), along with the associated stage file, time file, and a spline file generated by the Fit Spline module. The images were captured at 50% of the camera's resolution (4 KB/image). Download Extended Data 4, ZIP file. [file eneuro-12-ENEURO.0224-25.2025-s006.zip › Extended Data 4/wt1/L_img00167.jpeg]

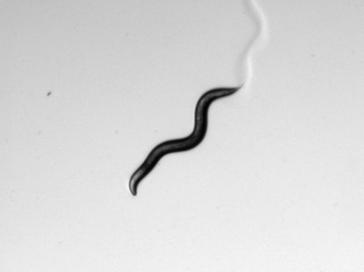

Supplement: Extended Data 4 — A sample WormTracker recording. This folder contains the recording of a wild-type worm (60 seconds, 15 frames per second), along with the associated stage file, time file, and a spline file generated by the Fit Spline module. The images were captured at 50% of the camera's resolution (4 KB/image). Download Extended Data 4, ZIP file. [file eneuro-12-ENEURO.0224-25.2025-s006.zip › Extended Data 4/wt1/L_img00168.jpeg]

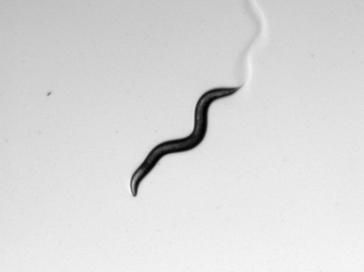

Supplement: Extended Data 4 — A sample WormTracker recording. This folder contains the recording of a wild-type worm (60 seconds, 15 frames per second), along with the associated stage file, time file, and a spline file generated by the Fit Spline module. The images were captured at 50% of the camera's resolution (4 KB/image). Download Extended Data 4, ZIP file. [file eneuro-12-ENEURO.0224-25.2025-s006.zip › Extended Data 4/wt1/L_img00169.jpeg]

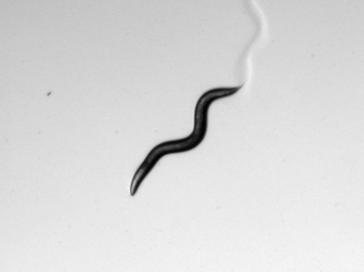

Supplement: Extended Data 4 — A sample WormTracker recording. This folder contains the recording of a wild-type worm (60 seconds, 15 frames per second), along with the associated stage file, time file, and a spline file generated by the Fit Spline module. The images were captured at 50% of the camera's resolution (4 KB/image). Download Extended Data 4, ZIP file. [file eneuro-12-ENEURO.0224-25.2025-s006.zip › Extended Data 4/wt1/L_img00170.jpeg]

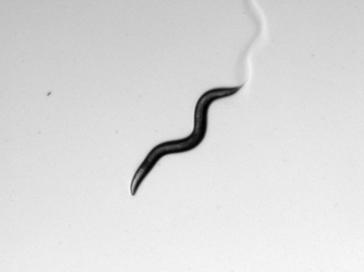

Supplement: Extended Data 4 — A sample WormTracker recording. This folder contains the recording of a wild-type worm (60 seconds, 15 frames per second), along with the associated stage file, time file, and a spline file generated by the Fit Spline module. The images were captured at 50% of the camera's resolution (4 KB/image). Download Extended Data 4, ZIP file. [file eneuro-12-ENEURO.0224-25.2025-s006.zip › Extended Data 4/wt1/L_img00171.jpeg]

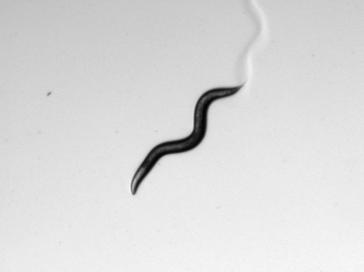

Supplement: Extended Data 4 — A sample WormTracker recording. This folder contains the recording of a wild-type worm (60 seconds, 15 frames per second), along with the associated stage file, time file, and a spline file generated by the Fit Spline module. The images were captured at 50% of the camera's resolution (4 KB/image). Download Extended Data 4, ZIP file. [file eneuro-12-ENEURO.0224-25.2025-s006.zip › Extended Data 4/wt1/L_img00172.jpeg]

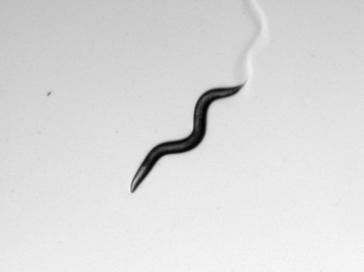

Supplement: Extended Data 4 — A sample WormTracker recording. This folder contains the recording of a wild-type worm (60 seconds, 15 frames per second), along with the associated stage file, time file, and a spline file generated by the Fit Spline module. The images were captured at 50% of the camera's resolution (4 KB/image). Download Extended Data 4, ZIP file. [file eneuro-12-ENEURO.0224-25.2025-s006.zip › Extended Data 4/wt1/L_img00173.jpeg]

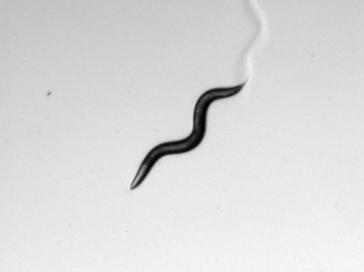

Supplement: Extended Data 4 — A sample WormTracker recording. This folder contains the recording of a wild-type worm (60 seconds, 15 frames per second), along with the associated stage file, time file, and a spline file generated by the Fit Spline module. The images were captured at 50% of the camera's resolution (4 KB/image). Download Extended Data 4, ZIP file. [file eneuro-12-ENEURO.0224-25.2025-s006.zip › Extended Data 4/wt1/L_img00174.jpeg]

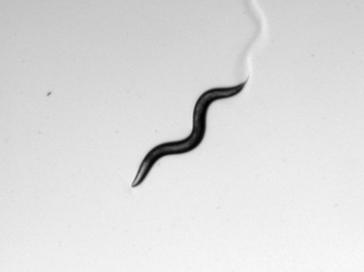

Supplement: Extended Data 4 — A sample WormTracker recording. This folder contains the recording of a wild-type worm (60 seconds, 15 frames per second), along with the associated stage file, time file, and a spline file generated by the Fit Spline module. The images were captured at 50% of the camera's resolution (4 KB/image). Download Extended Data 4, ZIP file. [file eneuro-12-ENEURO.0224-25.2025-s006.zip › Extended Data 4/wt1/L_img00175.jpeg]

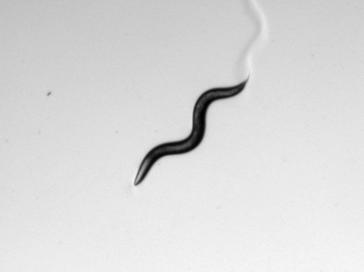

Supplement: Extended Data 4 — A sample WormTracker recording. This folder contains the recording of a wild-type worm (60 seconds, 15 frames per second), along with the associated stage file, time file, and a spline file generated by the Fit Spline module. The images were captured at 50% of the camera's resolution (4 KB/image). Download Extended Data 4, ZIP file. [file eneuro-12-ENEURO.0224-25.2025-s006.zip › Extended Data 4/wt1/L_img00176.jpeg]

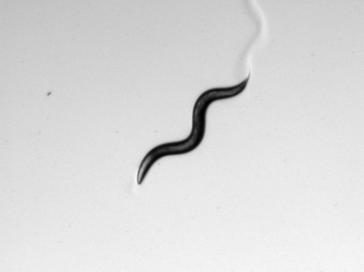

Supplement: Extended Data 4 — A sample WormTracker recording. This folder contains the recording of a wild-type worm (60 seconds, 15 frames per second), along with the associated stage file, time file, and a spline file generated by the Fit Spline module. The images were captured at 50% of the camera's resolution (4 KB/image). Download Extended Data 4, ZIP file. [file eneuro-12-ENEURO.0224-25.2025-s006.zip › Extended Data 4/wt1/L_img00177.jpeg]

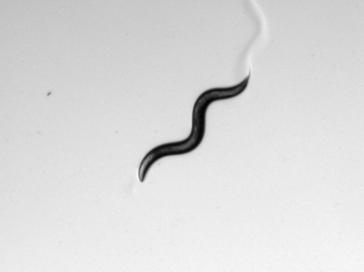

Supplement: Extended Data 4 — A sample WormTracker recording. This folder contains the recording of a wild-type worm (60 seconds, 15 frames per second), along with the associated stage file, time file, and a spline file generated by the Fit Spline module. The images were captured at 50% of the camera's resolution (4 KB/image). Download Extended Data 4, ZIP file. [file eneuro-12-ENEURO.0224-25.2025-s006.zip › Extended Data 4/wt1/L_img00178.jpeg]

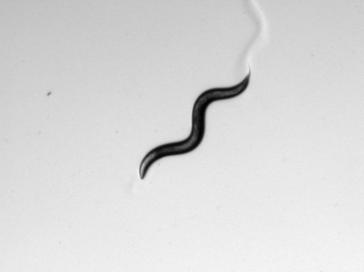

Supplement: Extended Data 4 — A sample WormTracker recording. This folder contains the recording of a wild-type worm (60 seconds, 15 frames per second), along with the associated stage file, time file, and a spline file generated by the Fit Spline module. The images were captured at 50% of the camera's resolution (4 KB/image). Download Extended Data 4, ZIP file. [file eneuro-12-ENEURO.0224-25.2025-s006.zip › Extended Data 4/wt1/L_img00179.jpeg]

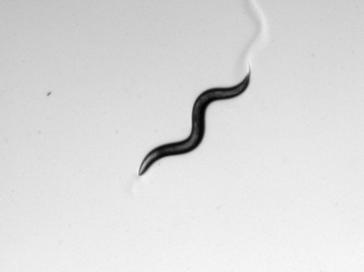

Supplement: Extended Data 4 — A sample WormTracker recording. This folder contains the recording of a wild-type worm (60 seconds, 15 frames per second), along with the associated stage file, time file, and a spline file generated by the Fit Spline module. The images were captured at 50% of the camera's resolution (4 KB/image). Download Extended Data 4, ZIP file. [file eneuro-12-ENEURO.0224-25.2025-s006.zip › Extended Data 4/wt1/L_img00180.jpeg]

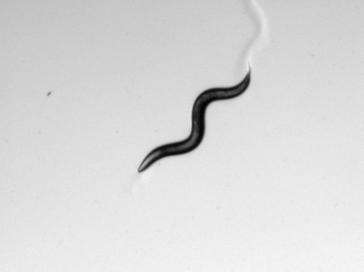

Supplement: Extended Data 4 — A sample WormTracker recording. This folder contains the recording of a wild-type worm (60 seconds, 15 frames per second), along with the associated stage file, time file, and a spline file generated by the Fit Spline module. The images were captured at 50% of the camera's resolution (4 KB/image). Download Extended Data 4, ZIP file. [file eneuro-12-ENEURO.0224-25.2025-s006.zip › Extended Data 4/wt1/L_img00181.jpeg]

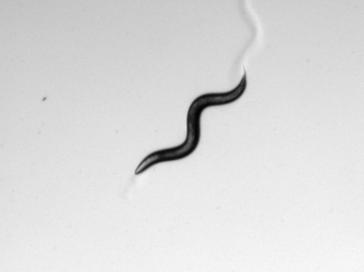

Supplement: Extended Data 4 — A sample WormTracker recording. This folder contains the recording of a wild-type worm (60 seconds, 15 frames per second), along with the associated stage file, time file, and a spline file generated by the Fit Spline module. The images were captured at 50% of the camera's resolution (4 KB/image). Download Extended Data 4, ZIP file. [file eneuro-12-ENEURO.0224-25.2025-s006.zip › Extended Data 4/wt1/L_img00182.jpeg]

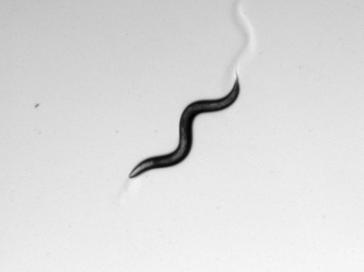

Supplement: Extended Data 4 — A sample WormTracker recording. This folder contains the recording of a wild-type worm (60 seconds, 15 frames per second), along with the associated stage file, time file, and a spline file generated by the Fit Spline module. The images were captured at 50% of the camera's resolution (4 KB/image). Download Extended Data 4, ZIP file. [file eneuro-12-ENEURO.0224-25.2025-s006.zip › Extended Data 4/wt1/L_img00183.jpeg]
