# Supplementary material for: Track-A-Worm 2.0: A Software Suite for Quantifying Properties of C. elegans Locomotion, Bending, Sleep, and Action Potentials
Source: eNeuro. 2025 Aug 13;12(8):ENEURO.0224-25.2025. doi: 10.1523/ENEURO.0224-25.2025 (PMC12393025; doi:10.1523/ENEURO.0224-25.2025)
Supplement: Extended Data 5 — A sample SleepTracker recording. This folder contains the recording of four wild-type worms (10 hrs., 1 frame/10 sec) along with the associated times file. Due to its large size, this recording is stored on the Zenodo server (https://zenodo.org/records/15857492) for download. Download Extended Data 5, DOCX file. [file eneuro-12-ENEURO.0224-25.2025-s007.docx]

**Extended Data 5**

Due to its large size, this recording is stored on the Zenodo server (<https://zenodo.org/records/15857492>) for download.
